# Mitophagy mitigates mitochondrial DNA-induced activation of cGAS-STING in autoimmune thyroiditis

**DOI:** 10.1038/s41467-026-76047-9

**Published:** 2026-07-24

**Authors:** Xiao-Chen Xie, Yang Guo, Ran Guo, Yong-Ze Li, Shan-Shan Wang, Xiao-You Jiang, Shuang Hao, Ye Zhang, Yu-Han Li, Xi-Yan Liu, Xiao-Xu Wu, Xin-Yue Zhang, Wen-Dong Guo, Yan-Ling Feng, Jia-Bin Li, Chen Liu, Liang Wang, Zhen-Hua Li, Wei-Ping Teng, Zhong-Yan Shan, Liu Cao, Qi-Qiang Guo

**Affiliations:** 1 Department of Endocrinology and Metabolism, Institute of Endocrinology, NHC Key Laboratory of Diagnosis and Treatment of Thyroid Diseases, The First Affiliated Hospital of China Medical University, Shenyang, Liaoning Province China; 2 The College of Basic Medical Science, Health Sciences Institute, China Medical University, Shenyang, Liaoning Province China; 3 Key Laboratory of Cell Biology of Ministry of Public Health, Key Laboratory of Medical Cell Biology of Ministry of Education, Key Laboratory of Precision Diagnosis and Treatment of Gastrointestinal Tumors of Ministry of Education, Liaoning Province Collaborative Innovation Center of Aging Related Disease Diagnosis and Treatment and Prevention, China Medical University, Shenyang, Liaoning Province China; 4 Department of Orthopedics, Shengjing Hospital of China Medical University, Shenyang, Liaoning Province China; 5 Department of Cell Biology, Key Laboratory of Cell Biology, National Health Commission of the PR China, Key Laboratory of Medical Cell Biology, Ministry of Education of the PR China, China Medical University, Shenyang, Liaoning Province China; 6 Department of Pathology, College of Basic Medical Sciences, China Medical University, Shenyang, Liaoning Province China; 7 School of Pharmacy, China Medical University, Shenyang, Liaoning Province China; 8 Clinical Translational Research Center, Shengjing Hospital of China Medical University, Shenyang, Liaoning Province China

**Keywords:** Thyroid diseases, Mitophagy, Mechanisms of disease

## Abstract

Autoimmune thyroiditis arises from disrupted homeostasis of thyroid follicular epithelial cells and coordinated immune cell activation within the microenvironment. However, its pathogenesis is not fully understood. Here, we identify a mitochondrial (mt) DNA-cGAS-STING inflammatory axis as a driver of autoimmune thyroiditis in mice. By contrast, ubiquitin-dependent mitophagy mediated by PINK1 and Parkin was found to protect mice from disease. Mechanistically, mitochondrial dysfunction elevates mitochondrial reactive oxygen species levels, activating the ATM-CHK2 DNA damage response pathway, which in turn phosphorylates the autophagy adapter TAX1BP1 at Ser722. This modification promotes the recruitment of mitochondria to autophagosomes, thereby facilitating mitophagy. Impairing the ATM-CHK2-TAX1BP1 mitophagy pathway causes mtDNA leakage into the cytosol and triggers cGAS-STING-dependent inflammation. Notably, pharmacological inhibition of STING with C176 effectively slows autoimmune thyroiditis progression. Together, these findings define an mtDNA-driven pathogenic mechanism in autoimmune thyroiditis and identify STING as a potential therapeutic target.

## Abstract (web summary)

In this study, the authors demonstrate that mitochondrial DNA leakage activates the cGAS-STING pathway in autoimmune thyroiditis and that restoring mitophagy or pharmacologically inhibiting STING reduces disease severity in mice.

## Introduction

Autoimmune thyroiditis (AIT) is a common endocrine disorder marked by destruction of thyroid follicles, lymphocytic infiltration, and concurrent hypothyroidism. Evidence indicates that AIT is driven primarily by the disrupted homeostasis of thyroid follicular epithelial cells, mediated by the coordinated activity of immune cells within the local microenvironment^1^. However, the pathogenic mechanisms underlying AIT have not been fully elucidated.

The synthesis of thyroid hormones in follicular epithelial cells depends on hydrogen peroxide to oxidize absorbed iodide into active iodine, enabling the iodination of tyrosine residues on thyroglobulin and subsequent hormone coupling. As a result, these cells are persistently exposed to reactive oxygen species (ROS), leading to chronic oxidative stress^2^. Oxidative stress can induce mitochondrial damage and dysfunction, a common hallmark across many diseases. Owing to their endosymbiotic origin, mitochondria retain bacterial-like characteristics, and damage triggers the cytosolic release of damage-associated molecular patterns (DAMPs) such as mitochondrial DNA (mtDNA) and ROS. Failure to properly control these DAMPs can drive autoimmune and inflammatory pathology^3^. The cyclic GMP-AMP synthase (cGAS)-stimulator of interferon genes (STING) pathway, a key innate immune signaling cascade, is activated by cytosolic mtDNA and induces proinflammatory cytokines such as interleukin-6 (IL-6), tumor necrosis factor (TNF), interleukin-1β (IL-1β), and interferon gamma (IFNγ)^4,5^. The pivotal role of the cGAS-STING signaling pathway has been demonstrated in autoimmune diseases such as systemic lupus erythematosus (SLE)^6–8^. Recent studies have also implicated this pathway in autoimmune thyroiditis, with STING expression elevated in Hashimoto’s thyroiditis tissues correlating with inflammatory cytokine levels^9^ and cGAS-STING activation promoting thyrocyte pyroptosis and immune cell infiltration^10^. Whether mitochondrial dysfunction substantially contributes to the pathogenesis of autoimmune thyroiditis and whether targeting this pathway offers therapeutic value remains unknown.

Against this backdrop, mitophagy emerges as a key protective mechanism that limits mitochondrial dysfunction and its proinflammatory consequences. PINK1/Parkin-dependent mitophagy is a selective form of autophagy that degrades damaged mitochondria through the autophagy-lysosome system to maintain mitochondrial homeostasis^11^. As a key stress-adaptive pathway, mitophagy safeguards the mitochondrial network and can be triggered by loss of mitochondrial membrane potential^12,13^, mitochondrial protein misfolding^14^, or mitochondrial ROS (mtROS)^15–17^. Central to this process is the stabilization of PINK1 on the outer mitochondrial membrane (OMM), which recruits the E3 ubiquitin ligase Parkin and phosphorylates both Parkin at Ser65 and ubiquitin at Ser65^18,19^. Activated Parkin results in the ubiquitylation of OMM proteins on damaged mitochondria. Targeting ubiquitinated mitochondria to autophagosomes is a crucial step in mitophagy that requires selective autophagy adaptors, including OPTN, NDP52, TAX1BP1, and p62. These adaptors recognize ubiquitinated mitochondria through their ubiquitin-binding domains (UBDs) and interact with the LC3 protein via the LC3-interacting region (LIR) motif^16,20–24^. Notably, loss of Pink1 drives Hashimoto’s thyroiditis in a mouse model, accompanied by mitochondrial abnormalities, mitophagy defects, and upregulation of DAMP-related genes, including STING and NLRP3, leading to increased release of pro-inflammatory cytokines and immune cell recruitment^25^. These findings strongly suggest that impaired mitophagy contributes to the pathogenesis of autoimmune thyroiditis. However, the upstream signaling mechanisms that regulate mitophagy in thyroid follicular epithelial cells under chronic oxidative stress remain unexplored.

Another pathway shown to affect mitophagy under oxidative stress is the ATM-CHK2 DNA damage response. The DNA damage response (DDR) ATM-CHK2 signaling pathway is essential for maintaining genomic stability within the nucleus^26^. Surprisingly, ATM deficiency is associated with several neurodegenerative diseases characterized by mitochondrial dysfunction and increased ROS production, suggesting a critical role for ATM in cytoplasmic organelle quality control^27^. Recent studies have focused on the ATM pathway’s role as a ROS sensor^28^, regulating autophagy to maintain homeostasis of both mitochondria and peroxisomes^17,29^. Our prior research showed that, in response to mtROS, activated CHK2 translocates to damaged mitochondria, initiating PINK1-Parkin-dependent mitophagy and targeting compromised mitochondria for clearance^16^. However, under the chronic oxidative stress conditions experienced by thyroid follicular epithelial cells during iodine-dependent hormone synthesis, whether this pathway participates in mitochondrial quality control, and the specific regulatory mechanisms involved, remain to be explored.

In this study, we aimed to investigate whether mitochondrial dysfunction-driven activation of the mtDNA-cGAS-STING inflammatory axis contributes to the pathogenesis of autoimmune thyroiditis and to elucidate the upstream regulatory mechanism of mitophagy that counteracts this process. We hypothesized that in thyroid follicular epithelial cells, chronic oxidative stress associated with thyroid hormone synthesis leads to mitochondrial damage and mtDNA leakage into the cytosol, thereby activating the cGAS-STING pathway and promoting autoimmune inflammation. Furthermore, we hypothesized that the ATM-CHK2-TAX1BP1 axis activates PINK1/Parkin-dependent mitophagy to clear damaged mitochondria and prevent mtDNA release, thus protecting against disease progression. Finally, we tested whether pharmacological inhibition of STING could attenuate autoimmune thyroiditis in a mouse model.

## Results

### Mitochondrial dysfunction and elevated mitophagy in autoimmune thyroiditis

**Fig. 1:**
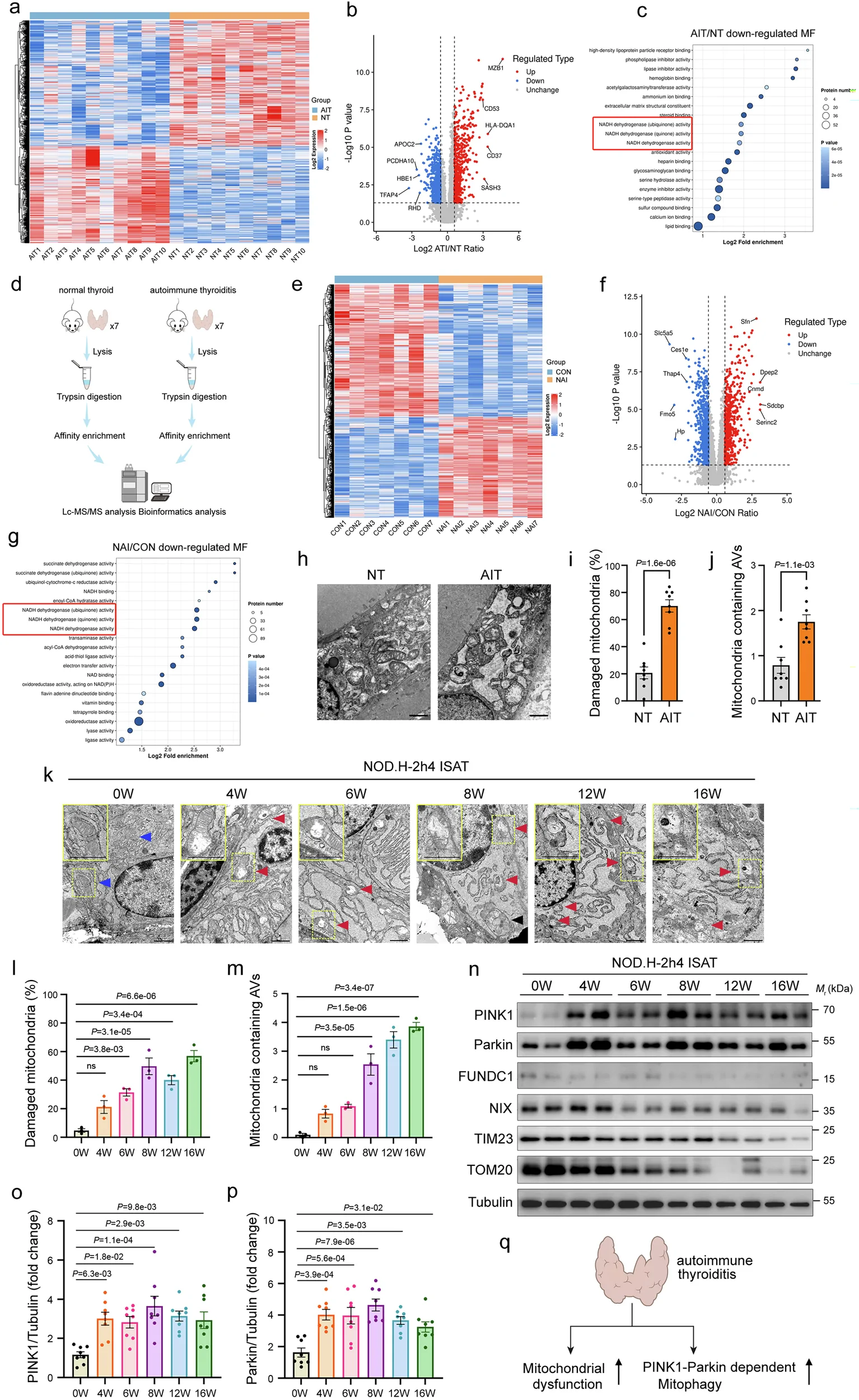
Mitochondrial dysfunction and elevated mitophagy in autoimmune thyroiditis. **a** Protein expression heatmap for thyroid tissue comparing autoimmune thyroiditis and healthy controls. Lines indicate individual protein assays quantified by mass spectrometry. **b** Volcano plot showing protein fold changes versus *P*-values in thyroid tissue from autoimmune thyroiditis patients and healthy controls. **c** Molecular Function (MF) enrichment analysis in thyroid tissue for downregulated proteins comparing autoimmune thyroiditis patients with healthy controls. Bubble color reflects Fisher’s exact test *P*-value, and bubble size reflects the number of proteins. **d** Workflow chart outlining LC-MS/MS analysis of thyroid tissue proteome profiles from high-iodine-fed and normal animals (*n* = 7 per group). **e** Thyroid tissue protein expression heatmaps for high-iodine-fed versus normal mice. **f** Volcano plot as in (**b**) for mice (NAI vs CON). **g** MF enrichment for downregulated proteins in mice. **h** Representative electron microscope images of thyroid tissue from patients with autoimmune thyroiditis and healthy controls. Scale bar, 1 µm. **i**, **j** Levels of damaged mitochondria (**i**) and mitochondria containing autophagic vacuoles (AVs) (**j**) in thyroid tissue assessed by TEM. **k** Representative transmission electron microscopy images of mouse thyroid tissue from the indicated groups. Red arrows indicate damaged mitochondria, blue arrows indicate intact mitochondria, and black arrows indicate autophagosomes engulfing damaged mitochondria. Scale bar, 1 µm. **l**, **m** Quantification of damaged mitochondria (**l**) and AV‑containing mitochondria (**m**) in mice over time (0–16 w). Cells counted: **l** *n* = 37,42,55,50,35,39; **m** *n* = 31,41,45,49,37,38 per time point. Variations in cell numbers are due to technical variability in TEM sample preparation; importantly, cells at each time point were derived from three independent mice. **n**–**p** Relative expression of PINK1 and Parkin in thyroid tissue assessed by western blot. Tubulin served as the loading control. *n* = 8 in each group. **q** A schematic model of autoimmune thyroiditis associated with mitochondrial dysfunction and enhanced PINK1-Parkin-dependent mitophagy. Statistics: Data are mean ± SEM. Two‑sided Student’s *t*‑test for two groups (**b**, **f**, **i**, **j**); one‑way ANOVA with Tukey’s post‑hoc test for multiple groups (**l**, **m**, **n**–**p**). Exact *P*-values are shown in graphs; ns indicates not significant.

To investigate the pathogenesis of autoimmune thyroiditis, we first analyzed differential protein expression patterns. We compared proteomic profiles of thyroid tissue from patients with autoimmune thyroiditis to healthy controls, and of lesional thyroid tissue to genetically matched control tissue in the NOD.H-2^h4^ mouse model of autoimmune thyroiditis^30^ (Fig. 1a–g, Supplementary Figs. 1–3). In both cases, the proteomes recapitulated hallmark features of autoimmune thyroid disease (Supplementary Figs. 1g, 2g) and indicated immune cell activation (Supplementary Figs. 1h, 2h). Most notably, coordinated suppression of mitochondrial respiratory chain activity (Fig. 1c, g), together with evidence of accumulating mitochondrial damage, was observed (Fig. 1h–m). Furthermore, in vitro experiments showed that Nthy-ori3-1 thyroid follicular epithelial cells exposed to excess iodine developed exacerbated mitochondrial damage, increased mitochondrial ROS, and reduced ATP production. Crucially, blocking mtROS transport between mitochondria and the cytosol with 4,4’diisothiocyanatostilbene-2,2’-disulfonate (DIDS), an inhibitor of the outer membrane pore VDAC1, rescued these mitochondrial impairments (Supplementary Fig. 4), indicating that ROS generated during thyroid hormone synthesis in follicular epithelial cells directly contributes to mitochondrial dysfunction. Collectively, these multi-model analyses identify mitochondrial dysfunction as a pathogenic hub in the development of autoimmune thyroiditis.

In autoimmune thyroiditis, mitochondrial damage and dysfunction coincide with elevated mitophagy levels (Fig. 1h–m). Mitophagy maintains mitochondrial quality control and cellular homeostasis by removing dysfunctional mitochondria. To investigate the mechanism of mitophagy induction, we used the iodine-accelerated spontaneous autoimmune thyroiditis (ISAT) model. Disease-associated changes included reduced levels of the mitochondrial proteins TOM20 and TIM23, indicative of mitophagic activation, along with upregulation of PINK1 and Parkin, while the mitophagy receptors FUNDC1 and NIX remained unchanged (Fig. 1n–p, Supplementary Fig. 5). These findings implicate the PINK1-Parkin pathway in triggering mitophagy. Consistently, excess iodine induced mitophagy in thyroid follicular epithelial cells in vitro (Supplementary Fig. 6). Collectively, these data support a model in which PINK1-Parkin-dependent mitophagy acts as an adaptive response to mitochondrial dysfunction and damage during the pathogenesis of autoimmune thyroiditis (Fig. 1q).

### cGAS/STING pathway activation correlates with clinical progression in autoimmune thyroiditis

The cGAS-STING signaling pathway is the core machinery for sensing and responding to aberrant cytosolic double-stranded DNA (dsDNA). Beyond pathogen-derived exogenous DNA released during infection, mitochondrial damage and genomic instability can generate mislocalized self-dsDNA. Such leaked endogenous DNA is detected by the DNA sensor cGAS, initiating cGAS-STING signaling. Dysregulated activation of this pathway is causally implicated in a range of autoimmune and autoinflammatory diseases. Notably, our proteomics analyses revealed elevated STING expression in autoimmune thyroiditis, and further investigation in both the ISAT mouse model and thyroid tissues from AIT patients showed increased release of mtDNA into the cytosol (Supplementary Fig. 7), implicating cGAS-STING pathway activation in disease progression.

**Fig. 2:**
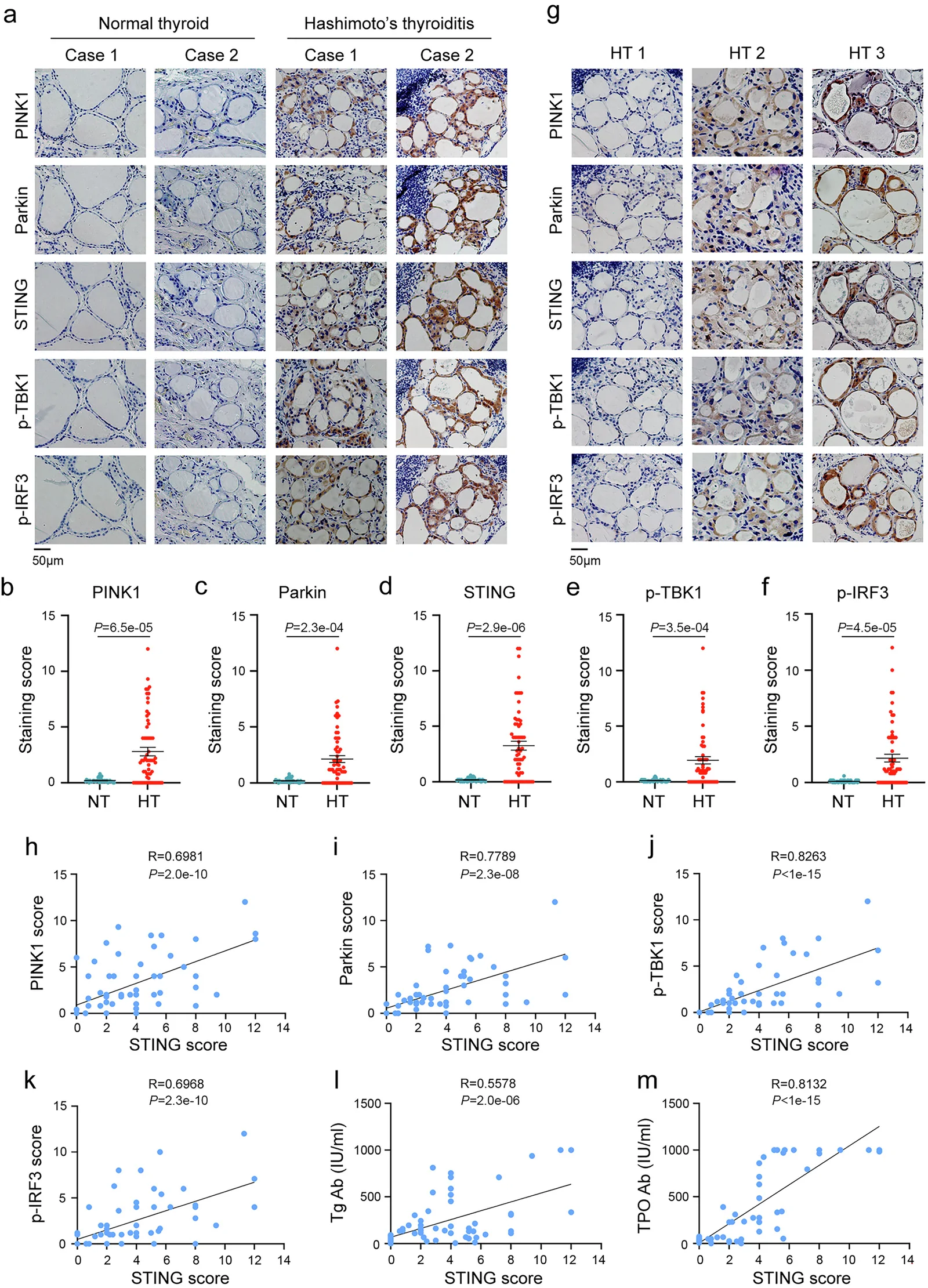
cGAS/STING pathway activation correlates with clinical progression in autoimmune thyroiditis. **a** IHC staining of Hashimoto’s thyroiditis (HT) and control thyroid samples with the indicated antibodies. Representative images from two cases are shown. Scale bar, 50 µm. **b**–**f** Comparison of staining scores for the indicated proteins between HT (*n* = 63) and control thyroid samples (*n* = 40). Data are mean ± SEM, comparisons between two groups were performed using a two-sided Mann-Whitney U test, adjusted *P*-values are shown. **g** Human thyroid samples with HT were stained using the indicated antibodies. Representative images are shown. Scale bar, 50 µm. **h**–**m** IHC staining of HT thyroid samples with the indicated antibodies was scored, and correlation analyses were performed. A Spearman correlation test was used (two-tailed).

We investigated the clinical relevance of cGAS-STING signaling and PINK1-Parkin-dependent mitophagy in Hashimoto’s thyroiditis (HT) by performing immunohistochemical staining of key molecular markers (STING, p-TBK1, p-IRF3, PINK1, and Parkin) in 63 HT thyroid specimens and 40 normal controls. HT specimens exhibited significant upregulation of STING, p-TBK1, p-IRF3, PINK1, and Parkin (Fig. 2a–f). Positive staining was restricted to thyrofollicular epithelial cells and absent in infiltrating lymphocytes or stromal immune cells. Quantitative analysis revealed strong positive correlations (all *P* < 0.001) between STING expression and downstream effectors (p-TBK1: *R* = 0.8263; p-IRF3: *R* = 0.6968), mitophagy regulators (PINK1: *R* = 0.6981; Parkin: *R* = 0.7789), and clinical progression markers (TPO-Ab: *R* = 0.8132; TgAb: *R* = 0.5578) (Fig. 2g–m). These findings collectively demonstrate that cGAS-STING pathway activation within thyroid epithelial cells is mechanistically linked to HT pathogenesis, while concurrent upregulation of PINK1-Parkin-mediated mitophagy likely reflects a compensatory response mitigating epithelial damage during disease progression.

### Activation of PINK1/Parkin-mediated mitophagy suppresses autoimmune thyroiditis pathogenesis

**Fig. 3:**
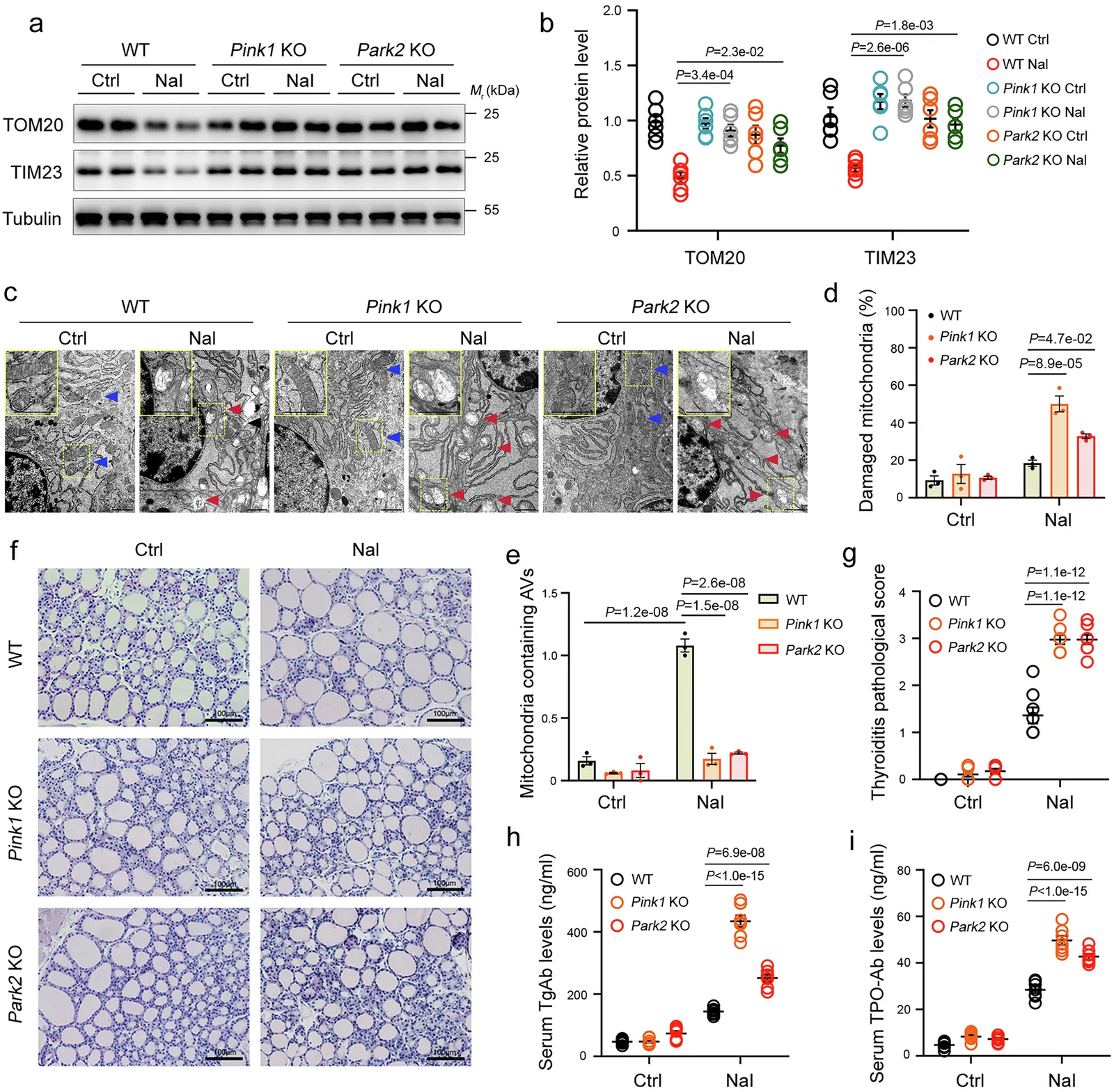
Activation of PINK1/Parkin-mediated mitophagy suppresses autoimmune thyroiditis pathogenesis. **a**, **b** Western blot analysis of TOM20 and TIM23 in thyroids from WT, *Pink1* KO, and *Park2* KO mice, comparing control versus ISAT groups. Tubulin was used as a loading control. Data are presented as mean ± SEM (*n* = 6 in each group); Comparisons were performed using two-way ANOVA followed by Tukey’s multiple comparisons test (two-sided). Adjusted *P*-values are shown. **c** Representative transmission electron microscopy images of mouse thyroid tissue from the indicated groups. Red arrows indicate damaged mitochondria (characterized by disrupted cristae and reduced cristae density), blue arrows indicate intact mitochondria, and black arrows indicate autophagosomes engulfing damaged mitochondria. Scale bar, 1 µm. **d**, **e** Levels of damaged mitochondria (**d**) and mitochondria containing AVs (**e**) in thyroid tissue assessed by TEM. Data are presented as mean ± SEM (a total of 48 cells were counted per group from three independent mice); Multiple comparisons were performed using two-way ANOVA followed by Tukey’s multiple comparisons test (two-sided). Adjusted *P*-values are shown. **f**, **g** Inflammation in the thyroid was quantified from H&E-stained paraffin sections by measuring the area of lymphocyte infiltration. Scale bar, 100 µm. Thyroid inflammation scores are presented. Data are presented as mean ± SEM (*n* = 8 in each group); Multiple comparisons were performed using two-way ANOVA followed by Tukey’s multiple comparisons test (two-sided). Adjusted *P*-values are shown. **h**, **i** TgAb and TPO-Ab levels were measured by ELISA. Data are presented as mean ± SEM (*n* = 7 in each group); Multiple comparisons were performed using two-way ANOVA followed by Tukey’s multiple comparisons test (two-sided). Adjusted *P*-values are shown.

We next investigated the role of PINK1/Parkin-mediated mitophagy in autoimmune thyroiditis. We established an iodine-induced spontaneous autoimmune thyroiditis (ISAT) model in NOD.H-2^h4^ mice and compared disease progression across wild-type (WT), *Pink1*^*−/−*^, and *Park2*^*−/−*^ ISAT mice. Western blotting of thyroid tissues revealed significantly reduced mitochondrial protein levels (TOM20/TIM23) in WT-ISAT mice versus control (*P* < 0.01), consistent with mitophagy activation. This reduction was attenuated in *Pink1*^*−/−*^ and *Park2*^*−/−*^ ISAT groups (*P* < 0.05 vs. WT-ISAT), confirming suppression of mitophagy upon genetic ablation (Fig. 3a, b).

To directly visualize mitochondrial damage and autophagic clearance, we performed transmission electron microscopy (TEM). As shown in Fig. 3c, *Pink1*^−/−^ and *Park2*^−/−^ ISAT mice exhibited numerous damaged mitochondria with disrupted cristae and swelling, whereas such damage was much less frequent in WT-ISAT mice. Quantification of damaged mitochondria per cell confirmed a significant increase in the knockout ISAT mice, which was markedly reduced in WT-ISAT mice (Fig. 3d). Importantly, we also quantified autophagic vacuoles containing damaged mitochondria as a direct measure of mitophagic flux. WT-ISAT mice showed a significant increase in the number of such autophagic vacuoles compared to control mice, whereas this increase was greatly attenuated in *Pink1*^*−/−*^ and *Park2*^*−/−*^ ISAT mice (Fig. 3e). These ultrastructural findings provide direct evidence that PINK1/Parkin-dependent mitophagy is activated in WT-ISAT mice and impaired upon genetic ablation of *Pink1* or *Park2*.

Consistently, H&E staining showed increased lymphocytic infiltration in knockout ISAT mice versus WT controls (Fig. 3f, g). ELISA revealed elevated serum autoantibodies (TgAb: *P* < 0.001; TPO-Ab: *P* < 0.001) in *Pink1*^*−/−*^ and *Park2*^*−/−*^ ISAT groups compared to WT-ISAT (Fig. 3h, i). These findings establish that defective PINK1/Parkin-mediated mitophagy exacerbates autoimmune thyroiditis by impairing mitochondrial quality control.

### The ATM-CHK2 pathway drives mitophagy in response to mitochondrial dysfunction by phosphorylating TAX1BP1 at Ser722

**Fig. 4:**
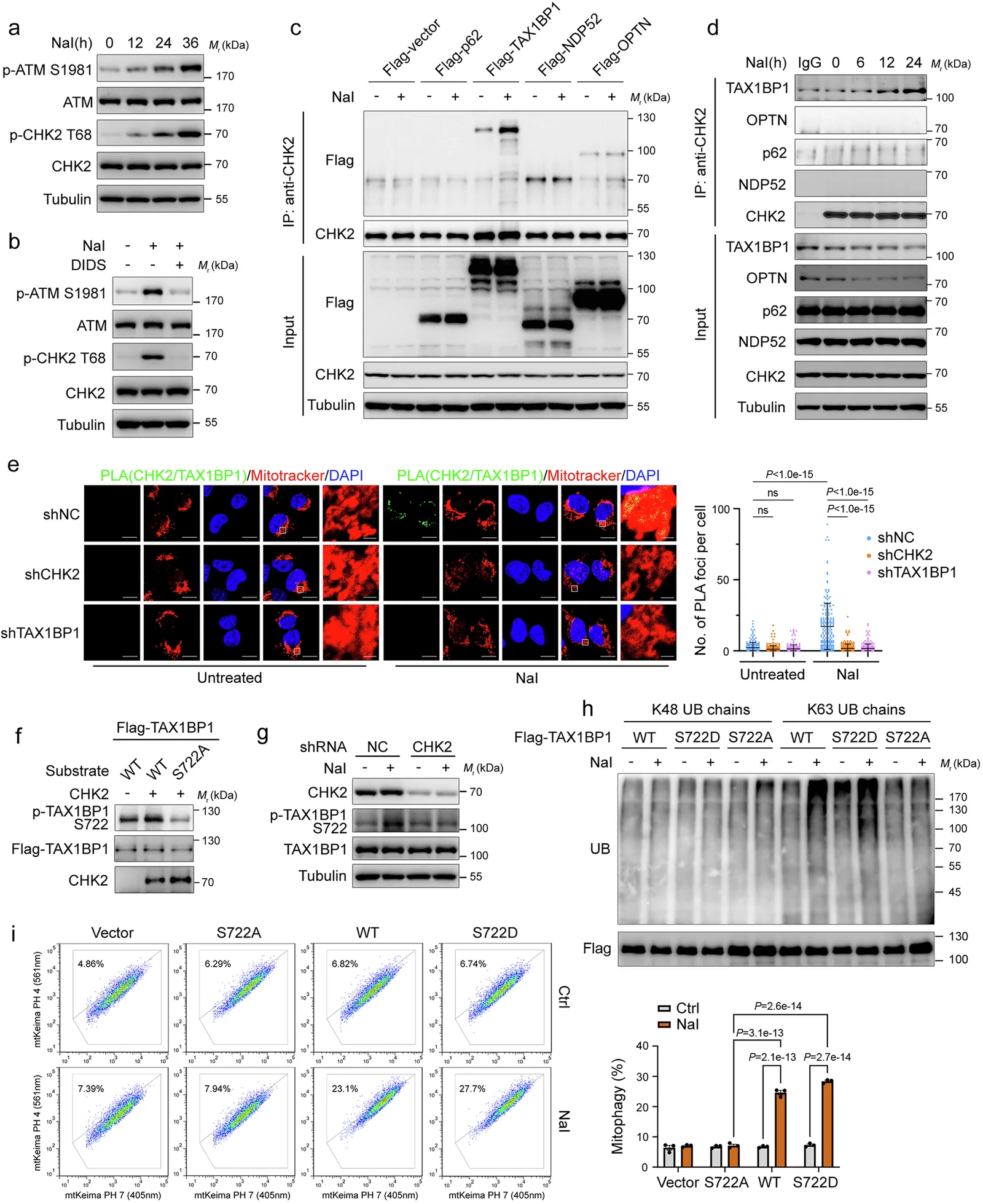
The ATM-CHK2 pathway drives mitophagy in response to mitochondrial dysfunction by phosphorylating TAX1BP1 at ser722. **a** Western blot of p-ATM S1981, ATM, p-CHK2 T68, and CHK2 in Nthy-ori3-1 cells cultured with or without NaI (40 mM). Tubulin was used as a loading control. **b** Western blot of p-ATM Ser1981, ATM, p-CHK2 Thr68, and CHK2 in Nthy-ori3-1 cells following NaI treatment with or without DIDS. **c** Cells were transfected with the indicated plasmid, treated with or without NaI for 24 h and collected for IP and western blot analyses. IP was performed with anti-CHK2, followed by immunoblotting with anti-FLAG or anti-CHK2. **d** Cells were treated with NaI for 0, 6, 12, or 24 h. IP with anti-CHK2 followed by immunoblotting for TAX1BP1, OPTN, p62, NDP52, and CHK2. **e** Evaluation of the in-situ interaction between CHK2 and TAX1BP1 by proximity ligation assay (PLA) in Nthy-ori3-1 cells. Representative images show MitoTracker staining and PLA signals (red) in cells treated with NaI for 24 h. Scale bar: 20 µm (main image), 2 µm (magnified view). **f** In vitro phosphorylation was assessed by incubating purified CHK2 kinase with purified FLAG-TAX1BP1 WT or FLAG-TAX1BP1 S722A in the presence of ATP-γ-S, followed by immunoblotting with the indicated antibodies. **g** Western blot of CHK2, p-TAX1BP1 Ser722, and TAX1BP1 in Nthy-ori3-1 cells transfected with the indicated shRNA, cultured with or without NAI (40 mM) for 24 h. **h** Cells transfected with Flag-TAX1BP1 (WT, S722A, S722D) were cultured with or without NaI (40 mM) for 24 h. Association with K48- and K63-linked ubiquitin chains was analyzed by immunoblotting. **i** Representative FACS plot (left) and quantification (right) of mt-Keima-expressing cells stably transfected with the indicated plasmid, following NaI (40 mM) treatment. Data are representative of at least three independent experiments (**a**, **b**, **g**) or two independent experiments (**c**, **d**, **f**, **h**). For (**e**), *n* = 200 cells per condition from three independent experiments; for (**i**), data are from three independent experiments. Statistics: two‑way ANOVA with Tukey’s multiple comparisons test (two-sided); adjusted *P*-values are shown (**e**, **i**). Data are presented as mean±SEM.

Our search to discover the mechanistic link between iodine-induced mitochondrial dysfunction and mitophagy led us to screen for key regulatory pathways that are affected. Notably, we observed that excess iodine activated the DNA damage response ATM-CHK2 pathway (Fig. 4a). Pharmacological blockade of mitochondrial-cytosolic ROS transport with DIDS completely abrogated this activation (Fig. 4b). To test whether the ATM-CHK2 pathway is required for mitophagy, we knocked down ATM or CHK2 in Nthy-ori3-1 cells and used the mitochondria-targeted Keima fluorescent probe to quantify mitophagic flux. ATM or CHK2 silencing reduced the Keima emission shift from 458 nm to 561 nm, indicating impaired delivery of mitochondria to lysosomal acidic compartments upon NaI treatment (Supplementary Fig. 8a). These findings support a model in which the ATM-CHK2 pathway mediates mitophagy induction under conditions of mitochondrial dysfunction.

We then examined whether CHK2 regulates mitophagy through direct interactions with selective autophagy adaptors, including p62, TAX1BP1, NDP52, and OPTN. Co-immunoprecipitation assays showed that, upon NaI treatment in Nthy-ori3-1 cells, CHK2 specifically interacted with both exogenous and endogenous TAX1BP1, with no detectable binding to the other adaptors (Fig. 4c, d). A proximity ligation assay (PLA) further confirmed increased CHK2-TAX1BP1 interactions within mitochondria under NaI treatment (Fig. 4e). Notably, CHK2 phosphorylation at Thr68 was observed under conditions that promote CHK2-TAX1BP1 association, suggesting that CHK2 activation facilitates this interaction. Consistent with this, a pharmacological CHK2 inhibitor markedly reduced Thr68 phosphorylation and diminished the NaI-induced CHK2-TAX1BP1 interaction (Supplementary Fig. 8b). To map the binding regions of TAX1BP1 responsible for the CHK2 interaction, we tested full-length (FL) and truncated versions of TAX1BP1. The SKICH domain (residues 1-150) of TAX1BP1 was sufficient to bind CHK2 (Supplementary Fig. 8c).

Next, using in vitro kinase assays, we assessed whether CHK2 phosphorylates TAX1BP1. Liquid chromatography-tandem mass spectrometry (LC-MS/MS) identified phosphorylation at Ser722, an evolutionarily conserved residue in TAX1BP1 (Supplementary Fig. 8d, e). For further validation, we generated a phospho-specific antibody recognizing TAX1BP1 phosphorylated at Ser722. In vitro kinase assays confirmed that mutation of Ser722 to alanine (S722A) abolished CHK2-dependent phosphorylation (Fig. 4f). Consistently, endogenous TAX1BP1 Ser722 phosphorylation increased upon NaI treatment in control cells but was abrogated in CHK2-depleted cells (Fig. 4g). These results confirm that CHK2 is the direct upstream kinase that phosphorylates TAX1BP1 at Ser722.

Because TAX1BP1 engages ubiquitinated mitochondria through its ubiquitin-binding zinc finger (UBZ) domain, we asked whether phosphorylation at Ser722, adjacent to the UBZ, modulates ubiquitin-chain recognition. Under NaI stress, co-immunoprecipitation assays revealed that wild-type TAX1BP1 (WT) specifically bound K63-linked ubiquitin chains, whereas the phospho-deficient S722A mutant failed to interact (Fig. 4h). Strikingly, the phospho-mimetic S722D mutant constitutively enhanced K63-ubiquitin chain binding even in the absence of stress.

We next designed rescue assays to determine if CHK2-dependent phosphorylation of TAX1BP1 at Ser722 is required to drive mitophagy. Rescue experiments in TAX1BP1-depleted Nthy-ori3-1 cells reconstituted with wild-type (WT), phospho-deficient (S722A), or phospho-mimetic (S722D) TAX1BP1 (Supplementary Fig. 8f) revealed that NaI-treated cells expressing WT or S722D TAX1BP1 showed a significant reduction of TOM20 and TIM23 compared with S722A TAX1BP1-expressing cells (Supplementary Fig. 8g–i). Consistently, mitophagic flux quantified by mt-Keima emission shift from 458 nm to 561 nm, together with TEM, showed increased mitochondria-engulfing autophagosomes in WT/S722D versus S722A reconstituted cells (Fig. 4i, Supplementary Fig. 8j, k). Together, these data show that CHK2 promotes mitophagy through phosphorylation-dependent activation of TAX1BP1 at Ser722 during excessive iodine-induced mitochondrial stress.

### Mitophagy suppresses mtDNA release-induced cGAS-STING activation

**Fig. 5:**
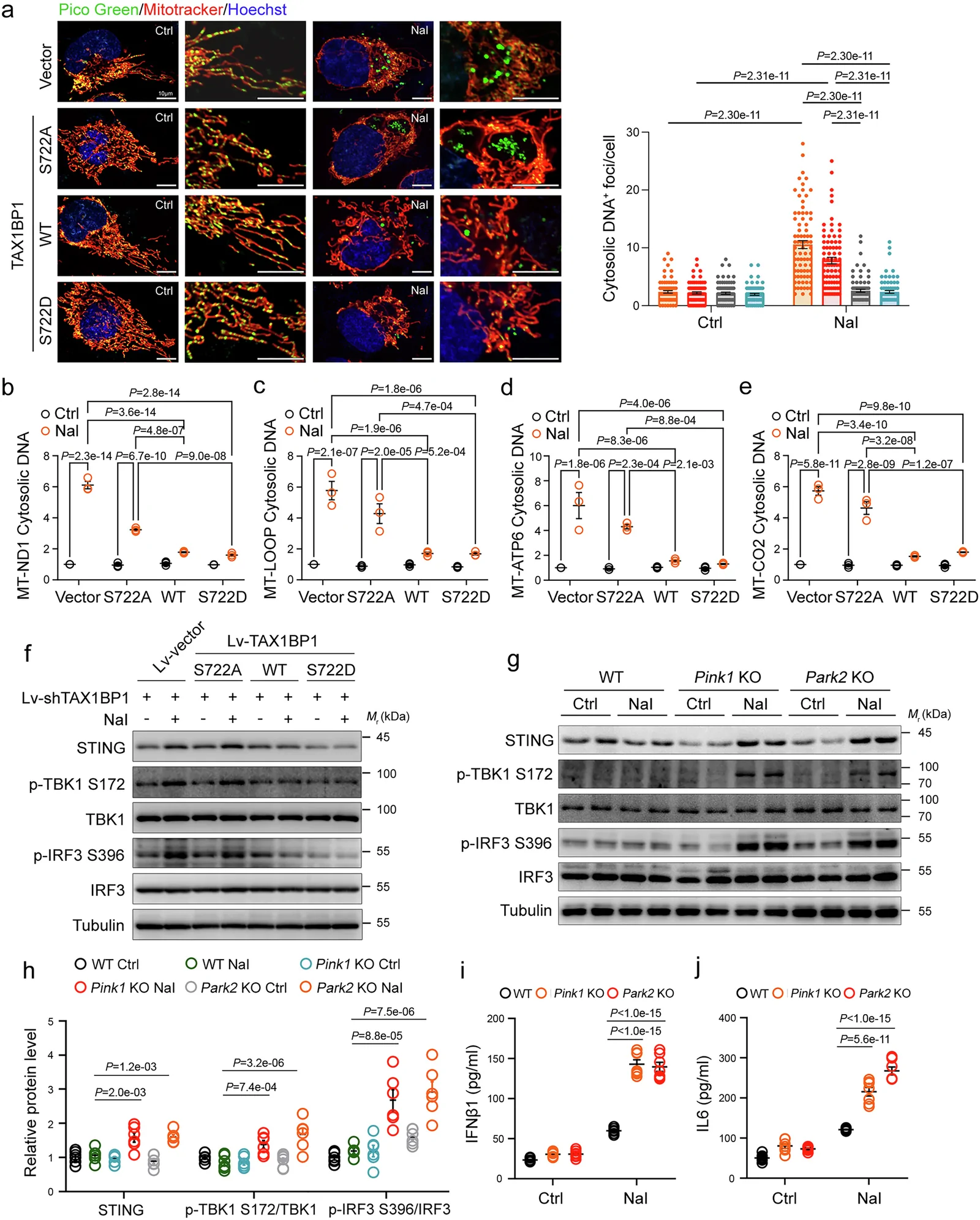
Mitophagy suppresses mtDNA release-induced cGAS-STING activation. **a** Representative confocal images (left) and quantification (right) of Nthy-ori3-1 cells transfected with the indicated shRNA, treated with or without NaI, and stained with PicoGreen (DNA) and MitoTracker (mitochondria). Scale bars: 10 μm (main image and magnified view). Data are presented as mean ± SEM; *n* = 80 cells per group from three independent experiments. Data are presented as fold change relative to the control group (mean of control = 1). Multiple comparisons were performed using two-way ANOVA followed by Tukey’s multiple comparisons test (two-sided). Adjusted *P*-values are shown. **b**–**e** Cytosolic leakage of mtDNA was assessed by qPCR in Nthy-ori3-1 cells cultured in normal medium or treated with NaI (40 mM, 24 h). Data are presented as mean ± SEM from three independent experiments. Data are presented as fold change relative to the control group (mean of control = 1). Multiple comparisons were performed using two-way ANOVA followed by Tukey’s multiple comparisons test (two-sided). Adjusted *P*-values are shown. **f** Nthy-ori3-1 cells were transfected with the Flag-TAX1BP1 WT, Flag-TAX1BP1 S722A and Flag-TAX1BP1 S722D proteins and cultured in normal medium or treated with NaI (40 mM) for 24 h. Western blot analysis of STING, p-TBK1 S172, TBK1, p-IRF3 S396, and IRF3 protein levels was performed. Tubulin was used as a loading control. Three independent experiments were performed. **g**, **h** Relative expression of STING, p-TBK1 (Ser172), TBK1, p-IRF3 (Ser396), and IRF3 in thyroid tissue was assessed by western blot in WT, *Pink1* KO, and *Park2* KO ISAT mice and controls. Tubulin served as the loading control. Data are presented as mean ± SEM (*n* = 8 in each group); Comparisons were performed using two-way ANOVA followed by Tukey’s multiple comparisons test (two-sided). Adjusted *P*-values are shown. **i**, **j** IFNβ1 and IL-6 levels were determined by ELISA. Data are presented as mean ± SEM (*n* = 7 in each group); Multiple comparisons were performed using two-way ANOVA followed by Tukey’s multiple comparisons test (two-sided). Adjusted *P*-values are shown.

Proteomic profiling of the ISAT model revealed concomitant mitochondrial dysfunction and upregulated STING expression in thyroid tissue, along with increased release of mtDNA into the cytosol, suggesting inflammation driven by the mtDNA-cGAS-STING axis may critically contribute to autoimmune thyroiditis progression (Supplementary Fig. 7). To determine whether mitophagy regulates mtDNA release-mediated cGAS/STING activation, we first examined whether the CHK2-TAX1BP1 mitophagy axis modulates mitochondrial stress-induced mtDNA leakage. Confocal microscopy quantification of cytosolic dsDNA foci (PicoGreen) and mitochondrial mass (MitoTracker) showed significant accumulation of extramitochondrial DNA in cells expressing the phospho-deficient TAX1BP1 S722A mutant compared with WT or phospho-mimetic S722D under excess iodine treatment (Fig. 5a). qPCR-based measurement of cytoplasmic mtDNA confirmed these findings (Fig. 5b–e). In parallel, depletion of PINK1 or Parkin similarly increased cytosolic dsDNA foci and cytoplasmic mtDNA (Supplementary Fig. 9a–e). We next investigated the molecular mechanism by which mtDNA is released into the cytosol under conditions of impaired mitophagy. mtDNA can be released through several distinct channels, including BAX/BAK macropores, oligomerized VDAC, and the mitochondrial permeability transition pore (mPTP). To determine which of these pathways is responsible in our model, we treated Nthy-ori3-1 cells with NaI in the presence of a BAX inhibitor (BAI1, 5 μM), a VDAC inhibitor (VBIT-4, 5 μM), or the mPTP inhibitor (cyclosporin A, CsA, 2.5 μM). Cytosolic fractions were isolated, and mtDNA levels were quantified by qPCR. As shown in Supplementary Fig. 9f–q, BAI1 significantly reduced the amount of mtDNA in the cytosol, whereas VBIT-4 and CsA had no effect. To further validate the involvement of BAX/BAK, we knocked down BAX and BAK using specific shRNAs. Silencing of BAX and BAK substantially attenuated NaI induced mtDNA leakage (Supplementary Fig. 9r–v). These results demonstrate that mtDNA release in our model occurs primarily through BAX/BAK dependent pores, rather than through VDAC channels or the mPTP. Together, these findings establish that compromised mitophagy directly potentiates mtDNA leakage during mitochondrial stress.

Reconstitution of TAX1BP1-depleted Nthy-ori3-1 cells with the phospho-deficient TAX1BP1 (S722A), but not wild-type or phospho-mimetic (S722D) variants, amplified cGAS-STING signaling, as evidenced by coordinated upregulation of STING, phospho-TBK1, and activated IRF3 under excess iodine stress (Fig. 5f). This phenotype was recapitulated by PINK1/Parkin depletion (Supplementary Fig. 9w, x), confirming conserved regulation across mitophagy pathways. Critically, in the ISAT model, *Pink1*^−/−^ or *Park2*^−/−^ mice exhibited exaggerated thyroidal activation of the STING-TBK1-IRF3 axis (Fig. 5g, h), accompanied by elevated serum IFNβ1 and IL-6 (Fig. 5i, j), confirming that defective mitophagy leads to the pathological mtDNA leakage that fuels cGAS-STING-dependent inflammation.

### STING inhibition attenuates the progression of autoimmune thyroiditis

**Fig. 6:**
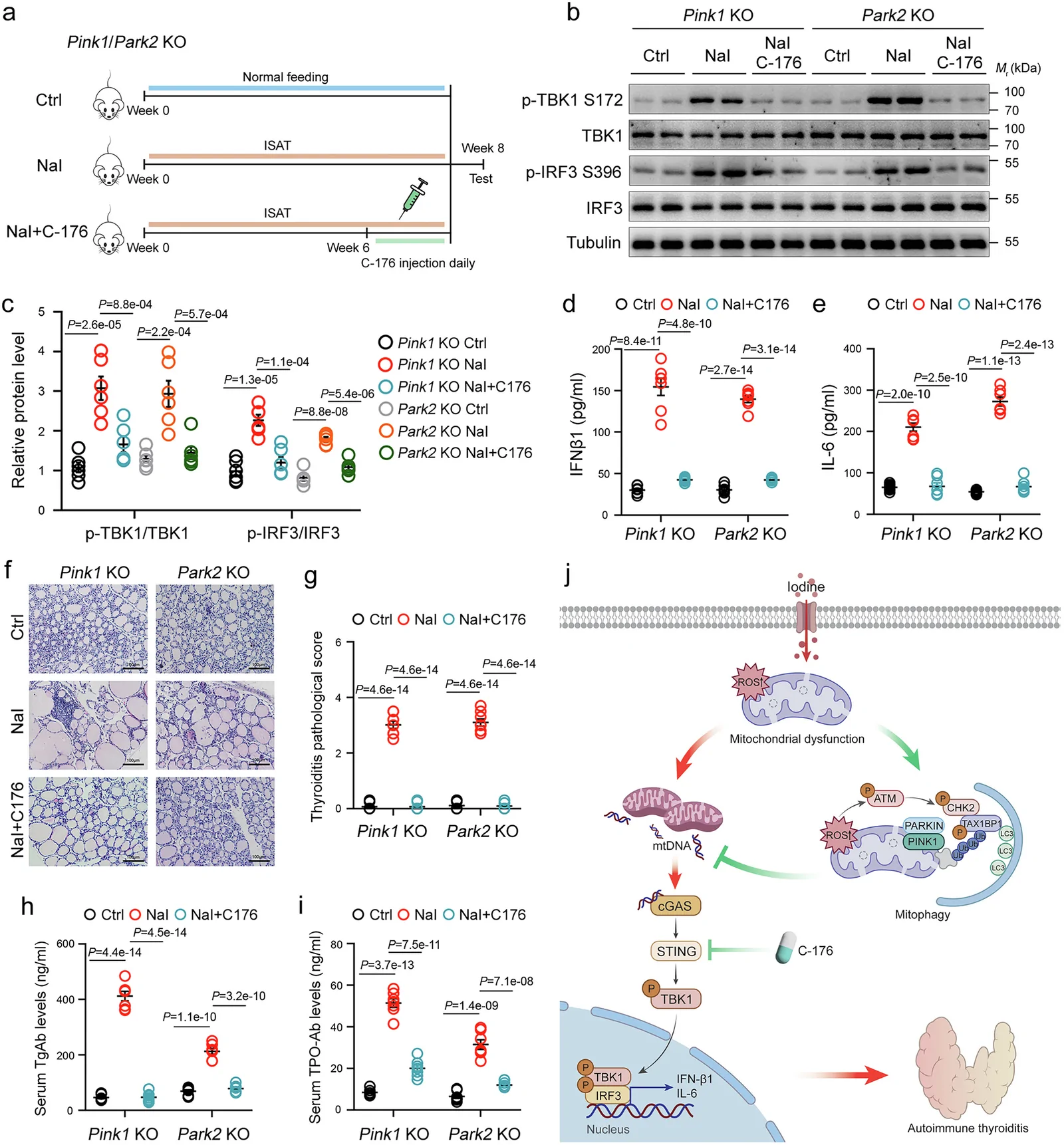
STING inhibition attenuates the progression of autoimmune thyroiditis. **a** Schematic model of STING inhibitor treatment in *Pink1* KO and *Park2* KO ISAT mice. **b**, **c** Western blot analysis of p-TBK1 S172, TBK1, p-IRF3 S396, and IRF3 in thyroid tissue from *Pink1* KO and *Park2* KO ISAT mice treated with or without C-176. Relative protein levels were quantified. Tubulin was used as a loading control. Data are presented as mean ± SEM (*n* = 6 in each group); Comparisons were performed using two-way ANOVA followed by Tukey’s multiple comparisons test (two-sided). Adjusted *P*-values are shown. **d**, **e** IFNβ1 and IL-6 levels were determined by ELISA. Data are presented as mean ± SEM (*n* = 7 in each group); Comparisons were performed using one-way ANOVA followed by Tukey’s multiple comparisons test (two-sided). Adjusted *P*-values are shown. **f**, **g** Thyroid inflammation was assessed on H&E-stained sections by quantifying the area of lymphocytic infiltration. Scale bar, 100 µm. Data are presented as mean ± SEM (*n* = 8 in each group); Comparisons were performed using one-way ANOVA followed by Tukey’s multiple comparisons test (two-sided). Adjusted *P*-values are shown. **h**, **i** Serum TgAb and TPO-Ab levels were determined by ELISA. Data are presented as mean ± SEM (*n* = 7 in each group); Comparisons were performed using one-way ANOVA followed by Tukey’s multiple comparisons test (two-sided). Adjusted *P*-values are shown. **j** Schematic model illustrating how mitophagy mitigates mitochondrial dysfunction-triggered mtDNA-cGAS-STING activation in autoimmune thyroiditis, and how STING inhibition can suppress disease.

To determine the impact of blocking cGAS-STING on autoimmune thyroiditis progression, we first assessed whether the STING inhibitor C-176 suppresses inflammation driven by the mtDNA-cGAS-STING pathway (Fig. 6a). In both *Pink1*^−/−^ and *Park2*^−/−^ ISAT mice, C-176 treatment suppressed phosphorylation of TBK1 and IRF3, which are key downstream effectors of STING (Fig. 6b, c). Notably, C-176 administration also significantly reduced serum IFNβ1 and IL-6 levels in these mice (Fig. 6d, e), indicating effective inhibition of mtDNA-cGAS-STING activation resulting from mitophagy loss.

Given the efficacy in suppressing mtDNA-cGAS-STING activation, we next evaluated the therapeutic effect on autoimmune thyroiditis progression in the ISAT model. As expected, both *Pink1*^−/−^ and *Park2*^−/−^ ISAT mice developed thyroiditis under excess iodine exposure, consistent with cGAS-STING hyperactivation. Notably, a two-week treatment with C-176 significantly attenuated thyroiditis severity (Fig. 6f, g) and reversed elevated serum TgAb and TPOAb levels (Fig. 6h, i), demonstrating that pharmacological STING inhibition effectively blocks disease progression.

## Discussion

Although autoimmune thyroiditis features disrupted thyrocyte homeostasis and immune activation, its pathogenic mechanisms remain incompletely understood. Here, we show that mitochondrial dysfunction promotes cytosolic mtDNA leakage, activating the cGAS-STING pathway and exacerbating autoimmune thyroiditis. Critically, ATM-CHK2-TAX1BP1-mediated activation of PINK1/Parkin-dependent mitophagy suppresses mtDNA-driven cGAS-STING signaling, thereby attenuating disease pathogenesis. Importantly, pharmacological STING inhibition with C-176 ameliorates disease severity, identifying the STING signaling pathway as a promising therapeutic target (Fig. 6j).

A growing body of evidence implicates the cGAS-STING pathway, activated by released mitochondrial DNA (mtDNA), as a key link between mitochondrial damage, sterile inflammation, and chronic immune activation in autoimmune diseases such as Systemic Lupus Erythematosus (SLE)^6–8^ and Sjögren’s syndrome (SS)^31^. Our findings reveal a key pathogenic pathway in autoimmune thyroiditis rooted in the unique biology of thyroid follicular cells, whose hormone synthesis inherently creates a pro-oxidant environment. Thyroid hormone synthesis requires hydrogen peroxide as a crucial cofactor for thyroperoxidase, which oxidizes iodide and facilitates its incorporation into thyroglobulin. This physiologic requirement, however, creates a state of persistent oxidative stress. We show that this stress leads to mitochondrial damage and dysfunction, which in turn activates the cGAS-STING pathway, likely through the release of mitochondrial DNA. Therapeutically, pharmacologic STING inhibition with a small-molecule agent markedly ameliorated disease in our animal model, supporting the potential of targeting this pathway in autoimmune thyroiditis. In the context of autoimmune thyroiditis, elevated STING expression in Hashimoto’s thyroiditis tissues correlates with inflammatory markers, and cGAS-STING activation promotes thyrocyte pyroptosis^9,10^. Our findings extend these observations by identifying the upstream trigger, mitochondrial dysfunction and mtDNA leakage, and by demonstrating that pharmacological STING inhibition with C-176 effectively ameliorates disease in a mouse model.

Mitophagy protects against senescence and inflammatory tissue damage by clearing damaged mitochondria and preventing the activation of the mtDNA-cGAS-STING axis^32–36^. Our data define a cytoprotective mechanism in which PINK1-Parkin-mediated mitophagy acts as a negative regulator of inflammatory signaling in autoimmune thyroiditis. This model is strongly supported by evidence that Pink1 deficiency in mice leads to spontaneous Hashimoto’s thyroiditis with mitochondrial abnormalities, mitophagy defects, and upregulation of STING and NLRP3^25^. While this prior work established the protective role of PINK1, our study further uncovers the upstream regulatory mechanism, the ATM-CHK2-TAX1BP1 axis, that controls PINK1/Parkin-dependent mitophagy in response to mitochondrial stress. By selectively removing impaired mitochondria, this pathway reduces the cytosolic availability of the potent immunostimulant mtDNA, thereby limiting cGAS sensing and downstream STING-dependent type I interferon production. This model supports the concept that enhancing mitophagy may be a helpful therapeutic strategy to disrupt the feed-forward cycle of inflammation and tissue damage.

Our findings reveal an activating regulatory layer for the autophagy receptor TAX1BP1 that complements its known inhibition by TNIP1^23^. We find that mitochondrial stress co-opts the ATM-CHK2 pathway to directly phosphorylate TAX1BP1 at Ser722, adjacent to its ubiquitin-binding zinc finger (UBZ) domain. This phosphorylation functions as a molecular switch that increases TAX1BP1’s affinity for K63-linked ubiquitin chains and is functionally essential for mitophagy, as evidenced by rescue of mitophagic flux only by the phosphomimetic (S722D) mutant.

This convergence of inhibitory (TNIP1) and activating (CHK2) signals on TAX1BP1 underscores its role as a critical gatekeeper. We propose a model where the balance between these opposing forces determines TAX1BP1’s functional state. CHK2-mediated phosphorylation may license TAX1BP1 for mitophagy by allosterically enhancing UBZ domain accessibility or displacing TNIP1, thereby ensuring that robust mitophagy is triggered only under sustained stress. More broadly, these findings highlight the concept that autophagy receptor regulation extends beyond recruitment to precise post-translational control that integrates upstream stress signals, providing insight into mitochondrial homeostasis and suggesting potential therapeutic avenues for diseases marked by mitochondrial dysfunction.

The aggregation of mtDNA signals observed in TAX1BP1 S722A mutant cells superficially resembles the nucleoid clustering reported in Drp1-deficient cells, where mitochondrial fission is blocked and nucleoids accumulate within hyperfused mitochondria^37^. However, the underlying mechanisms and functional consequences are fundamentally distinct. In Drp1-deficient cells, clustered nucleoids remain enclosed within the mitochondrial matrix, leading to sequestration of cytochrome c and delayed apoptosis. In contrast, the TAX1BP1 S722A mutation impairs mitophagy, resulting in the release of mtDNA into the cytosol, as evidenced by the loss of co-localization between PicoGreen-stained mtDNA and the mitochondrial marker MitoTracker. This cytosolic mtDNA then activates the cGAS-STING inflammatory axis. Thus, while both conditions produce aggregated mtDNA signals, they reflect two distinct facets of mitochondrial quality control, one involving fission and cristae architecture to regulate apoptosis, and the other involving autophagic clearance to drive innate immune signaling and inflammation. The morphological similarity suggests that both severe mitochondrial hyperfusion and defective mitophagy can lead to mtDNA aggregation, but the critical difference lies in whether mtDNA is retained within or released from mitochondria.

While our study establishes a protective role for PINK1/Parkin-mediated mitophagy in autoimmune thyroiditis, we acknowledge a limitation in our experimental approach. The use of systemic *Pink1*^−/−^ and *Park2*^−/−^ mouse models precludes definitive exclusion of cell type-specific contributions, particularly from immune cells where mitophagy also modulates inflammatory responses. Nonetheless, multiple lines of evidence indicate that the exacerbated thyroiditis we observe is primarily driven by defective mitophagy within thyrofollicular epithelial cells. First, immunohistochemical analyses of human HT specimens showed localized upregulation of PINK1, Parkin, and activated STING signaling was limited to thyrofollicular epithelial cells and not infiltrating lymphocytes. Second, in vitro studies using Nthy-ori3-1 thyrocytes recapitulated the key findings, showing that mitophagy impairment directly causes mtDNA leakage and cGAS-STING activation in this relevant cell type. Although future studies using thyrocyte-specific conditional knockouts will be valuable for dissecting cell-autonomous mechanisms, the concordance between our in vivo and in vitro data provides evidence that epithelial mitophagy is pivotal in restraining thyroiditis pathogenesis.

Collectively, our work unveils a pathogenic axis in AIT in which thyrocyte mitochondrial damage activates cGAS-STING via mtDNA release and shows that ATM-CHK2-driven mitophagy counterbalances this inflammatory cascade. This reframes AIT as a disorder in which mitochondria play an important role and highlights mitophagy enhancement as a possible therapeutic avenue.

## Methods

### Reagents and antibodies

Antibodies against PINK1 (#6946), Parkin (#4211), TOM20 (#42406), Phospho-ATM Ser1981 (#13050), ATM (#2873), Phospho-CHK2 Thr68 (#2661), CHK2 (#3440), STING (#13647), p-TBK1 Ser172 (#5483), TBK1 (#3504), p-IRF3 Ser396 (#29047), IRF3 (#4302), NIX (#12396), TAX1BP1 (#5105), NDP52 (#60732), Ubiquitin (#3933), and α-Tubulin (#2144) were purchased from Cell Signaling Technology. Antibody against OPTN (10837-1-AP), BAX (50599-2-lg), and BAK (29552-1-AP) were purchased from Proteintech Technology. Antibodies against TIM23 (NBP2-13432) and FUNDC1 (NBP1-56430) were purchased from NOVUS Biologicals. Antibody against p62 (P0067) was purchased from Sigma. An additional antibody against Parkin (sc-32282) was purchased from Santa Cruz Biotechnology. Anti-Flag tag antibody (SG4110-16) was purchased from Shanghai Genomics Technology. A rabbit antiserum against TAX1BP1 phosphorylated at Ser722 was raised against peptide FDS(P)SFDVHK-C, of which the serine is phosphorylated and indicated as S (P). The antiserum was precleaned using the corresponding nonphosphorylated peptide coupled to SulfoLink Coupling Resin (Thermo Scientific) and purified by affinity chromatography. CHK2 inhibitor II was from Sigma (St. Louis, MO). BAX inhibitor (BAI1), VDAC inhibitor (VBIT-4), mPTP inhibitor (Cyclosporin A), DIDS, C-176, and corn oil were from MedChemExpress. Protein A/G agarose was from Santa Cruz.

### DNA construction, mutagenesis and viral infection

Flag-TAX1BP1, Flag-OPTN, Flag-NDP52, and Flag-p62 were purchased from GeneChem Company. Flag-TAX1BP1 ΔUBZ (amino acids 1–725), Flag-TAX1BP1 CC (amino acids 150–600), and Flag-TAX1BP1 ΔSKICH (amino acids 127–789) were obtained from MiaoLingBio. Mutagenesis (TAX1BP1 S722A and TAX1BP1 S722D) was performed via site-directed mutagenesis. The TAX1BP1 shRNAi-resistant (nontargetable) mutant plasmids were generated by mutating three nucleotide base pairs to alternative nucleotides within the shRNA target region without altering the amino acid sequences. The sequences of all constructs were confirmed by DNA sequencing. For lentiviral production and infection, shRNA against ATM (shATM), CHK2 (shCHK2), TAX1BP1 (shTAX1BP1), BAX (shBAX), and BAK (shBAK) lentivirus were purchased from Shanghai GeneChem Company. PINK1 siRNA and Parkin siRNA were purchased from RiboBio Co., Ltd., Guangzhou, China.

### Cell lines and mice

Nthy-ori3-1 cells were obtained from the European Collection of Authenticated Cell Cultures (ECACC). The cell line was derived from a 35-year-old female donor and immortalized by transfection with a plasmid encoding the SV40 large T antigen. Cells were cultured in RPMI-1640 supplemented with 10% fetal bovine serum, 100 U/mL penicillin, and 100 µg/mL streptomycin.

NOD.H-2^h4^ mice were obtained from The Jackson Laboratory (Bar Harbor, ME, USA; stock number 004447). *Pink1*-deficient (C57BL/6JCya-Pink1^em1^/Cya; product ID S-KO-12891) and *Park2*-deficient (C57BL/6JCya-Prkn^em1^/Cya; product ID S-KO-10234) mice were purchased from Cyagen Biosciences (Santa Clara, CA, USA). These knockout mice were generated by Cyagen using CRISPR/Cas9-mediated gene editing on a C57BL/6 N background. To obtain congenic strains on the NOD.H-2^h4^ background, each knockout line was backcrossed to NOD.H-2^h4^ mice for more than 10 generations (*N* > 10). Genotyping was performed every generation using PCR to confirm the retention of the knockout allele and the NOD.H-2^h4^ genetic background. All animals were maintained under SPF conditions with a 12 h light/dark cycle, at a controlled temperature of 22–24 °C and relative humidity of 45–65%, and with free access to food and water. Wild-type, *Pink1* knockout (*Pink1*^−/−^), and *Park2* knockout (*Park2*^−/−^) NOD.H-2^h4^ mice (4–5 weeks of age) were randomly divided into control groups and high-iodine (0.05% sodium iodine) groups. Mice were fed with high-iodine water for 8 weeks. For efficacy studies in *Pink1*^−/−^ and *Park2*^−/−^ mice, animals receiving high-iodine water were injected daily with 562.5 nmol C-176 or vehicle (DMSO) in 200 µL for 2 weeks. Mice were euthanized by isoflurane inhalation followed by cervical dislocation. All animal experiments were approved by the Institutional Animal Care and Use Committee (IACUC) of China Medical University and were performed in accordance with applicable regulations.

### Human thyroid samples

Human thyroid specimens were obtained from the First Affiliated Hospital of China Medical University. Tissues were collected between 2019 and 2023 from adult patients who underwent thyroidectomy for thyroid nodules. A total of 63 adult patients with Hashimoto’s thyroiditis and 40 adult healthy controls were included based on clinical and histopathological diagnosis. Written informed consent was obtained from all participants. The study protocol was approved by the Institutional Research Ethics Committee of the First Affiliated Hospital of China Medical University.

### Enzyme-linked immunosorbent assay (ELISA)

Levels of mouse TgAb, TPO-Ab, IFNβ1, and IL-6 are determined by enzyme-linked immunosorbent assay (Shanghai enzyme-linked biotechnology) according to the manufacturer’s instructions.

### Assessment of autoimmune thyroiditis

Thyroid lobes were dissected under anesthesia, fixed in 4% paraformaldehyde for at least 24 h, embedded in paraffin, and sectioned at 3 µm. Sections were stained with hematoxylin and eosin (H&E), and histological changes were examined by light microscopy. Lymphocytic infiltration was graded as previously described^38^. Briefly, HE-stained thyroid sections were graded on the following scale according to the approximate area of lymphocytic infiltration (0, 0%; 1, 1–10%; 2, 11–30%; 3, 31–50%; and 4, > 50%). The thyroiditis scores were expressed as means of at least three noncontiguous sections from each thyroid gland.

### Immunohistochemical (IHC) staining

Paraffin-embedded human Hashimoto’s thyroiditis and normal thyroid sections were stained with antibodies against STING, PINK1, Parkin, p-IRF3 Ser396, and p-TBK1 Ser172 with nonspecific IgG as a negative control. The IHC scores were assessed by two independent authors blinded to the patients’ clinicopathological data. Tissue sections were quantitatively scored according to the staining intensity (0, no signal; 1, weak; 2, moderate; and 3, strong) and percentage of positive cells (1, 0–25%; 2, 26–50%; 3, 51–75%; and 4, > 75%). The final score was calculated by multiplying the intensity score by the proportion score, yielding a total score ranging from 0–12.

### Detection of mtDNA in cytosolic extracts

Digitonin extracts from Nthy-ori3-1 cells were adapted from West et al^39^. as follows: siNC, siPINK1, and siParkin Nthy-ori3-1 cells exposed to 40 mM NaI for 72 h (1 × 10^7^) were each divided into two equal aliquots, and one aliquot was resuspended in 500 µl of 50 µM NaOH and boiled for 30 min to solubilize DNA. 50 µl of 1 M Tris-HCl (pH 8.0) was added to neutralize the pH of the lysate, and the extracts served as normalization controls for total mtDNA. The second equal aliquot was resuspended in roughly 500 µl buffer containing 150 mM NaCl, 50 mM HEPES pH7.4, and 20 μg/ml digitonin (MedChemExpress). The homogenates were incubated end over end for 10 min to allow selective plasma membrane permeabilization, then centrifuged at 980 g for 3 min three times to pellet intact cells. The cytosolic supernatants were transferred to fresh tubes and spun at 17000 g for 10 min to pellet any remaining cellular debris, yielding cytosolic preparations free of nuclear, mitochondrial and endoplasmic reticulum contamination. DNA was then isolated from both the cytosolic fractions and the whole-cell normalization controls using QIAQuick Nucleotide Removal Columns (QIAGEN) according to the manufacturer’s protocol.

Quantitative PCR was performed using TB Green® Premix Ex Taq™ II (Tli RNaseH Plus, TaKaRa, Cat. No. RR820A) on a QuantStudio 6 Flex Real-Time PCR System (Roche). Each reaction was run in triplicate in a 20 µL volume containing 10 µL of 2 × TB Green Premix, 0.4 µL each of forward and reverse primers (10 µM), 2 µL of template DNA, and 7.2 µL of RNase-free water. The thermal cycling conditions were: 93 °C for 1 min, followed by 45 cycles of 55 °C for 1 min and 72 °C for 1 min. A dissociation curve analysis was performed after each run to confirm amplification specificity.

The following primer pairs were used. For human targets: *ND1* (forward 5′-CACCCAAGAACAGGGTTTGT-3′, reverse 5′-TGGCCATGGGTATGTTGTTAA-3′); *LOOP* (forward 5′-CTATCACCCTATTAACCACTCA-3′, reverse 5′-TTCGCCTGTAATATTGAACGTA-3′); *ATP6* (forward 5′-AATCCAAGCCTACGTTTTCACA-3′, reverse 5′-AGTATGAGGAGCGTTATGGAGT-3′); *CO2* (forward 5′-AATCGAGTAGTACTCCCGATTG-3′, reverse 5′-TTCTAGGACGATGGGCATGAAA-3′). For mouse targets: *Nd1* (forward 5′-TCCGAGCATCTTATCCACGC-3′, reverse 5′-GTATGGTGGTACTCCCGCT-3′); *Loop* (forward 5′-AATCTACCATCCTCCGTGAAACC-3′, reverse 5′-TCAGTTTAGCTACCCCCAAGTTTAA-3′); *Co2* (forward 5′-ATAACCGAGTCGTTCTGCCAAT-3′, reverse 5′-TTTCAGAGCATTGGCCATAGAA-3′). The nuclear-encoded control gene *RPL13A* (human: forward 5′-GCCCTACGACAAGAAAAAGCG-3′, reverse 5′-TACTTCCAGCCAACCTCGTGA-3′; mouse: forward 5′-CTGCTCTCAAGGTTGTTCGGCT-3′, reverse 5′-CCTTCCGTTTCTCCTCCAGAGT-3′) was used to verify the absence of nuclear DNA contamination in the cytosolic fractions; no *RPL13A* signal was detected in any cytosolic sample.

The amount of mtDNA in the cytosolic fraction was normalized to total mtDNA from the whole-cell extract (the normalization control). Relative mtDNA levels were calculated using the ΔΔCt method. For each sample, the Ct value of the target mtDNA region was first normalized to the corresponding total mtDNA Ct value (ΔCt = Ct_target – Ct_total_mtDNA). The ΔΔCt was then calculated as ΔCt_treatment—ΔCt_control, and fold change was determined as 2^–ΔΔCt. Data are presented as fold change relative to the control group (mean of control group set to 1). The absence of nuclear DNA contamination was further confirmed by the lack of *RPL13A* amplification in the cytosolic fractions.

### Western blot and immunoprecipitation

Cells were lysed with IP lysis buffer (50 mM Tris-Cl, pH 7.4, 1% Triton X-100, 1% NP-40, 150 mM NaCl, 1 mM EDTA, 0.25% sodium deoxycholate, and protease inhibitor cocktail), and lysates were centrifuged at 18,300 × *g* for 20 min at 4 °C. The supernatants were collected for Western blot analysis or immunoprecipitation.

For Western blotting, protein samples were separated by SDS-PAGE and transferred onto PVDF membranes (Millipore). Membranes were blocked with 5% non-fat milk in TBST (Tris-buffered saline containing 0.1% Tween-20) for 1 h at room temperature, then incubated with primary antibodies (1:1000 dilution) overnight at 4 °C in blocking buffer. After three washes with TBST (10 min each), membranes were incubated with HRP-conjugated secondary antibodies (1:5000 dilution) for 1 h at room temperature, followed by three additional washes. Immunoreactive bands were visualized using enhanced chemiluminescence (ECL) substrate (Tanon, 180-5001) and detected on a MicroChemi 4.2 imaging system (DNR). Band intensities were quantified using ImageJ (National Institutes of Health).

For immunoprecipitation (IP), primary antibodies were coupled to Protein A/G agarose beads (Santa Cruz Biotechnology) by gentle rotation for 2 h at 4 °C. The antibody-bead complexes were then added to cell lysates and incubated overnight at 4 °C with rotation. Beads were washed three times with IP lysis buffer, and bound proteins were eluted by boiling in 2 × SDS sample buffer for 5 min. Eluates were separated by SDS-PAGE and analyzed by immunoblotting as described above.

### In vitro kinase assay

Kinase assays were performed as described previously^17^. Briefly, purified Flag-TAX1BP1 WT or S722A was incubated with His-CHK2 (PV3367, Thermo Fisher Scientific) in 25 μl kinase buffer (50 mM HEPES [pH 7.4], 10 mM MgCl_2_, 10 mM MnCl_2_, 0.2 mM DTT, 100 μM ATP) at 30 °C for 45 min. The reaction was terminated by adding SDS-PAGE loading buffer and heating to 100 °C for 5 min. The reaction mixture was then subjected to SDS-PAGE.

### Mass spectrometry analysis

For the detection of phosphorylation sites, in vitro phosphorylation of TAX1BP1 by CHK2 was performed according to the in vitro kinase assay protocol described above. Protein samples were digested using trypsin and analyzed using LC-MS/MS. The raw data were processed using Proteome Discoverer (PD, version 2.1), and MS/MS spectra were searched against the reviewed Swiss-Prot human proteome database. All searches were carried out with precursor mass tolerance of 7 p.p.m., fragment mass tolerance of 20 milli mass unit (mmu), phosphorylation (+79.966 Da) as variable modifications, carbamidomethylation (Cys) (+57.0215 Da) as fixed modification and two trypsin missed cleavages allowed. Only peptides with at least six amino acids in length were considered. The peptide and protein identifications were filtered by PD to control the false discovery rate (FDR) < 1%. At least one unique peptide was required for protein identification. Mass spectrometric analysis of a tryptic fragment at m/z 607.57355 Da (−1.24 mmu/−2.04 ppm), which was matched with the +3 charged peptide 713-GHGTGFCFDSSFDVHK-728, suggested that TAX1BP1 S722 was phosphorylated. The XCorr score was 4.37.

### Mito-Keima mitophagy assay

Stable cell lines expressing mt-Keima were generated using a lentiviral system. The mt-Keima lentiviral particles were constructed and packaged by Hanbio Biotechnology (Shanghai, China). Nthy-ori3-1 cells were seeded in 6-well plates at a density of 2 × 10^5^ cells per well and cultured overnight to reach 30–50% confluence. The culture medium was then replaced with fresh medium containing 5 μg/mL polybrene, and the cells were infected with the mt-Keima lentivirus at a multiplicity of infection (MOI) of 20. After 24 h of infection, the virus-containing medium was removed and replaced with fresh complete medium. At 72 h post-infection, stable transductants were selected by adding puromycin at a final concentration of 2 μg/mL for 7 days, with medium changes every 2–3 days. The expression of mt-Keima was confirmed by fluorescence microscopy (excitation at 458 nm and 561 nm). The established stable cell line was maintained in culture medium containing 1 μg/mL puromycin. For mitophagy analysis, cells were seeded in 6-well plates, allowed to recover for 24 h, and then treated with NaI as indicated in the figure legends. After treatment, cells were harvested for flow cytometry (FACS) analysis. Lysosomal mt-Keima was quantified by dual-excitation ratiometric measurements at 405 nm (pH 7) and 561 nm (pH 4) with 610/20 nm emission filters. For each sample, 10,000 events were collected. The data were analyzed using FlowJo.

### Fluorescence microscopy

Nthy-ori3-1 cells expressing mt-Keima were grown in confocal dishes and cultured in the indicated conditions. The live-cell mt-Keima signal was obtained using a 60 × oil lens objective on an inverted fluorescence microscope (Nikon, A1RHD25, Japan, Tokyo). Fluorescence of mt-Keima was imaged in 2 channels via 2 sequential excitations (458 nm, green; 561 nm, red) and using a 609- to 735-nm emission range. Mitophagy events were determined as the number of puncta per cell in images generated by subtracting the signal at a 458 nm excitation (reporting a neutral pH environment) from the signal at a 561 nm excitation (reporting an acidic pH-environment).

For mtDNA staining, Nthy-ori3-1 cells were treated with MitoTracker Red (for mitochondrial staining, Yeasen Biotechnology) for 1 h followed by 15 min of incubation in PicoGreen (for dsDNA staining, Yeasen Biotechnology). Cells were imaged on a Nikon microscopy with a 60 × oil-immersed objective.

PLA was performed using the NaveniFlex Cell MR Atto647N (Navinci) according to the manufacturer’s instructions. Images were captured using a 60 × oil lens objective on an inverted fluorescence microscope (Nikon, A1RHD25, Japan, Tokyo) and quantified by ImageJ. Antibody specificity was validated by knockdown approaches.

### Electron microscopy

Cells grown in 10-cm dishes and mouse thyroid tissue were fixed in 2.5% glutaraldehyde for 24 h at 4 °C. Post-fixation, cells or tissues were washed three times in PBS and then post-fixed with 1% osmium tetroxide in 0.1 M cacodylate buffer for 1 h. After washing in PBS, the samples were dehydrated through a graded series of ethanol and embedded in Poly/bed 812 epoxy resin (Polysciences, 02597-50). Ultrathin (60 nm) sections were collected on copper grids and stained with 2% uranyl acetate in 50% methanol for 10 min, followed by 1% lead citrate for 7 min. Images were acquired with a JEOL JEM1210 transmission electron microscope (JEOL). Damaged mitochondria were identified based on distinct morphological features, including disruption of cristae structure and reduced cristae density.

### Proteomics

#### Protein extraction

Thyroid samples were removed from −80 °C and ground in liquid nitrogen to a fine powder. Four volumes of pre-cooled 10% TCA/acetone were added, and proteins were precipitated at −20 °C for 4 h. Samples were centrifuged at 4500 *g* at 4 °C for 5 min, and the precipitate was washed 3 times with the pre-cooled acetone. Four volumes of lysis buffer (8 M urea, 1% protease inhibitor cocktail) were added to the protein precipitate, followed by sonication for three minutes on ice using a high intensity ultrasonic processor (Scientz). The remaining debris was removed by centrifugation at 12,000 *g* at 4 °C for 10 min. Finally, the supernatant was collected, and the protein concentration was determined with a BCA kit according to the manufacturer’s instructions.

#### Trypsin digestion

Equal amounts of protein were enzymatically hydrolyzed, and the volume was adjusted to be consistent with the lysate. Proteins were precipitated at a final concentration of 20% (w/v) TCA, vortexed, and incubated at 4 °C for 2 h. Precipitates were collected by centrifugation at 4500 *g* for 5 min at 4 °C, washed three times with pre-cooled acetone, and air-dried. Pellets were redissolved in 200 mM TEAB and ultrasonically dispersed. Trypsin was added at 1:50 trypsin-to-protein mass ratio for the first digestion overnight. The sample was reduced with 5 mM dithiothreitol for 30 min at 56 °C and alkylated with 11 mM iodoacetamide for 15 min at room temperature in darkness. Finally, the peptides were desalted by Strata X SPE column.

#### LC-MS/MS (liquid chromatography-tandem mass spectrometry) Analysis

The tryptic peptides were dissolved in solvent A and separated using the Ultra Performance Liquid System (vanquish neo). The mobile phase consisted of solvent A (0.1% formic acid, 2% acetonitrile/in water) and solvent B (0.1% formic acid, 90% acetonitrile/in water). Peptides were separated with the following gradient: 0–14.5 min, 6–22%B; 14.5–17.5 min, 22–34%B; 17.5–19 min, 34–80%B; 19–20 min, 80%B, and all at a constant flow rate of 700 nl/min on an EASY-nLC 1200 UPLC system (ThermoFisher Scientific). The peptides were separated by an ultra-performance liquid chromatography system, injected into an NSI ion source, ionized, and analyzed on an Orbitrap Exploris 480 mass spectrometry. The electrospray voltage applied was 2300 V. FAIMS compensate voltage (CV) was set as −45 V. Precursors and fragments were analyzed at the Orbitrap detector. The full MS scan resolution was set to 60000 for a scan range of 350–1400 m/z. The MS/MS scan was fixed first mass as 120.0 m/z at a resolution of 15000. The data acquisition mode uses a data-independent scanning (DIA) procedure, in which peptide ions from multiple continuous m/z windows are followed into the HCD collision cell after a primary scan and fragmented using 27% of the fragmentation energy, followed by secondary mass spectrometry. In order to improve the effective utilization of the mass spectrometry, the automatic gain control (AGC) was set to 1E6 and the maximum injection time was set to 22 ms.

#### Database Search

The DIA data were processed using DIA-NN search engine (v.1.8). Tandem mass spectra were searched against Mus_musculus_10090_SP_20230103.fasta (17132 entries) and Homo_sapiens_9606_SP_20231220.fasta (20429 entries) concatenated with reverse decoy database. Trypsin/P was specified as cleavage enzyme allowing up to 1 missing cleavage. Excision on N-term Met and carbamidomethyl on Cys were specified as fixed modification. FDR was adjusted to <1%.

#### Bioinformatic analysis

GO and KEGG functional enrichment analyses were performed using InterProScan (*p* < 0.05) and the KOG database (http://www.ncbi.nlm.nih.gov/KOG).

Protein Extraction, Trypsin Digestion, LC-MS/MS analysis, and data processing were conducted by Jingjie PTM BioLab (Hangzhou). Co., lnc.

#### Statistical analysis

One-way analysis of variance (ANOVA) was used for multiple-group comparisons and two-way ANOVA with Tukey’s multiple comparisons test was used when two conditions were considered between the groups. Statistical calculations were carried out using GraphPad Prism 10. Data are presented as mean ± SEM. Data that did not conform to normal distribution were described as median (interquartile spacing), and the Mann-Whitney U rank-sum test was used for between-group comparisons, and the correlation between variables was analyzed using the Spearman correlation test. We tested data for normality and variance and considered a *P*-value of less than 0.05 as significant.

### Reporting summary

Further information on research design is available in the Nature Portfolio Reporting Summary linked to this article.

## Supplementary information

Supplementary Information

Transparent Peer Review file

Reporting Summary

## Source data

Source Data

## Data Availability

Source Data are provided with this paper. The mass spectrometry proteomics data have been deposited to the ProteomeXchange Consortium via the iProX partner repository with the dataset identifier PXD070578. Source data are provided with this paper.
