# Supplementary material for: Mitophagy mitigates mitochondrial DNA-induced activation of cGAS-STING in autoimmune thyroiditis
Source: Nat Commun. 2026 Jul 24;17:9041. doi: 10.1038/s41467-026-76047-9 (PMC13507228; doi:10.1038/s41467-026-76047-9)
Supplement: Supplementary file 1 [file 41467_2026_76047_MOESM1_ESM.pdf]

Supplementary information

Mitophagy mitigates mitochondrial DNA-induced activation of cGAS-STING in autoimmune thyroiditis

Xiao-Chen Xie[1,#,*], Yang Guo[2,3,#], Ran Guo[4,#], Yong-Ze Li[1,#], Shan-Shan Wang[2,3], Xiao-You Jiang[2,3], Shuang Hao[2,3], Ye Zhang[2,3], Yu-Han Li[2,3], Xi-Yan Liu[2,3], Xiao-Xu Wu[2,3], Xin-Yue Zhang[2,3], Wen-Dong Guo[2,3], Yan-Ling Feng[2,3], Jia-Bin Li[5], Chen Liu[5], Liang Wang[6], Zhen-Hua Li[7], Wei-Ping Teng[1,*], Zhong-Yan Shan[1,*], Liu Cao[8,*], Qi-Qiang Guo[2,3,*].

[1]Department of Endocrinology and Metabolism, Institute of Endocrinology, NHC Key Laboratory of Diagnosis and Treatment of Thyroid Diseases, The First Affiliated Hospital of China Medical University, Shenyang, Liaoning Province, China.
[2]The College of Basic Medical Science, Health Sciences Institute, China Medical University, Shenyang, Liaoning Province, China.
[3]Key Laboratory of Cell Biology of Ministry of Public Health, Key Laboratory of Medical Cell Biology of Ministry of Education, Key Laboratory of Precision Diagnosis and Treatment of Gastrointestinal Tumors of Ministry of Education, Liaoning Province Collaborative Innovation Center of Aging Related Disease Diagnosis and Treatment and Prevention, China Medical University, Shenyang, Liaoning Province, China.
[4]Department of Orthopedics, Shengjing Hospital of China Medical University, Shenyang, Liaoning Province, China.
[5]Department of Cell Biology, Key Laboratory of Cell Biology, National Health Commission of the PR China, Key Laboratory of Medical Cell Biology, Ministry of Education of the PR China, China Medical University, Shenyang, Liaoning Province, China.
[6]Department of Pathology, College of Basic Medical Sciences, China Medical University, Shenyang, Liaoning Province, China.
[7]School of Pharmacy, China Medical University, Shenyang, Liaoning Province, China.
[8]Clinical Translational Research Center, Shengjing Hospital of China Medical University, Shenyang, Liaoning Province, China.
[#]These authors contributed equally.
*Correspondence: xcxie@cmu.edu.cn (X.-C.X.); twp@vip.163.com (W.-P.T.); shanzhongyan@medmail.com.cn (Z.-Y.S.); lcao@cmu.edu.cn (L.C.); qqguo@cmu.edu.cn (Q.-Q.G.).

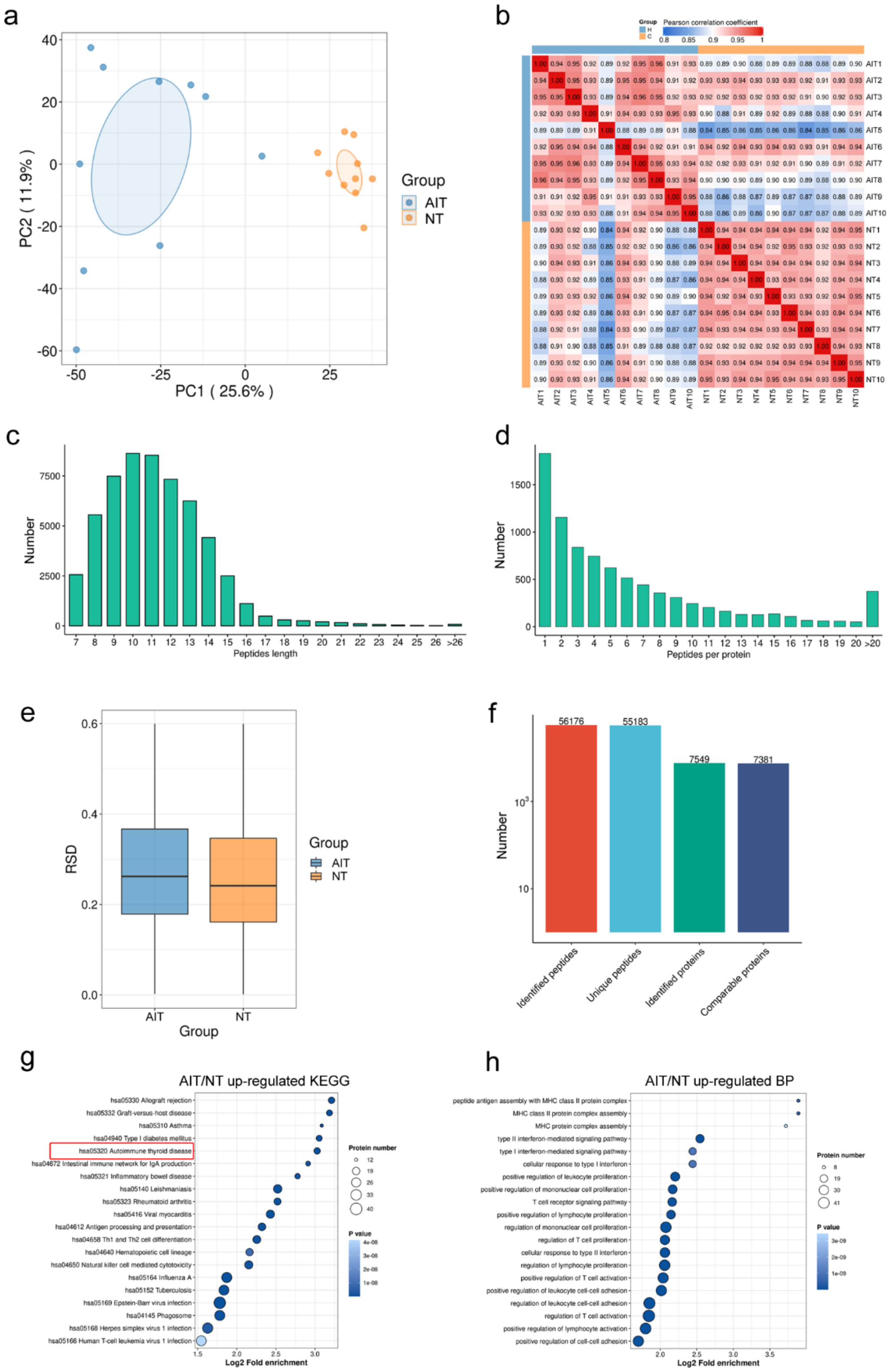

Supplementary Fig. 1 Quality control of the proteomics data from human thyroid tissues of healthy control and autoimmune thyroiditis patients

(a) PCA of relative quantitative data. Axes show the proportion of variance explained. (b) Pairwise Pearson correlation analysis of sample intensities. Replicate samples within each group show high correlation (red). (c) Histogram displaying the distribution of identified peptide lengths. (d) Distribution of the number of peptides identified per protein. (e) Box plot illustrating the distribution

of the relative standard deviation (RSD) for relative quantification values within replicate samples of each group. (f) Bar plot summarizing key metrics of the proteomic analysis: the total number of identified peptides, unique peptides, identified proteins, and quantifiable proteins. (g) KEGG enrichment analysis in thyroid tissue for upregulated proteins comparing autoimmune thyroiditis patients with healthy controls. Bubble color reflects Fisher's exact test P value, and bubble size reflects the number of proteins. (h) GO biological processes (BP) enrichment analysis in thyroid tissue for upregulated proteins comparing autoimmune thyroiditis patients with healthy controls. Bubble color reflects Fisher's exact test *P* value, and bubble size reflects the number of proteins.

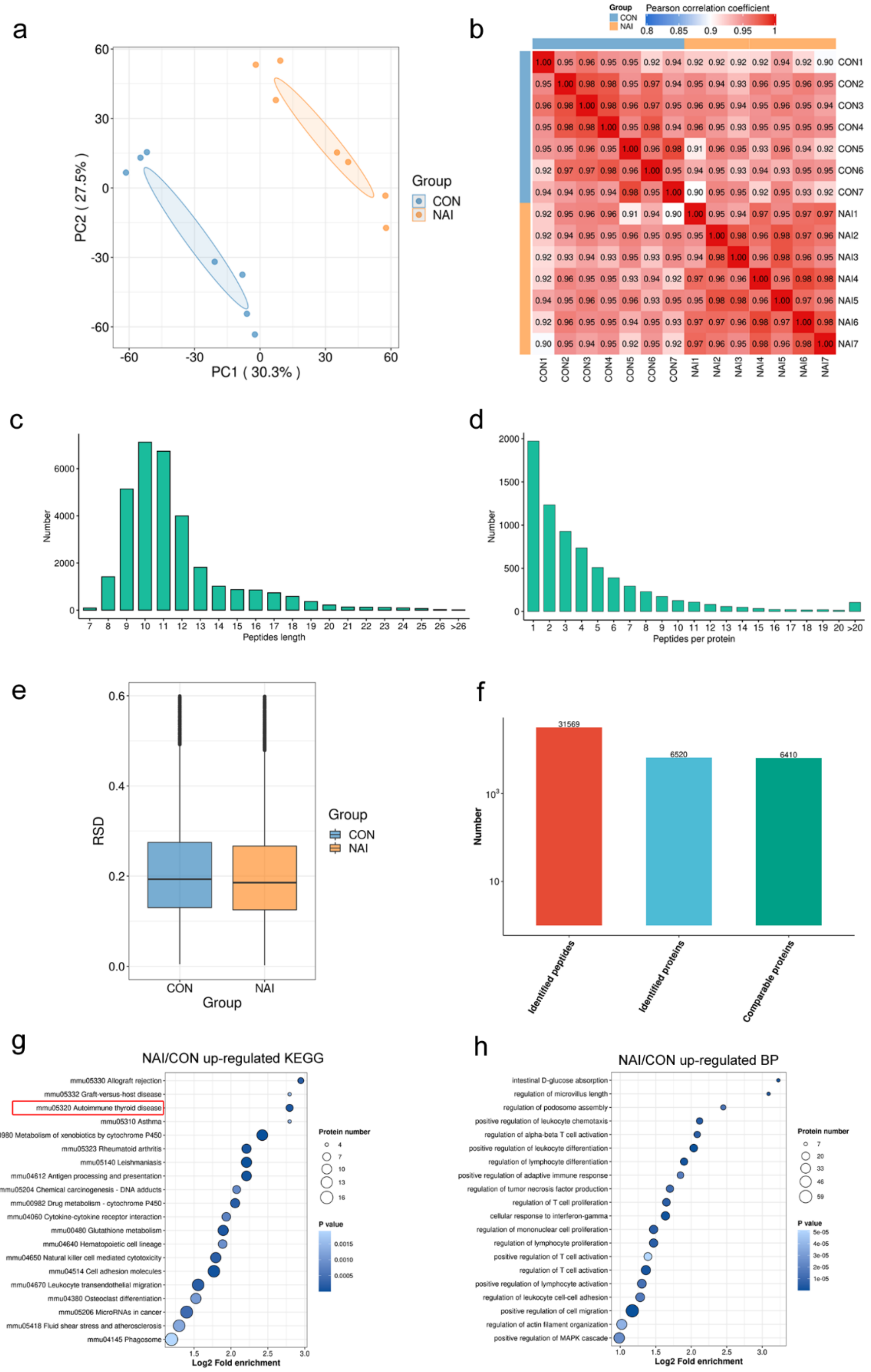

Supplementary Fig. 2 Proteomics quality control analysis of thyroid tissues from control and autoimmune thyroiditis model mice

(a) PCA of relative quantitative data. Axes show the proportion of variance explained. (b) Pairwise Pearson correlation analysis of sample intensities. Replicate samples within each group show high

correlation (red). (c) Histogram displaying the distribution of identified peptide lengths. (d) Distribution of the number of peptides identified per protein. (e) Box plot illustrating the distribution of the relative standard deviation (RSD) for relative quantification values within replicate samples of each group. (f) Bar plot summarizing key metrics of the proteomic analysis: the total number of identified peptides, unique peptides, identified proteins, and quantifiable proteins. (g) KEGG enrichment analysis in thyroid tissue for upregulated proteins comparing high-iodine-fed and normal groups. Bubble color reflects Fisher's exact test P value, and bubble size reflects the number of proteins. (h) GO biological processes (BP) enrichment analysis in thyroid tissue for upregulated proteins comparing high-iodine-fed and normal groups. Bubble color reflects Fisher's exact test *P* value, and bubble size reflects the number of proteins.

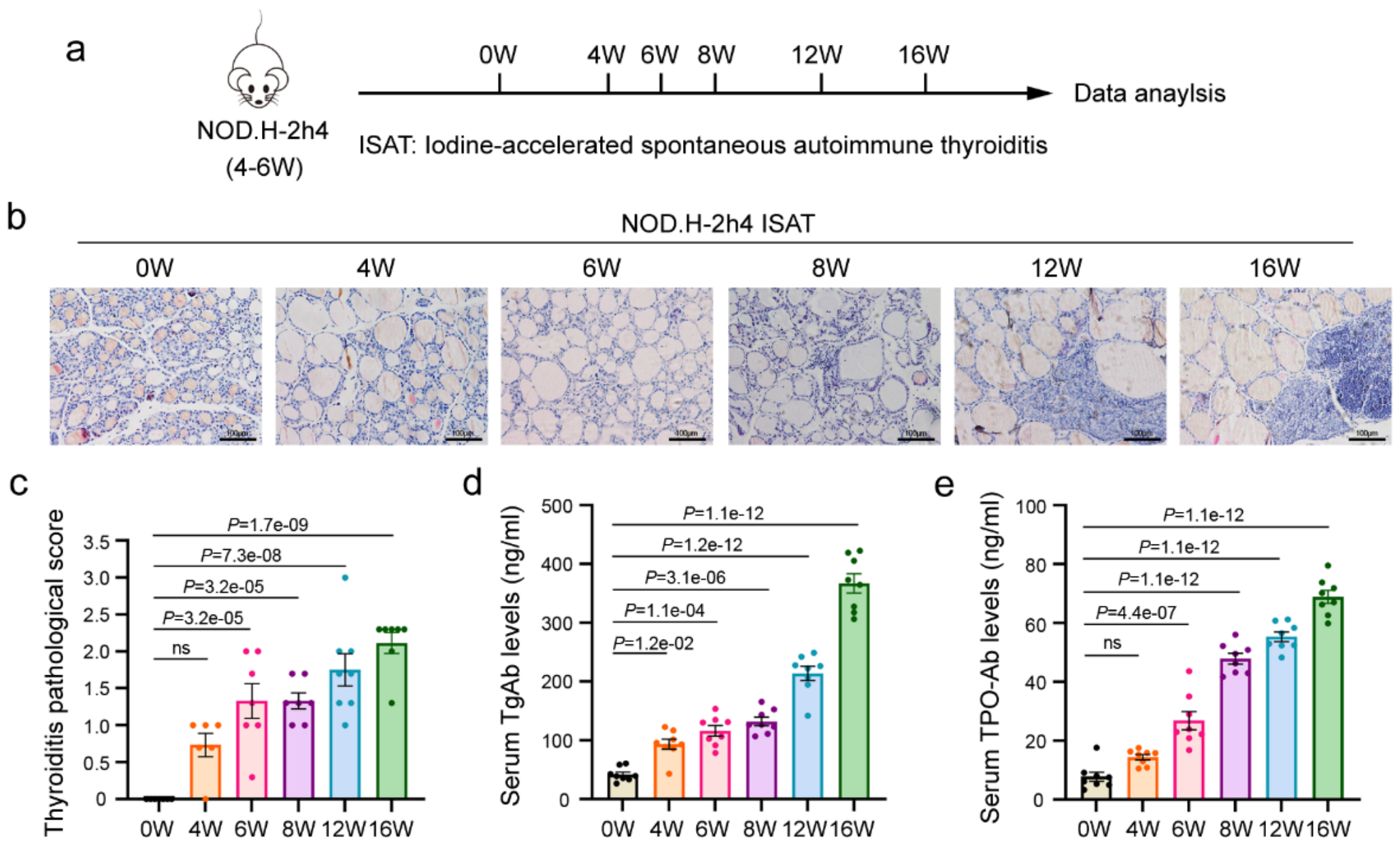

Supplementary Fig. 3 Induction of the iodine-induced spontaneous autoimmune thyroiditis (ISAT) mouse model

(a) Workflow chart showing the high iodine feeding times for NOD.H-2^{h4} mice. (b) Representative H&E-stained histology of the thyroid gland. Scale bar, 100 µm. (c) Scores of inflammatory infiltration in the thyroid. Data are presented as mean ± SEM (n = 6-8 in each group); (d, e) Serum TgAb and TPO-Ab levels were determined by ELISA. Data are presented as mean ± SEM (n = 8 in each group). Statistics for (c–e): Comparisons among multiple groups were performed using one-way ANOVA followed by Tukey's multiple comparisons test (two-sided). Adjusted *P* values are shown; ns, not significant.

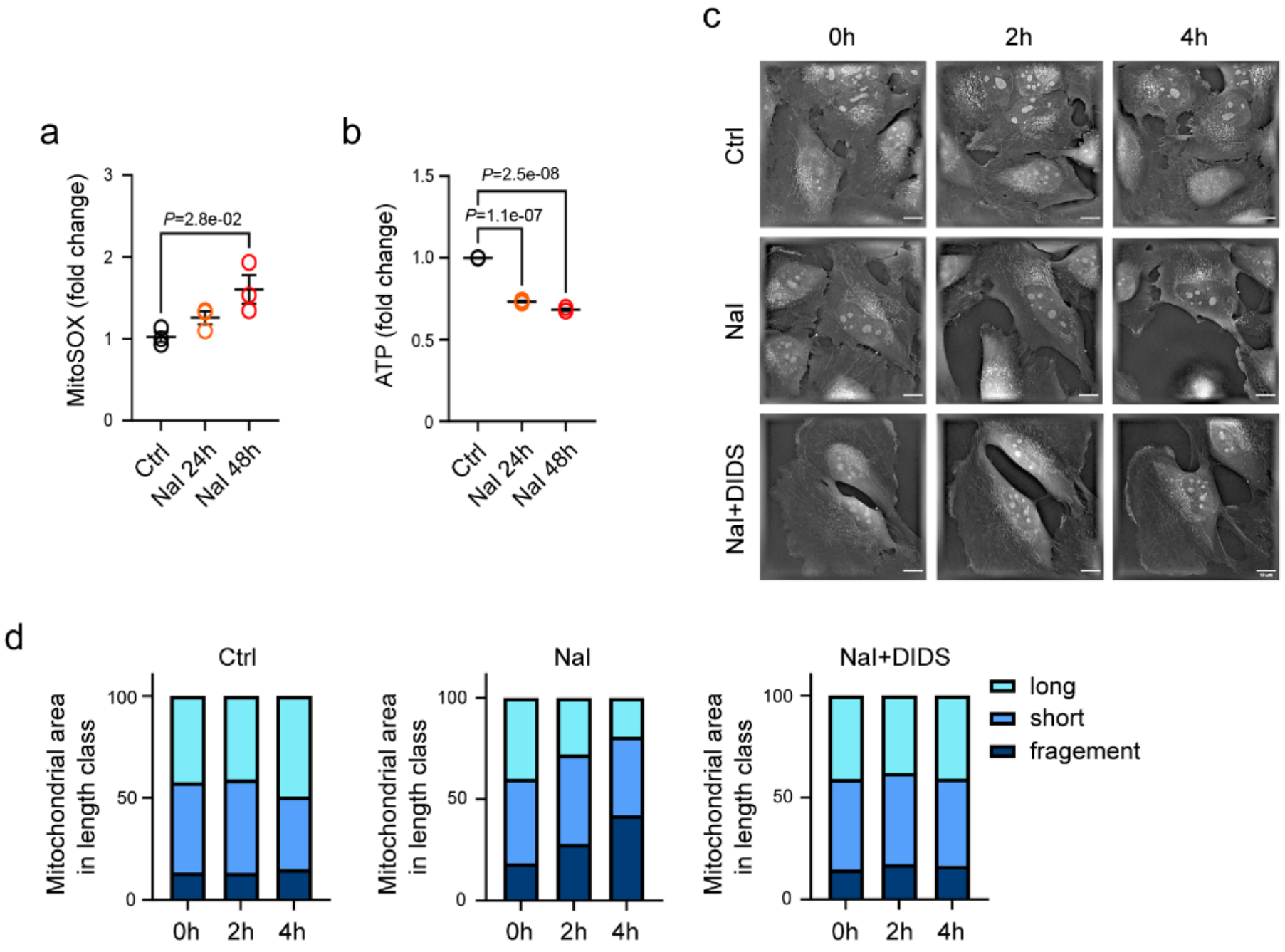

Supplementary Fig. 4 Excessive iodine induces mitochondrial dysfunction and damage in thyroid follicular epithelial cells

(a) Detection and quantification of intracellular mtROS in Nthy-ori3-1 cells after NaI treatment (40 mM) for 24 or 48 h. Data are presented as mean ± SEM from three independent experiments. (b) Detection and quantification of intracellular ATP levels in Nthy-ori3-1 cells after NAI (40 mM) treatment for 24h or 48h. Data are presented as mean ± SEM from three independent experiments. (c) Microscopic images of Nthy-ori3-1 cells under control, NaI treatment, or NaI + DIDS at 0, 2, and 4 h. Scale bar, 10 µm. (d) Stacked bar graphs illustrating the percentage of mitochondrial area categorized by length (long, short, and fragmented) in Ctrl, Nal, and Nal + DIDS groups at 0 h, 2 h, and 4 h. Statistics for (a) and (b): Comparisons among multiple groups were performed using one-way ANOVA followed by Tukey's multiple comparisons test (two-sided). Adjusted *P* values are shown.

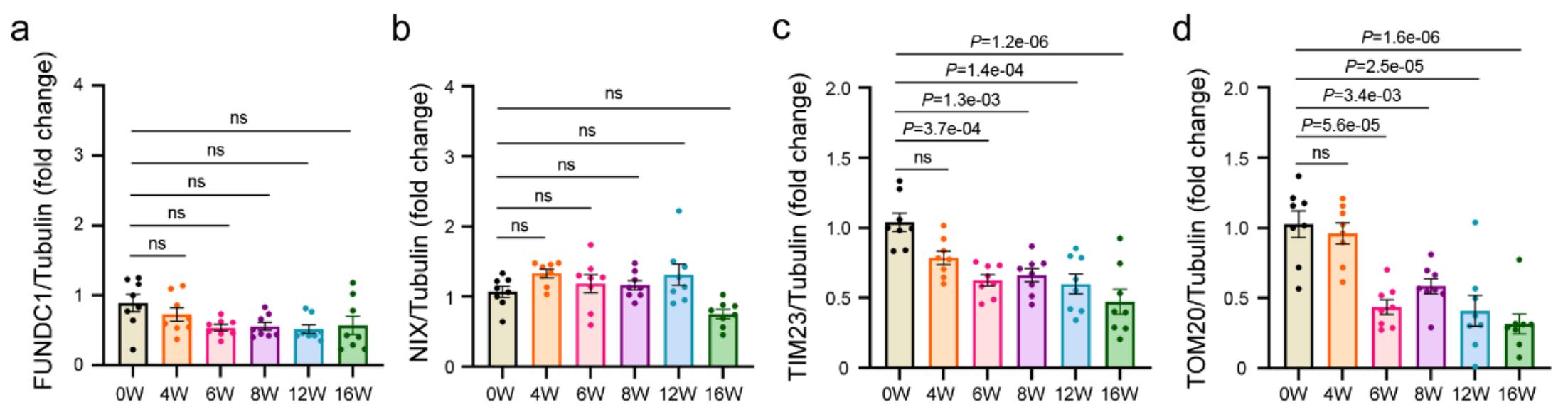

Supplementary Fig. 5 Assessment of mitophagy levels in the iodine-induced spontaneous autoimmune thyroiditis (ISAT) mouse model

(a-d) Relative expression of FUNDC1, NIX, TOM20, and TIM23 in thyroid tissue was assessed by western blot. Tubulin was used as a loading control. Data are presented as mean ± SEM (n=8 in each group). Comparisons among multiple groups were performed using one-way ANOVA followed by Tukey's multiple comparisons test (two-sided). Adjusted *P* values are shown in the graphs; ns, not significant.

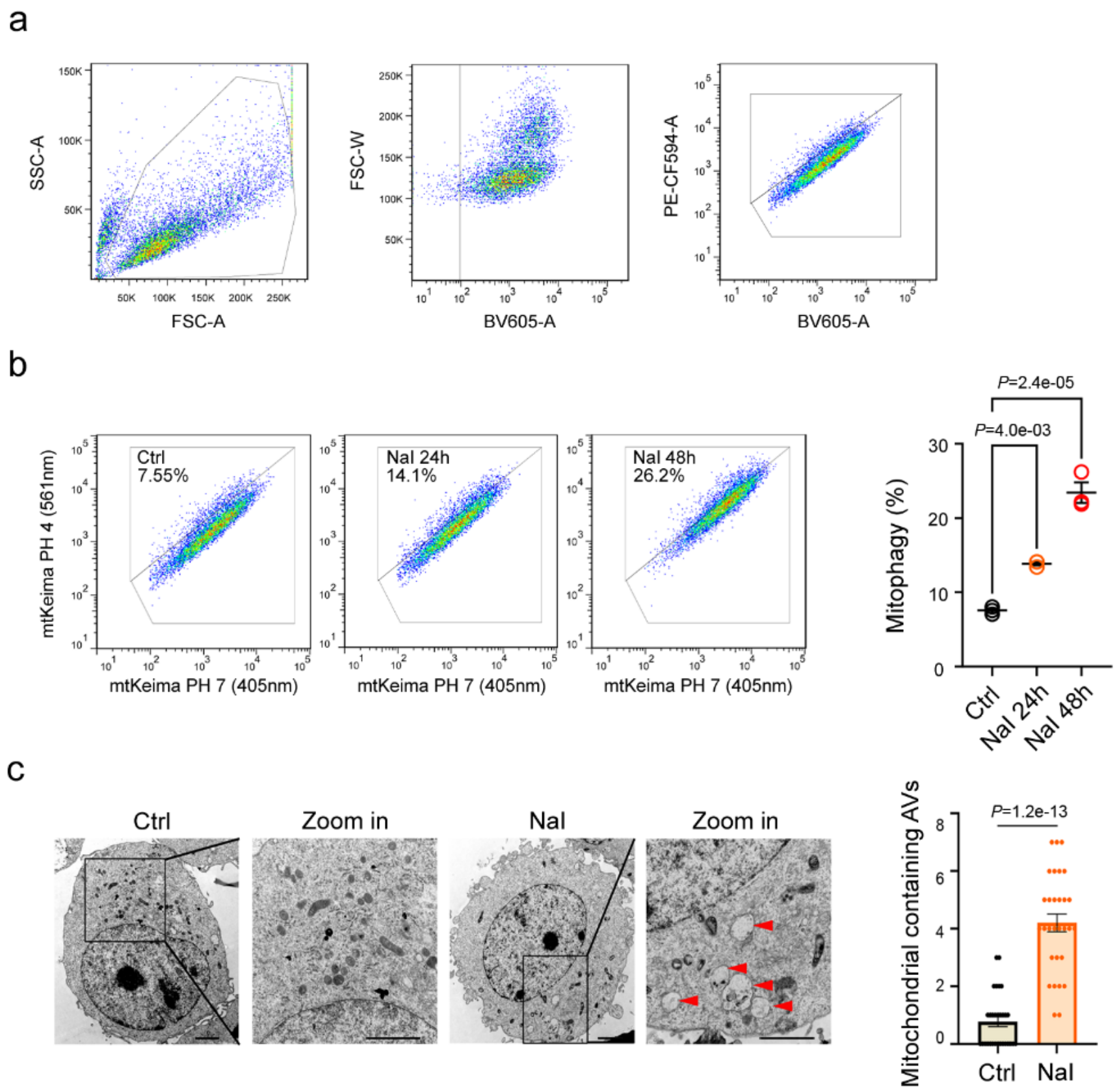

Supplementary Fig. 6 Excessive iodine induces mitophagy in thyroid follicular epithelial cells

(a) Representative flow cytometry plots and gating strategy for mt-Keima analysis. (b) Representative FACS plot (left) and quantification (right) of Nthy-ori3-1 cells stably expressing mt-Keima and subjected to NaI (40 mM) treatment for 0h, 24h or 48h. Data are presented as mean ± SEM from three independent experiments. Comparisons among multiple groups were performed using one-way ANOVA followed by Tukey's multiple comparisons test (two-sided). Adjusted *P* values are shown. (c) Representative TEM images (left) and quantification (right) of mitochondria-containing autophagic vacuoles in Nthy-ori3-1 cells cultured in normal medium or treated with NaI (40 mM). Data are presented as mean ± SEM. Scale bars: 1 μm (main image and magnified view). Comparison between two groups was performed using two-sided unpaired Student's t-test.

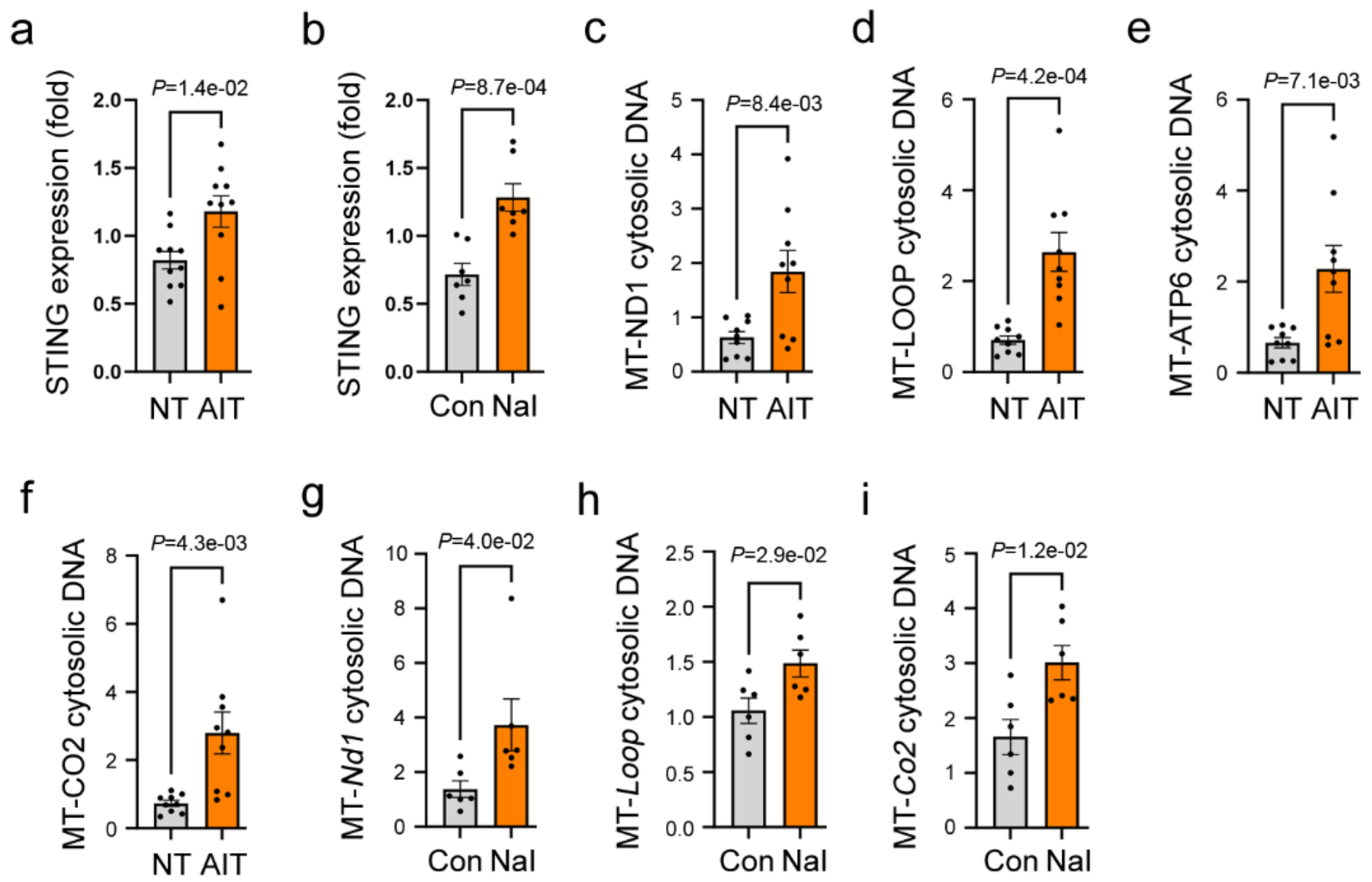

Supplementary Fig. 7 Proteomics analysis of STING expression in human and mouse models of autoimmune thyroiditis

(a) Proteomic analysis of STING expression in human thyroid tissue from healthy controls and patients with autoimmune thyroiditis. Data are presented as mean ± SEM (n=10 in each group); (b) Proteomic analysis of STING expression in thyroid tissue from control and autoimmune thyroiditis model mice. Data are presented as mean ± SEM (n=7 in each group). (c-f) Cytosolic mtDNA levels in thyroid tissues from healthy controls (NT) and AIT patients (AIT) (n = 9 per group). Specific mitochondrial DNA regions were quantified by qPCR: (c) *ND1*, (d) *LOOP*, (e) *ATP6*, and (f) *CO2*. Data are presented as mean ± SEM, presented as fold change relative to the control group (control mean set to 1). (g-i) Cytosolic mtDNA levels in thyroid tissues from control mice (Con) and ISAT mice at 6 weeks after high-iodine feeding (NaI) (n = 6 per group). Quantified regions: (g) *Nd1*, (h) *Loop*, and (i) *Co2*. Data are presented as mean ± SEM, presented as fold change relative to the control group (control mean set to 1). Statistics for (a–i): Comparisons between two groups were performed using two-sided unpaired Student's t-test. Adjusted *P* values are shown in the graphs.

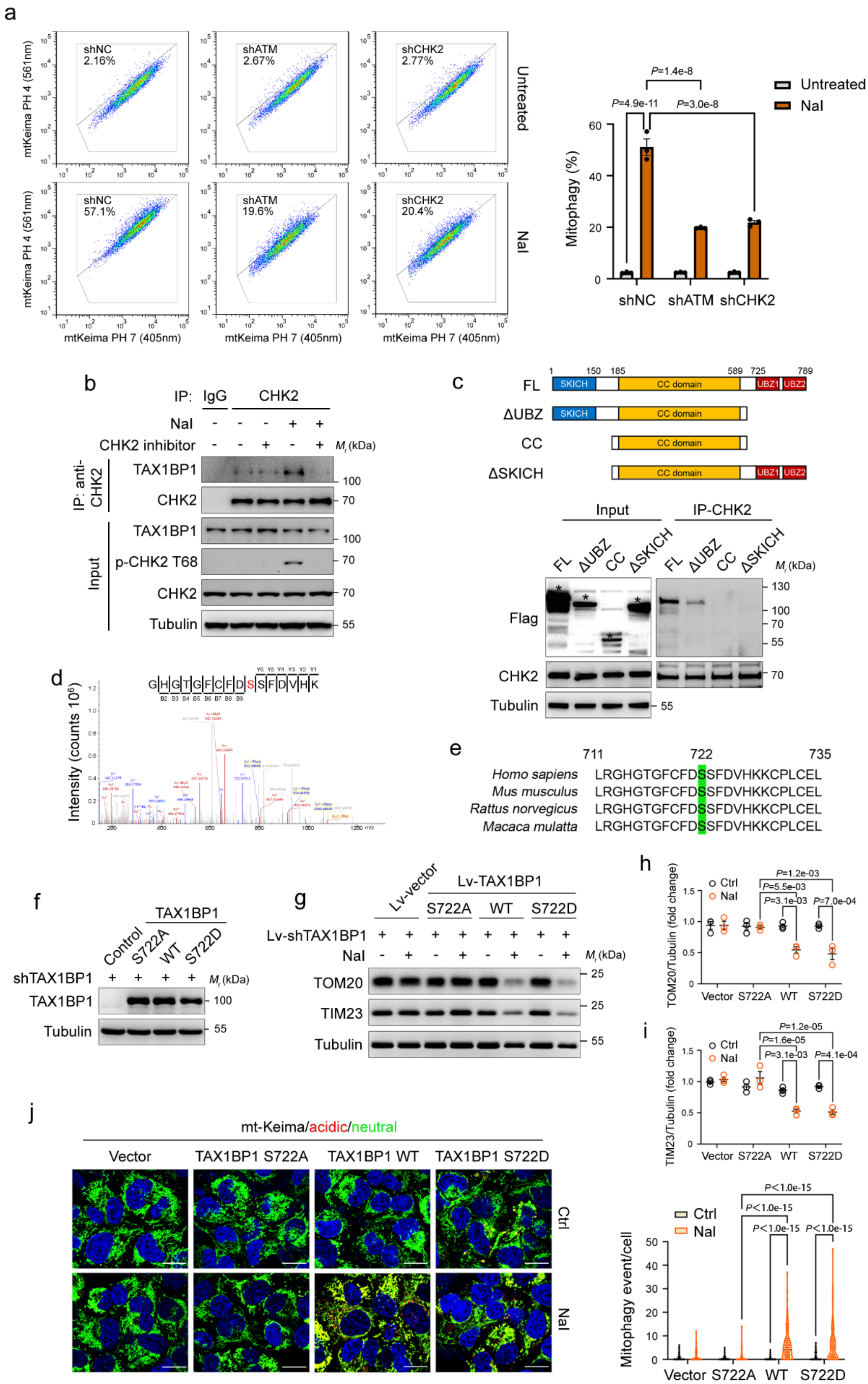
a
shNC 2.16%
shATM 2.67%
shCHK2 2.77%
shNC 57.1%
shATM 19.6%
shCHK2 20.4%
Untreated
NaI
mtKeima PH 4 (561nm)
mtKeima PH 7 (405nm)
Mitophagy (%)
P=1.4e-8
P=4.9e-11
P=3.0e-8
Untreated
NaI
shNC shATM shCHK2
b
IP: IgG CHK2
NaI
CHK2 inhibitor
IP: anti-CHK2
Input
TAX1BP1
CHK2
p-CHK2 T68
Tubulin
c
FL
ΔUBZ
CC
ΔSKICH
SKICH
CC domain
UBZ1
UBZ2
Input
IP-CHK2
Flag
CHK2
Tubulin
d
Intensity (counts 10^6)
G H G T G F C F D S S F D V H K
e
711 722 735
Homo sapiens LRGHGTGFCFDSSFDVHKKCPLCEL
Mus musculus LRGHGTGFCFDSSFDVHKKCPLCEL
Rattus norvegicus LRGHGTGFCFDSSFDVHKKCPLCEL
Macaca mulatta LRGHGTGFCFDSSFDVHKKCPLCEL
f
TAX1BP1
Control S722A WT S722D
shTAX1BP1
TAX1BP1
Tubulin
g
Lv-vector
Lv-TAX1BP1
S722A WT S722D
Lv-shTAX1BP1
NaI
TOM20
TIM23
Tubulin
h
TOM20/Tubulin (fold change)
Ctrl
NaI
P=1.2e-03
P=5.5e-03
P=3.1e-03
P=7.0e-04
Vector S722A WT S722D
i
TIM23/Tubulin (fold change)
P=1.2e-05
P=1.6e-05
P=3.1e-03
P=4.1e-04
j
mt-Keima/acidic/neutral
Vector TAX1BP1 S722A TAX1BP1 WT TAX1BP1 S722D
Ctrl
NaI
Mitophagy event/cell
P<1.0e-15
Vector S722A WT S722D

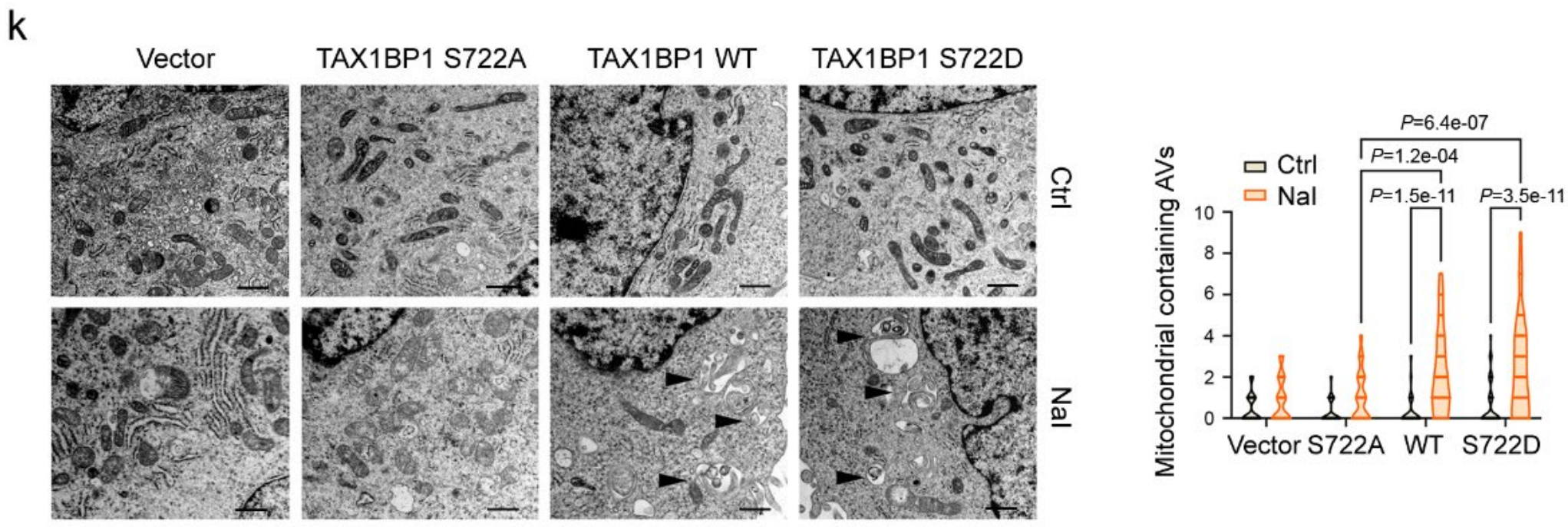

Supplementary Fig. 8 The ATM-CHK2 pathway drives mitophagy in response to mitochondrial dysfunction by phosphorylating TAX1BP1 at ser722

(a) Representative FACS plot (left) and quantification (right) of Nthy-ori3-1 cells stably expressing mt-Keima and the indicated shRNA in normal medium or after NAI (40 mM) treatment. Data are presented as mean ± SEM from three independent experiments. (b) Assessment of CHK2/TAX1BP1 binding in NaI-treated Nthy-ori 3-1 cells with or without CHK2 inhibitor. Cell lysates were immunoprecipitated (IP) with anti-CHK2 antibody and immunoblotted for TAX1BP1 and CHK2. (c) Schematic (up) representation of TAX1BP1 full length (FL) and regions encompassing the UBZ domain (ΔSKICH), CC domain (CC), SKICH domain (ΔUBZ) constructs. Nthy-ori3-1 cells were transfected with the Flag-TAX1BP1 FL, ΔSKICH, CC and ΔUBZ indicated plasmid. IP (down) was performed using anti-Flag antibody followed by immunoblotting with anti-Flag or anti-CHK2 antibody. (d) Liquid chromatography-tandem mass spectrometry/mass spectrometry (LC-MS/MS) analyses of a tryptic fragment at m/z 607.57355 Da (-1.24 mmu/ -2.04 ppm), which was matched with the +3 charged peptide 713-GHGTGFCFDSSFDVHK-728 of TAX1BP1, indicating phosphorylation of TAX1BP1 Ser 722. The XCorr score was 4.37. (e) Alignment of TAX1BP1 Ser 722 peptide sequences from different species. (f) Reconstituted expression in TAX1BP1-knockdown Nthy-ori3-1 cells using shRNA-resistant TAX1BP1 variants. (g-i) Western blot detection of TOM20 and TIM23 in Nthy-ori3-1 cells with reconstituted expression of TAX1PB1 WT, S722A mutant, or S722D mutant in normal medium or after NaI (40 mM) treatment for 24 h. Tubulin was used as a loading control. Data are presented as mean ± SEM from three independent experiments. (j) Representative confocal images (left) and quantification (right) of Nthy-ori3-1 cells stably expressing mt-Keima and the indicated plasmid following NaI (40 mM) treatment. Data are presented as mean ± SEM from three independent experiments. Scale bar, 20 μm. (k) Representative electron microscope images (left) and quantification (right) of Nthy-ori3-1 cells stably expressing the indicated plasmid and subjected to NaI (40 mM) treatment. Data are presented as mean ± SEM from three independent experiments. Scale bar, 2 μm. Statistics for (a) and (h–k): Comparisons among multiple groups were performed using two-way ANOVA followed by Tukey's multiple comparisons test (two-sided). Adjusted *P* values are shown in the graphs.

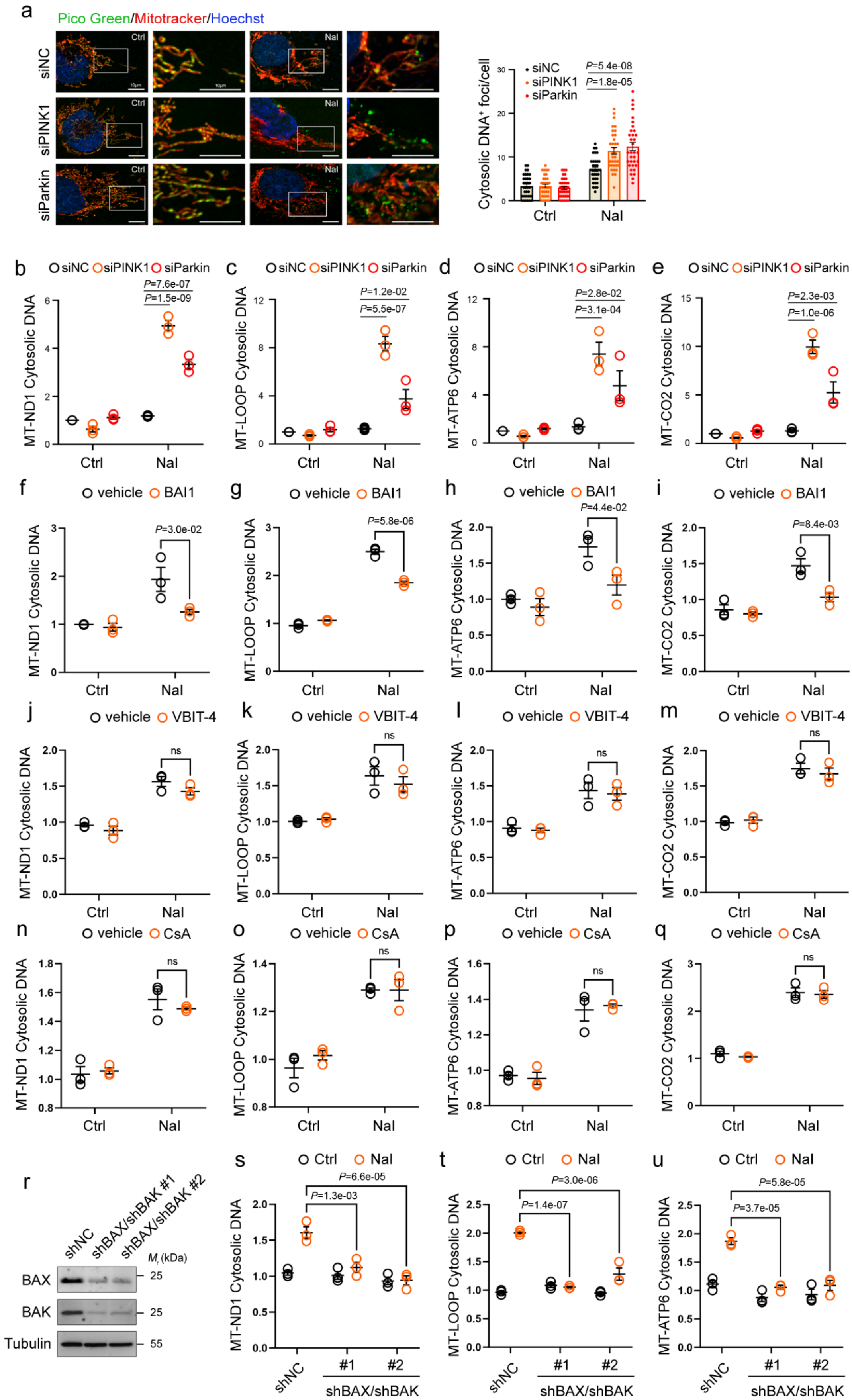

Pico Green/Mitotracker/Hoechst
siNC
siPINK1
siParkin
Ctrl
NaI
Cytosolic DNA+ foci/cell
P=5.4e-08
P=1.8e-05
MT-ND1 Cytosolic DNA
MT-LOOP Cytosolic DNA
MT-ATP6 Cytosolic DNA
MT-CO2 Cytosolic DNA
vehicle
BAI1
VBIT-4
CsA
ns
shNC
shBAX/shBAK #1
shBAX/shBAK #2
BAX
BAK
Tubulin
Mr (kDa)
shBAX/shBAK

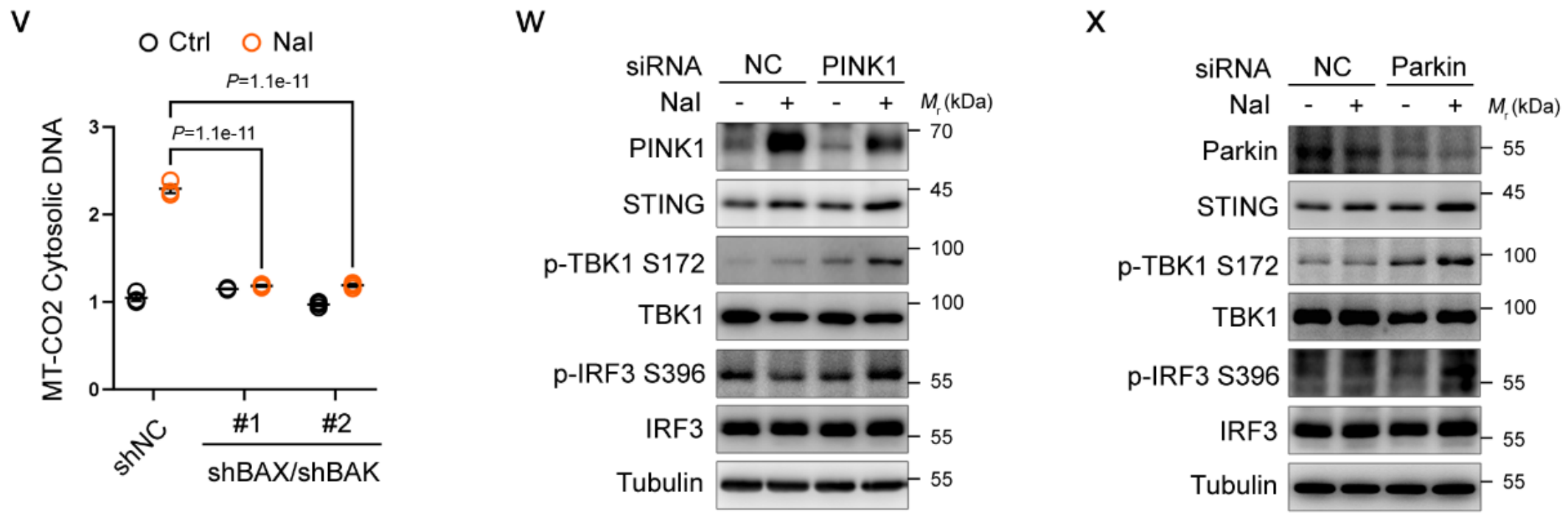

Supplementary Fig. 9 Mitophagy suppresses mtDNA release-induced cGAS-STING activation

(a) Representative confocal images (left) and quantification (right) of Nthy-ori3-1 cells transfected with the indicated siRNA, treated with or without NaI, and stained with PicoGreen (DNA) and MitoTracker (mitochondria). Scale bars: 10 µm (main image and magnified view). Data are presented as mean ± SEM from three independent experiments. (b-e) Cytosolic leakage of mtDNA was assessed by qPCR in Nthy-ori3-1 cells cultured in normal medium or treated with NaI (40 mM) for 24 h. Data are presented as mean ± SEM from three independent experiments. (f-i) Cytosolic mtDNA levels (qPCR) in Nthy-ori3-1 cells treated with NaI (40 mM, 36 h) in the presence of the BAX inhibitor BAI1 (2.5 µM). Specific mitochondrial DNA regions were quantified: (f) *ND1*, (g) *LOOP*, (h) *ATP6*, (i) *CO2*. Data are mean ± SEM from three independent experiments. (j-m) Cytosolic mtDNA levels in cells treated with NaI in the presence of the VDAC inhibitor VBIT-4 (5 µM). Quantified regions: (j) *ND1*, (k) *LOOP*, (l) *ATP6*, (m) *CO2*. Data are mean ± SEM. (n-q) Cytosolic mtDNA levels in cells treated with NaI in the presence of the mPTP inhibitor cyclosporin A (CsA, 2.5 µM). Quantified regions: (n) *ND1*, (o) *LOOP*, (p) *ATP6*, (q) *CO2*. Data are mean ± SEM; ns, not significant. (r) Western blot validation of BAX and BAK knockdown in Nthy-ori3-1 cells using specific shRNAs. Tubulin serves as loading control. (s-v) Cytosolic mtDNA levels in control shRNA-expressing cells or BAX/BAK double-knockdown cells following NaI treatment (40 mM, 36 h). Quantified regions: (s) *ND1*, (t) *LOOP*, (u) *ATP6*, (v) *CO2*. Data are mean ± SEM from three independent experiments. (w, x) Nthy-ori3-1 cells were transfected with the indicated siRNA and maintained in normal medium or treated with NaI (40 mM) for 24 h. Western blot analyses of STING, p-TBK1 (Ser172), TBK1, p-IRF3 (Ser396), and IRF3 in siPINK1 (w) and siParkin (x) cells. Tubulin was used as a loading control. Units: For panels a–q and s–v, data are presented as fold change relative to the control group (mean of control = 1). Statistics: For (a–q) and (s–v), comparisons among multiple groups were performed using two-way ANOVA followed by Tukey's multiple comparisons test (two-sided). Adjusted *P* values are shown in the graphs; ns indicates not significant.
