# Supplementary material for: Mitophagy mitigates mitochondrial DNA-induced activation of cGAS-STING in autoimmune thyroiditis
Source: Nat Commun. 2026 Jul 24;17:9041. doi: 10.1038/s41467-026-76047-9 (PMC13507228; doi:10.1038/s41467-026-76047-9)
Supplement: Supplementary file 2 [file 41467_2026_76047_MOESM2_ESM.pdf]

nature portfolio

Peer Review File

Mitophagy mitigates mitochondrial DNA-induced activation of cGAS-STING in autoimmune thyroiditis

Corresponding Author: Professor Qiqiang Guo

This file contains all reviewer reports in order by version, followed by all author rebuttals in order by version.

Version 0:

Reviewer comments:

Reviewer #1

(Remarks to the Author)
In this study by Xie and colleagues, the authors show that mitochondrial dysfunction and mitophagy are features of autoimmune thyroiditis. Using patient samples, they go on to show that cGAS-STING activation is related to clinical stage of the disease. They then show that PINK1-Parkin mitophagy can mitigate the pathogenesis of the disease in a TAX1BP1-dependent manner, and blocking this pathway induces mtDNA leakage. Finally, they show that inhibiting STING activation slows the pathology of autoimmune thyroiditis.

Overall, this study combines patient samples, relevant mouse models and cell lines to produce compelling conclusions. I find the experiments well performed, controlled and the conclusions justified. I only have a few major and minor comments.

Major comments:

- How is the mtDNA released? Is it via BAX/BAK pores? VDAC channels? MPTP? It would be interesting to test this in the cell line model.
- Is is possible to check whether there is mtDNA leakage in patient or mouse disease samples?

Minor comments:

- In Figure 1N, the authors rely on Western blotting of TOM20 and TIM23 to show mitophagic clearance of mitochondria. If these proteins samples are still available (i.e. it is not necessary to repeat the animal work for this point) it would be worth checking some other markers, for example by using the MitoProfile cocktail of antibodies. TOM20 is expressed on the outer membrane, and could be selectively extracted, so proteins inside the mitochondria are more relevant here. Similarly, if the samples are available, other methods for mitochondrial mass could be used, e.g. qPCR for mtDNA.
- In general, more information about the experiments could be included in the text. For example, in Figure 1, it would be useful to see examples of what the authors counted as a “damaged mitochondria”, in the figure (or supplemental figure), or in the text - or better, in the figure and the text.
- In Figure 3A, if these samples are still available, alternative ways of showing a decrease in mitochondria would be useful.
- In Figure 3C,D, please point out more clearly what makes a damaged mitochondria damaged.
- In Figure 4H, there is a small typo - it should be K63, not K68
- In Figure 5D, the authors assess mtDNA release into the cytosol. In the vector sample and the S722A sample, treated with NaI, there appears to be a grouping of mtDNA release, reminiscent of mtDNA nucleoids in Drp1 deficient cells (PMID 23821750). Is this something the authors have considered?

Reviewer #2

(Remarks to the Author)
In their study, Xie et al. describe a mechanism in which mitochondrial dysfunction in autoimmune thyroiditis (AT) leads to the cytoplasmic release of mtDNA, which in turn triggers the cGAS–STING pathway, resulting in inflammasome activation and the release of inflammatory cytokines that drive immune infiltration. In contrast, mitophagy activated by the ATM–CHK2–TAX1BP1 pathway acts as a protective mechanism that prevents the release of mtDNA. Using the iodine-accelerated spontaneous autoimmune thyroiditis mouse model, the authors demonstrate that activation of the PINK1–Parkin pathway

triggers mitophagy as an adaptive response to mitochondrial dysfunction and damage. Finally, they show that pharmacological inhibition of the STING pathway attenuates thyroiditis severity in the mouse model. They also propose that "enhancing mitophagy may be a helpful therapeutic strategy to disrupt the feed-forward cycle of inflammation and tissue damage".

The study is well designed, with an elegant set of carefully conducted experiments involving mouse models, in vitro experiments using a human thyroid cell line, and analyses of tissue derived from human patients. All statements and conclusions are well supported by the experimental results.

However, it should be noted that the authors do not cite previous studies that have already demonstrated the significance of the STING pathway in AT, e.g. Shen et al. J Transl Autoimmun. 2025 Mar 22;10:100284; Ding et al. Tissue Cell. 2024 Dec;91:102596. In particular, the most striking omission is the failure to cite the paper by Lee et al., published in Thyroid. 2024 Nov;34(11):1401–1413, who demonstrated that loss of Pink1 is a driver of Hashimoto's thyroiditis and is associated with mitochondrial abnormalities, mitophagy defects, and upregulation of DAMP-related genes including NLRP3, STING, and NF-κB, along with increased release of pro-inflammatory cytokines that promote recruitment of immune cells to the thyroid. Moreover, Lee et al. concluded that "impaired mitophagy-induced inflammation by excessive mtROS and DAMPs may be an additional stressor that exacerbates the autoimmune response", a conclusion very similar to that stated by Xie et al.

Other comments:

The manuscript lacks a clearly stated aim and hypothesis.

Line 260: "Confocal microscopy quantification of cytosolic dsDNA foci (PicoGreen) and mitochondrial mass (MitoTracker) showed significant accumulation of extramitochondrial DNA in cells expressing the phospho-deficient TAX1BP1 S722A mutant compared with WT ...": Did the authors observe such extramitochondrial DNA in tissues from AT mice or human patients?

Several methods are described only briefly. For example:

Line 456, Western blot and IP analysis: "Protein extraction from cultured cells was performed with IP lysis buffer followed by IP and immunoblotting using primary antibodies as described previously15." Reference 15 does not provide a detailed Western blot procedure (e.g. whether nitrocellulose or PVDF membranes were used, how membranes were blocked and incubated with antibodies, and how the signal was developed). Please provide the missing methodological details to enable reproducibility.

Line 482: "Stable cell lines expressing mt-Keima were generated using a lentiviral system." Please provide a detailed description of this method.

Figure 4h: Please correct the spelling error (K68 should be changed to K63).

Version 1:

Reviewer comments:

Reviewer #1

(Remarks to the Author)

I am satisfied with the authors' responses and the revisions made, and I have no further concerns regarding the manuscript.

Reviewer #2

(Remarks to the Author)

Dear Authors,

Thank you for addressing all my comments and questions. The new data demonstrating the presence of extramitochondrial DNA in tissues from AT mice and human patients are clear and convincing.

However, a detailed description of the methods used to generate these data is still missing. Please provide a comprehensive description of the qPCR procedures, including DNA isolation methods, primer sequences, and the qPCR kits used, as well as the method employed for calculating the amount of detected DNA (e.g., absolute quantification, ΔΔCt, Pfaffl, or another approach).

In addition, please clarify the units used to represent DNA amounts in the plots shown in Figures 5a–e, Supplementary Figures 7c–i, and Supplementary Figures 9a–q and 9s–v. (This information can be provided either in the Methods section or in the figure legends).

Open Access This Peer Review File is licensed under a Creative Commons Attribution 4.0 International License, which permits use, sharing, adaptation, distribution and reproduction in any medium or format, as long as you give appropriate credit to the original author(s) and the source, provide a link to the Creative Commons license, and indicate if changes were made.
In cases where reviewers are anonymous, credit should be given to 'Anonymous Referee' and the source.
The images or other third party material in this Peer Review File are included in the article's Creative Commons license, unless indicated otherwise in a credit line to the material. If material is not included in the article's Creative Commons license and your intended use is not permitted by statutory regulation or exceeds the permitted use, you will need to obtain permission directly from the copyright holder.
To view a copy of this license, visit https://creativecommons.org/licenses/by/4.0/

Point by Point Response to Reviewers:

Reviewer #1:

Comments:

In this study by Xie and colleagues, the authors show that mitochondrial dysfunction and mitophagy are features of autoimmune thyroiditis. Using patient samples, they go on to show that cGAS-STING activation is related to clinical stage of the disease. They then show that PINK1-Parkin mitophagy can mitigate the pathogenesis of the disease in a TAX1BP1-dependent manner, and blocking this pathway induces mtDNA leakage. Finally, they show that inhibiting STING activation slows the pathology of autoimmune thyroiditis.

Overall, this study combines patient samples, relevant mouse models and cell lines to produce compelling conclusions. I find the experiments well performed, controlled and the conclusions justified. I only have a few major and minor comments.

We sincerely thank the reviewer for the thoughtful and positive assessment of our work. We are delighted that the reviewer finds our study compelling and the experiments well-controlled and appropriately interpreted. We greatly appreciate the constructive suggestions and valuable insights provided. In the revised manuscript, we have carefully addressed all the comments, and we believe these revisions have further strengthened the scientific rigor and clarity of our study. We hope the revised version now meets the reviewer's expectations.

Major comments:

- How is the mtDNA released? Is it via BAX/BAK pores? VDAC channels? MPTP? It would be interesting to test this in the cell line model.

Response: We thank the reviewer for this important mechanistic question. To address how mtDNA is released into the cytosol in our cell line model, we performed a series of inhibitor and genetic loss-of-function experiments. Specifically, we treated Nthy-ori3-1 cells with NaI in the presence of a BAX inhibitor (BAI1), a VDAC inhibitor (VBIT-4), or the mPTP inhibitor cyclosporin A (CsA). Cytosolic mtDNA levels were then quantified by qPCR. As now shown in new Supplementary Figure 9, neither VBIT-4 nor CsA reduced mtDNA release, whereas BAI1 significantly decreased the amount of mtDNA in the cytosol. To confirm the specificity of the BAX/BAK pathway, we knocked down BAX and BAK using shRNA. Silencing of BAX and BAK also markedly suppressed mtDNA leakage upon NaI treatment. These results indicate that mtDNA release in our model occurs primarily through BAX/BAK-dependent pores, rather than through VDAC channels or the mPTP.

Below we provide the proposed new paragraph for the Results section and the corresponding figure legend, which we have incorporated into the revised manuscript.

"We next investigated the molecular mechanism by which mtDNA is released into the cytosol under conditions of impaired mitophagy. mtDNA can be released through several distinct channels, including BAX/BAK macropores, oligomerized VDAC, and the mitochondrial permeability transition pore (mPTP). To determine which of these pathways is responsible in our model, we treated Nthy-ori3-1 cells with NaI in the presence of a BAX inhibitor (BAI1, 2.5 μM), a VDAC inhibitor (VBIT-4, 5 μM), or the mPTP inhibitor (cyclosporin A, CsA, 2.5 μM). Cytosolic fractions were isolated, and mtDNA levels were quantified by qPCR. As shown in Supplementary Figure 9f-q, BAI1 significantly reduced the amount of mtDNA in the cytosol, whereas VBIT-4 and CsA had no effect. To further validate the involvement of BAX/BAK, we knocked down BAX and BAK using specific shRNAs. Silencing of BAX and BAK substantially

attenuated NaI-induced mtDNA leakage (Supplementary Fig. 9r-v). These results demonstrate that mtDNA release in our model occurs primarily through BAX/BAK-dependent pores, rather than through VDAC channels or the mPTP."

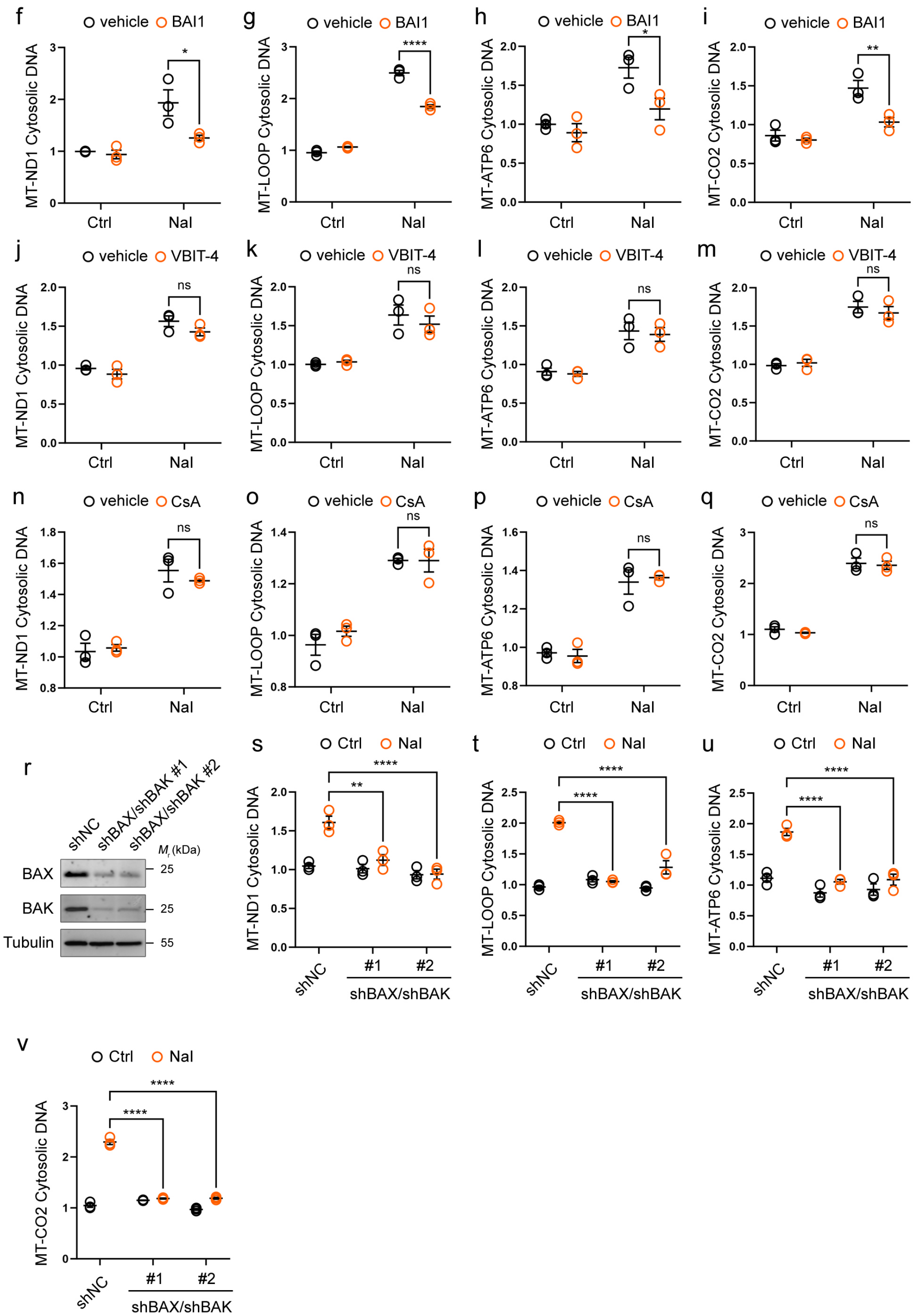

(f-i) Cytosolic mtDNA levels (qPCR) in Nthy-ori3-1 cells treated with NaI (40 mM, 36 h) in the presence of the BAX inhibitor BAI1 (2.5 μM). Specific mitochondrial DNA regions were quantified: (f) *ND1*, (g) *LOOP*, (h) *ATP6*, (i) *CO2*. Data are mean ± SEM from three independent experiments. *p < 0.05; **p < 0.01; ****p < 0.0001.
(j-m) Cytosolic mtDNA levels in cells treated with NaI in the presence of the VDAC inhibitor VBIT-4 (5 μM). Quantified regions: (j) *ND1*, (k) *LOOP*, (l) *ATP6*, (m) *CO2*. Data are mean ± SEM; ns, not significant.
(n-q) Cytosolic mtDNA levels in cells treated with NaI in the presence of the mPTP inhibitor cyclosporin A (CsA, 2.5 μM). Quantified regions: (n) *ND1*, (o) *LOOP*, (p) *ATP6*, (q) *CO2*. Data are mean ± SEM; ns, not significant.
(r) Western blot validation of BAX and BAK knockdown in Nthy-ori3-1 cells using specific shRNAs. Tubulin serves as loading control.
(s-v) Cytosolic mtDNA levels in control shRNA-expressing cells or BAX/BAK double-knockdown cells following NaI treatment (40 mM, 36 h). Quantified regions: (s) *ND1*, (t) *LOOP*, (u) *ATP6*, (v) *CO2*. Data are mean ± SEM from three independent experiments. **p < 0.01; ****p < 0.0001.

We have incorporated these new data and the corresponding text into the revised manuscript. We believe these findings directly answer the reviewer's question and further strengthen our mechanistic model.

- Is it possible to check whether there is mtDNA leakage in patient or mouse disease samples?

Response: We thank the reviewer for this important question. We fully agree that directly detecting mtDNA leakage in patient and mouse disease samples would greatly strengthen the translational relevance of our findings.

Following the reviewer's suggestion, we have now performed additional experiments. Using quantitative PCR, we measured cytoplasmic mtDNA levels in thyroid tissues from autoimmune thyroiditis patients (compared to healthy controls) and in thyroid tissues from the ISAT mouse model (compared to control mice). Significant mtDNA leakage was detected in both contexts. These data are now included in the revised manuscript as Supplementary Figure 7c-f (patient data) and Supplementary Figure 7g-i (mouse model data).

These results provide direct evidence that mtDNA leakage is a genuine pathological feature of autoimmune thyroiditis in both humans and mice, further supporting the pathophysiological relevance of the mtDNA-cGAS-STING axis identified in our study.

We believe these new data adequately address the reviewer's concern.

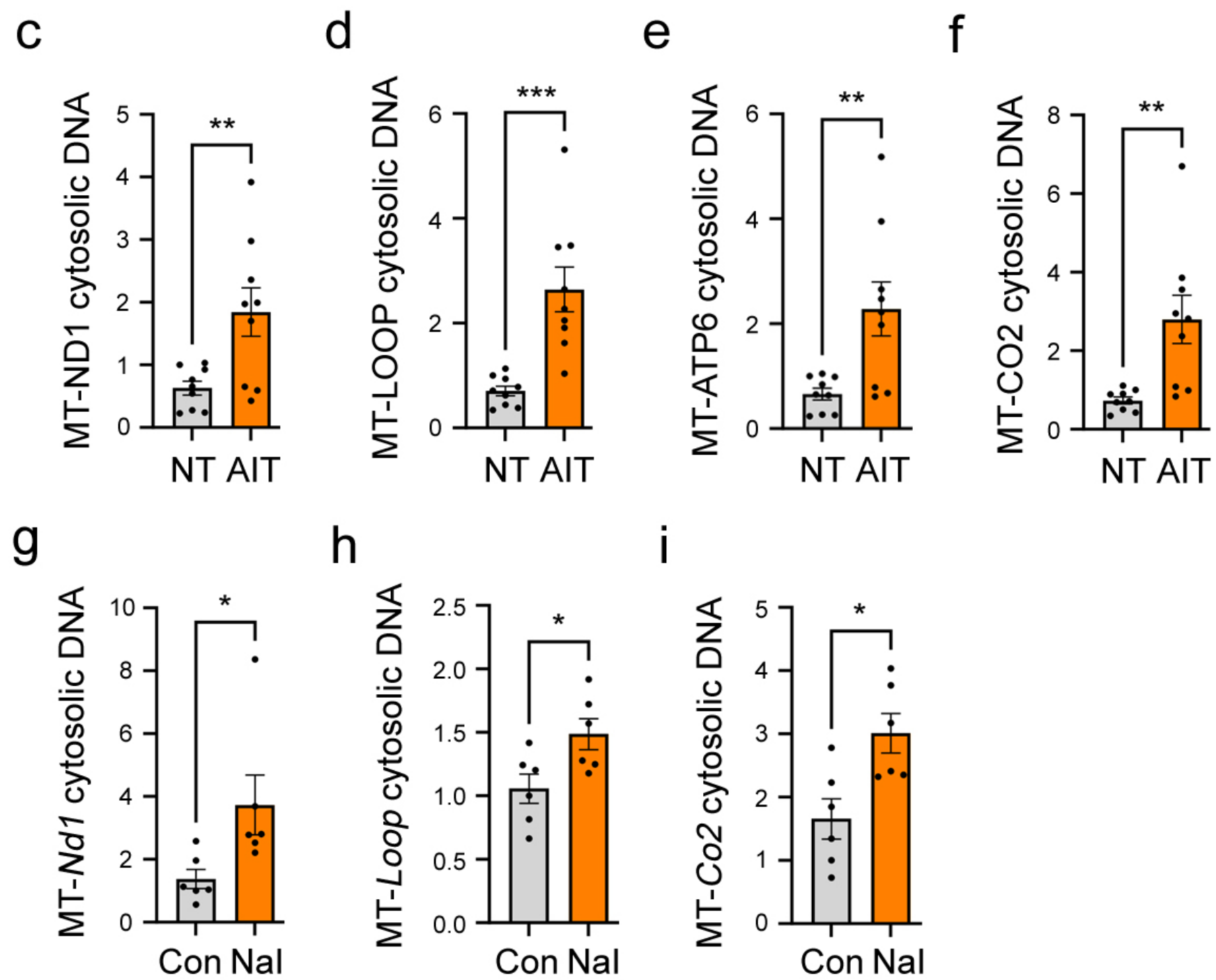

(c-f) Cytosolic mtDNA levels in thyroid tissues from healthy controls (NT) and AIT patients (AIT) (n = 9 per group). Specific mitochondrial DNA regions were quantified by qPCR: (c) *ND1*, (d) *LOOP*, (e) *ATP6*, and (f) *CO2*. Data are presented as mean ± SEM. **p < 0.01; ***p < 0.001.
(g-i) Cytosolic mtDNA levels in thyroid tissues from control mice (Con) and ISAT mice at 6 weeks after high-iodine feeding (NaI) (n = 6 per group). Quantified regions: (g) *Nd1*, (h) *Loop*, and (i) *Co2*. Data are presented as mean ± SEM. *p < 0.05.

Minor comments:
- In Figure 1N, the authors rely on Western blotting of TOM20 and TIM23 to show mitophagic clearance of mitochondria. If these proteins samples are still available (i.e. it is not necessary to repeat the animal work for this point) it would be worth checking some other markers, for example by using the MitoProfile cocktail of antibodies. TOM20 is expressed on the outer membrane, and could be selectively extracted, so proteins inside the mitochondria are more relevant here. Similarly, if the samples are available, other methods for mitochondrial mass could be used, e.g. qPCR for mtDNA.

Response: We thank the reviewer for this insightful suggestion. We fully agree that additional validation of mitophagic clearance would strengthen our conclusion, and we appreciate the recommendation to include additional markers or methods.

Regarding the availability of samples, we regret to inform that the protein lysates from the mouse thyroid tissues used in Figure 1n have been exhausted. Due to the limited size of mouse thyroid glands, insufficient material remains for further immunoblotting with the MitoProfile antibody cocktail or for mtDNA qPCR analysis.

However, as part of our original experimental design, we performed transmission electron microscopy (TEM) to directly visualize mitophagy in thyroid tissues across different time points

of NaI administration. These data were presented in Figure 1m of the original submission, showing a time-dependent increase (from 0 to 16 weeks) in the number of autophagic vacuoles containing damaged mitochondria. Representative images of autophagic vacuoles engulfing damaged mitochondria are shown below. This ultrastructural evidence directly demonstrates the progressive activation of mitophagy during the course of autoimmune thyroiditis.

Importantly, the TEM findings are fully consistent with our immunoblotting results (Figure 1n-p), which showed decreased TOM20/TIM23 levels accompanied by increased PINK1 and Parkin expression in WT-ISAT mice. Together, these two independent lines of evidence—direct visualization of autophagosome-engulfed mitochondria by TEM and changes in key mitophagy regulators by immunoblotting—provide complementary and robust support for the activation of PINK1/Parkin-dependent mitophagy in our model.

In the revised manuscript, we have now explicitly linked these two datasets in the Results section to better highlight the consistency between the TEM and immunoblotting findings. We believe this addresses the reviewer's concern regarding the validation of mitophagic clearance.

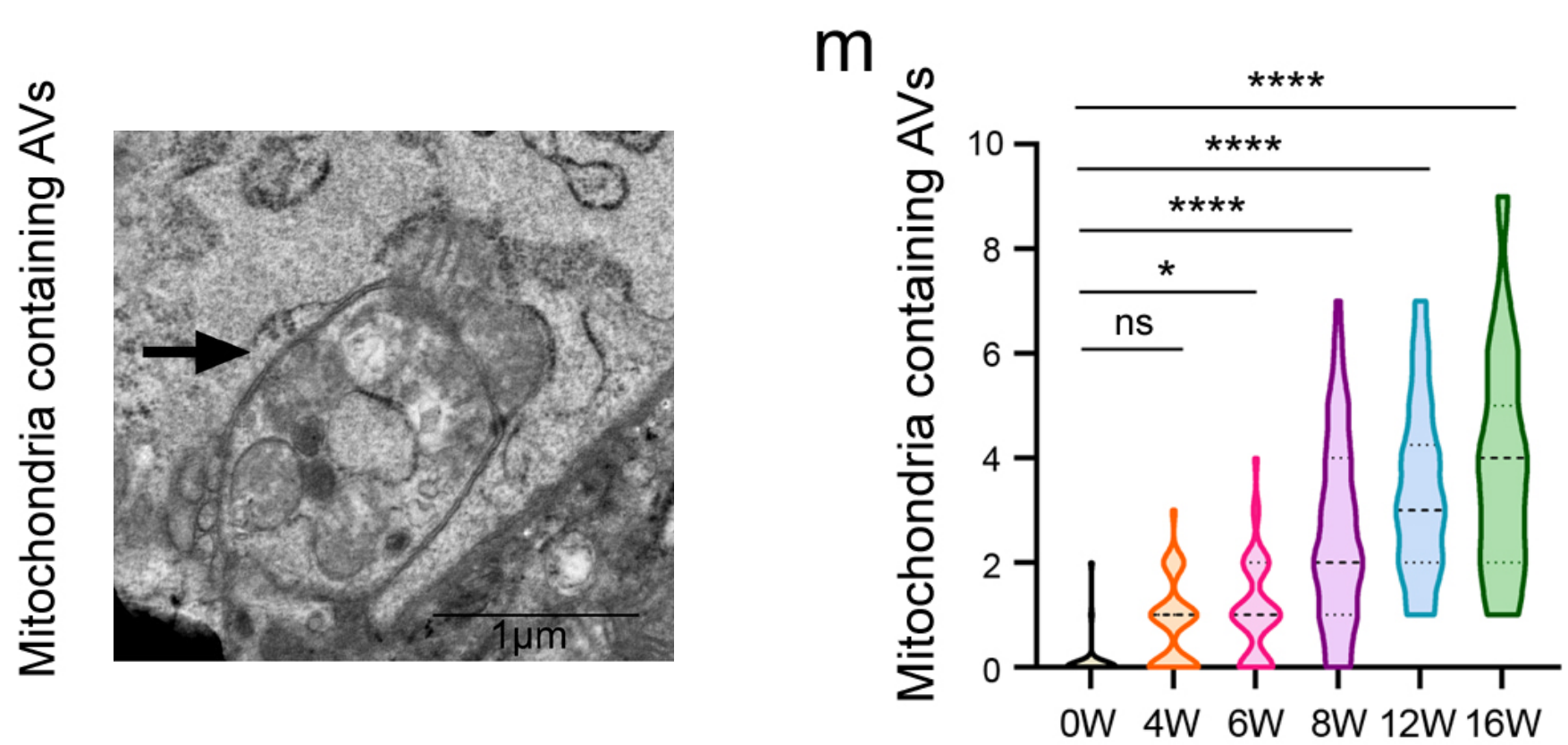

- In general, more information about the experiments could be included in the text. For example, in Figure 1, it would be useful to see examples of what the authors counted as a "damaged mitochondria" in the figure (or supplemental figure), or in the text - or better, in the figure and the text.

Response: We thank the reviewer for this helpful suggestion. We agree that clearly defining what we counted as "damaged mitochondria" will improve the transparency of our quantification. In the revised manuscript, we have: (1) added explicit morphological criteria for damaged mitochondria in the Materials and Methods section (loss of cristae, matrix swelling, and/or outer membrane rupture), which are consistent with established methods for evaluating mitochondrial integrity as described in previous studies (e.g., Ranjbarvaziri et al., Circulation, 2021); (2) annotated the representative TEM images in Figure 1k with red arrows to indicate damaged mitochondria and blue arrows to indicate intact mitochondria, with the figure legend updated accordingly; and (3) included magnified views of representative damaged and intact mitochondria to better illustrate the morphological features used for quantification. We believe these additions address the reviewer's concern.

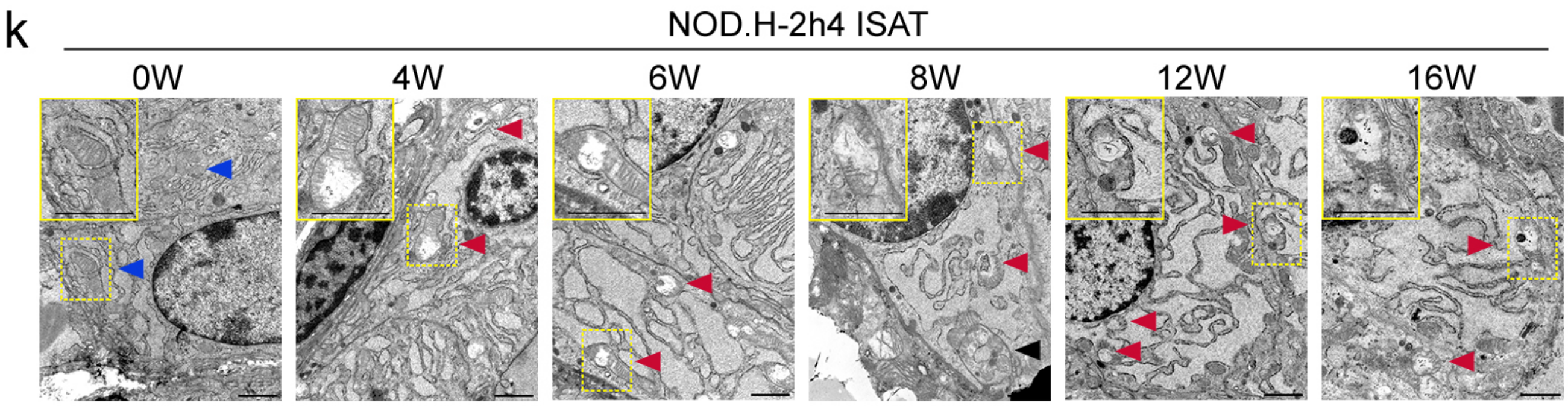

(k) Representative transmission electron microscopy images of mouse thyroid tissue from the indicated groups. Red arrows indicate damaged mitochondria (characterized by disrupted cristae and reduced cristae density), blue arrows indicate intact mitochondria, and black arrows indicate autophagosomes engulfing damaged mitochondria. Scale bar, 1 μm.

- In Figure 3A, if these samples are still available, alternative ways of showing a decrease in mitochondria would be useful.

Response: We thank the reviewer for this thoughtful suggestion. We agree that providing additional evidence to demonstrate the reduction in mitochondrial content would strengthen the conclusions drawn from Figure 3A. Unfortunately, the protein lysates used in Figure 3A have been exhausted due to the limited size of mouse thyroid tissues. As an alternative, we have now included transmission electron microscopy (TEM) data in the revised manuscript. As shown in Figure 3C and the newly added quantification, we counted autophagic vacuoles containing damaged mitochondria in WT-ISAT, *Pink1* KO ISAT, and *Park2* KO ISAT mice. Representative images of autophagic vacuoles engulfing damaged mitochondria are shown below. WT-ISAT mice showed significantly increased autophagosome-engulfed mitochondria, whereas this increase was markedly reduced in both knockout groups, directly confirming that the reduction in mitochondrial proteins reflects genuine mitophagic clearance.

We have now incorporated these TEM data and the corresponding quantification into the revised Results sections, and we believe this additional evidence adequately addresses the reviewer's concern regarding the demonstration of mitochondrial reduction.

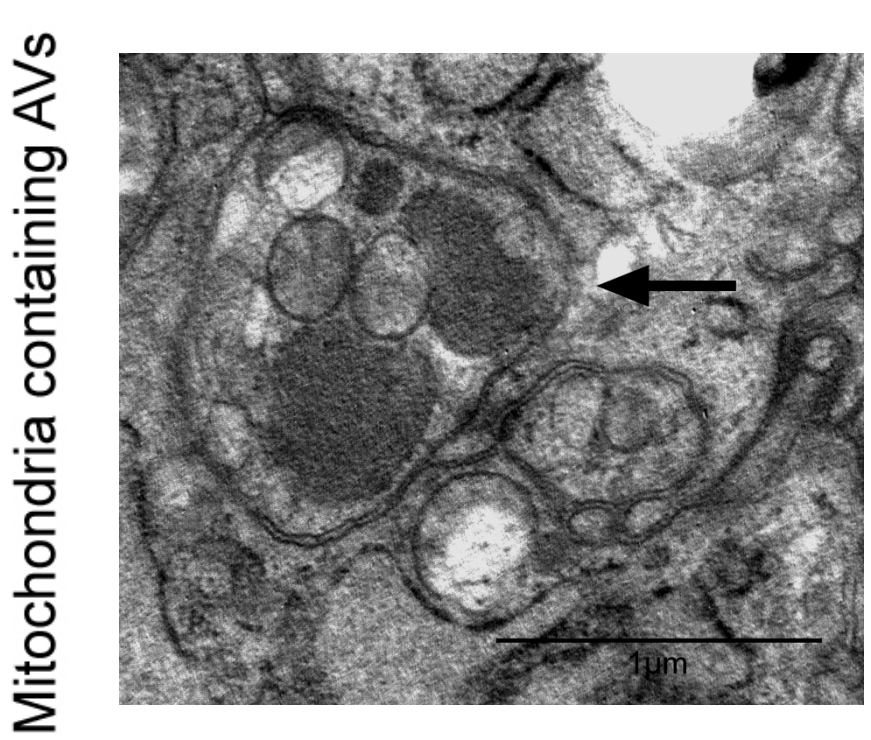

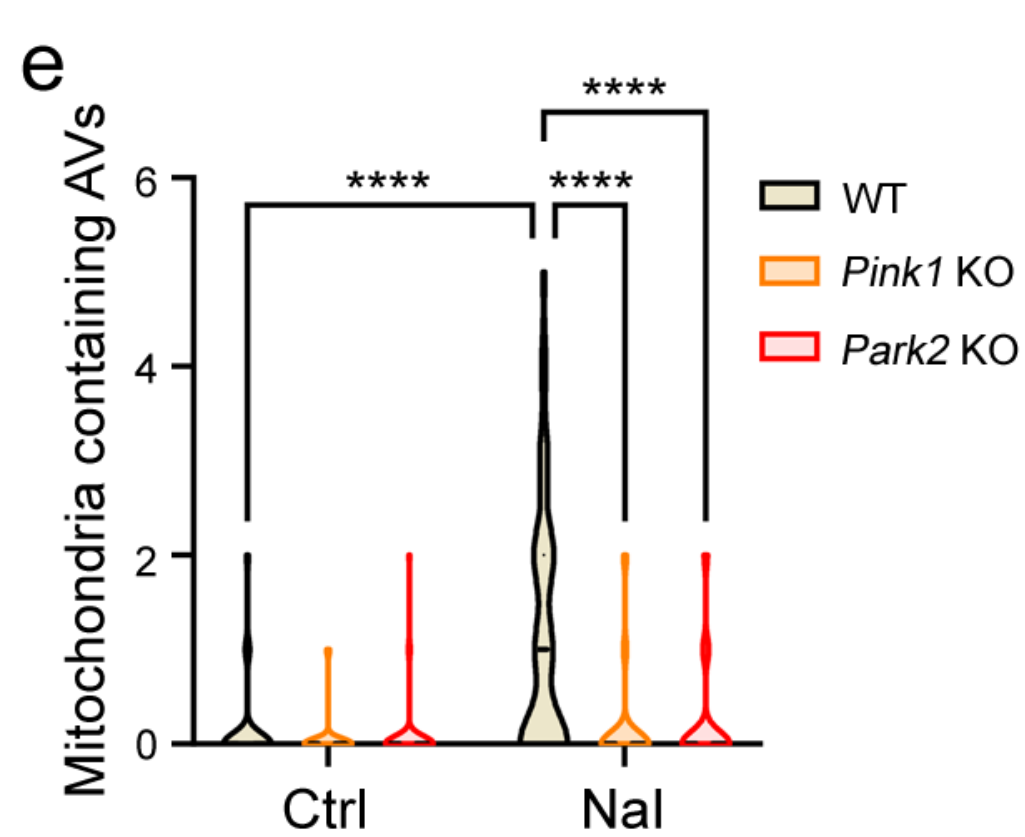

- In Figure 3C, D, please point out more clearly what makes a damaged mitochondria damaged.

Response: We thank the reviewer for this comment. As with Figure 1, we have now explicitly defined the morphological criteria for "damaged mitochondria" in the Materials and Methods section (loss of cristae structure, matrix swelling, and/or outer membrane rupture). In Figure 3c, we have added arrows to directly indicate representative damaged versus intact mitochondria in the TEM images, with red arrows indicating damaged mitochondria and blue arrows indicating intact mitochondria. Additionally, we have included magnified views of representative damaged and intact mitochondria to better illustrate the morphological features used for quantification. These revisions clarify our quantification and ensure consistency across figures.

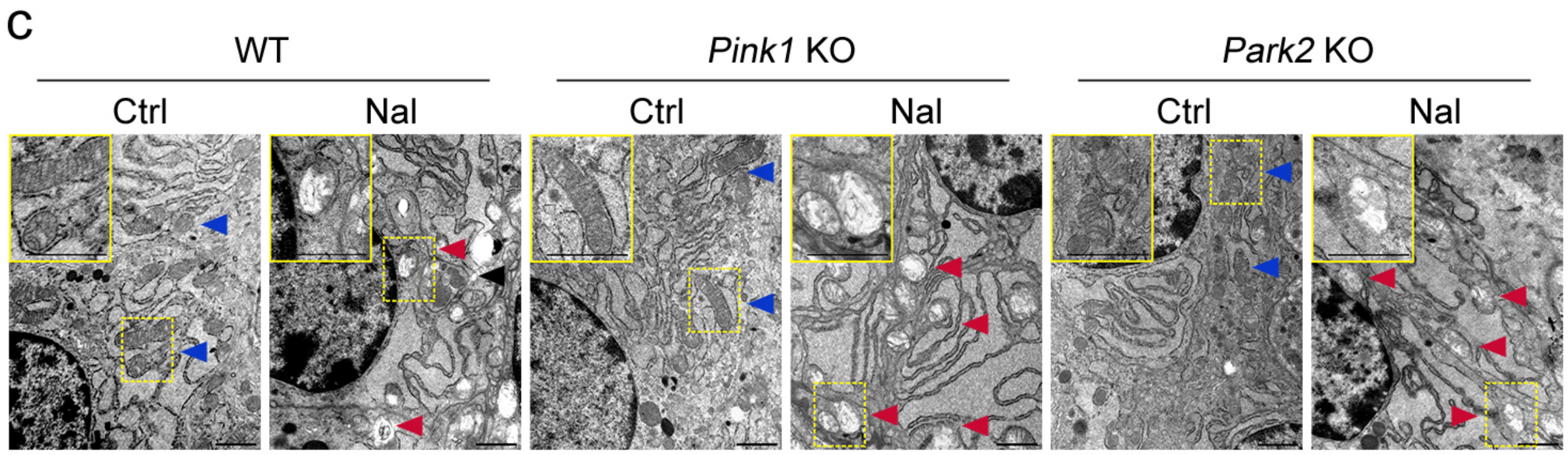

(c) Representative transmission electron microscopy images of mouse thyroid tissue from the indicated groups. Red arrows indicate damaged mitochondria (characterized by disrupted cristae and reduced cristae density), blue arrows indicate intact mitochondria, and black arrows indicate autophagosomes engulfing damaged mitochondria. Scale bar, 1 μm.

- In Figure 4H, there is a small typo - it should be K63, not K68

Response: We thank the reviewer for carefully catching this typo. The reviewer is correct that the ubiquitin linkage should be "K63" instead of "K68" in Figure 4H. We have corrected this error in the revised manuscript and have updated the figure accordingly. We apologize for any confusion this mistake may have caused.

- In Figure 5D, the authors assess mtDNA release into the cytosol. In the vector sample and the S722A sample, treated with NaI, there appears to be a grouping of mtDNA release, reminiscent of mtDNA nucleoids in Drp1 deficient cells (PMID 23821750). Is this something the authors have considered?

Response: We thank the reviewer for this insightful comment and for directing our attention to the interesting study by Ban-Ishihara et al. (PMID: 23821750). Indeed, the authors of that study observed that in Drp1-deficient cells, mitochondrial nucleoids cluster together within hyperfused mitochondria, forming large aggregates. We have carefully considered this observation in relation to our findings presented in Figure 5D.

While the morphological appearance of mtDNA signals in the S722A mutant cells under NaI treatment may superficially resemble the nucleoid clusters described in the PNAS paper, the underlying mechanisms and functional consequences are fundamentally distinct, as supported by evidence already presented in our original submission:

Subcellular Localization and Integrity: In the PNAS study, the clustered nucleoids were localized inside the matrix of enlarged, bulb-like mitochondria (mito-bulbs), as demonstrated by co-staining with inner membrane markers. In our model, the TAX1BP1 S722A mutation impairs mitophagy, leading to mtDNA release into the cytosol. In fact, in our original submission, we already performed co-staining of mtDNA (PicoGreen) with the mitochondrial marker MitoTracker (shown in Figure 5D, right panels). These images clearly show that in S722A cells treated with NaI, PicoGreen signals no longer co-localize with MitoTracker, confirming that the mtDNA foci represent cytosolic leakage rather than nucleoids enclosed within intact mitochondria. This is further quantified by our cytoplasmic qPCR data (Figure 5B-E).

Functional Outcome: The clustering of nucleoids within fused mitochondria in Drp1-deficient cells results in sequestration of cytochrome c within stacked cristae, leading to delayed apoptosis. In contrast, the accumulation of cytosolic mtDNA resulting from defective mitophagy (as seen in S722A cells) triggers the cGAS-STING inflammatory pathway (Figure 5F). Thus, while both conditions involve aberrant mtDNA distribution, they represent distinct facets of mitochondrial quality control: one affecting fission and cristae architecture (apoptosis regulation), and the other affecting autophagic clearance and innate immune signaling (inflammation).

We agree that the morphological similarity is intriguing, and it suggests that both severe mitochondrial hyperfusion (Drp1 loss) and defective autophagic clearance (S722A mutation) can lead to mtDNA aggregation. However, the critical difference—already demonstrated by our co-localization data in the original Figure 5D—lies in whether mtDNA remains sequestered within mitochondria (as in Drp1 deficiency) or is released into the cytosol (as in S722A cells).

To fully address this point, we have now incorporated the following discussion into the revised manuscript (Discussion section), which directly compares our findings with those of Ban-Ishihara et al.:

“The aggregation of mtDNA signals observed in TAX1BP1 S722A mutant cells superficially resembles the nucleoid clustering reported in Drp1-deficient cells, where mitochondrial fission is blocked and nucleoids accumulate within hyperfused mitochondria (Ban-Ishihara et al., 2013). However, the underlying mechanisms and functional consequences are fundamentally distinct. In Drp1-deficient cells, clustered nucleoids remain enclosed within the mitochondrial matrix, leading to sequestration of cytochrome c and delayed apoptosis. In contrast, the TAX1BP1 S722A mutation impairs mitophagy, resulting in the release of mtDNA into the cytosol, as evidenced by the loss of co-localization between PicoGreen-stained mtDNA and the mitochondrial marker MitoTracker. This cytosolic mtDNA then activates the cGAS-STING inflammatory axis. Thus, while both conditions produce aggregated mtDNA signals, they reflect two distinct facets of mitochondrial quality control, one involving fission and cristae architecture to regulate apoptosis, and the other involving autophagic clearance to drive innate immune signaling and inflammation. The morphological similarity suggests that both severe mitochondrial hyperfusion and defective mitophagy can lead to mtDNA aggregation, but the critical difference lies in whether mtDNA is retained within or released from mitochondria.”

We believe that this addition directly addresses the reviewer’s insightful comment and appropriately places our findings in the broader context of mitochondrial nucleoid dynamics.

Reviewer #2:

Comments:

In their study, Xie et al. describe a mechanism in which mitochondrial dysfunction in autoimmune thyroiditis (AT) leads to the cytoplasmic release of mtDNA, which in turn triggers the cGAS–STING pathway, resulting in inflammasome activation and the release of inflammatory cytokines that drive immune infiltration. In contrast, mitophagy activated by the ATM–CHK2–TAX1BP1 pathway acts as a protective mechanism that prevents the release of mtDNA. Using the iodine accelerated spontaneous autoimmune thyroiditis mouse model, the authors demonstrate that activation of the PINK1–Parkin pathway triggers mitophagy as an adaptive response to mitochondrial dysfunction and damage. Finally, they show that pharmacological inhibition of the STING pathway attenuates thyroiditis severity in the mouse model. They also propose that "enhancing mitophagy may be a helpful therapeutic strategy to disrupt the feed-forward cycle of inflammation and tissue damage".

The study is well designed, with an elegant set of carefully conducted experiments involving mouse models, in vitro experiments using a human thyroid cell line, and analyses of tissue derived from human patients. All statements and conclusions are well supported by the experimental results.

We sincerely thank the reviewer for the thoughtful and positive assessment of our work. We are pleased that the reviewer finds our study well designed, the experiments carefully conducted, and the conclusions well supported. We greatly appreciate the constructive comments and valuable suggestions, which have helped us improve the manuscript. In the revised version, we have carefully addressed all the points raised, and we believe these revisions have substantially enhanced the clarity and rigor of our study. We hope the revised manuscript now meets the reviewer's expectations. Thank you again for your time and expertise.

However, it should be noted that the authors do not cite previous studies that have already demonstrated the significance of the STING pathway in AT, e.g. Shen et al. J Transl Autoimmun. 2025 Mar 22;10:100284; Ding et al. Tissue Cell. 2024 Dec;91:102596. In particular, the most striking omission is the failure to cite the paper by Lee et al., published in Thyroid. 2024 Nov;34(11):1401–1413, who demonstrated that loss of Pink1 is a driver of Hashimoto's thyroiditis and is associated with mitochondrial abnormalities, mitophagy defects, and upregulation of DAMP related genes including NLRP3, STING, and NF κB, along with increased release of pro inflammatory cytokines that promote recruitment of immune cells to the thyroid. Moreover, Lee et al. concluded that "impaired mitophagy-induced inflammation by excessive mtROS and DAMPs may be an additional stressor that exacerbates the autoimmune response", a conclusion very similar to that stated by Xie et al.

Response: We sincerely thank the reviewer for the positive assessment of our work and for the thoughtful comments. We are grateful for the careful reading and for bringing these important publications to our attention.

We have now carefully reviewed the recommended articles: Shen et al. (J Transl Autoimmun. 2025), Ding et al. (Tissue Cell. 2024), and Lee et al. (Thyroid. 2024). We apologize for the omission of these relevant studies in our original submission.

We particularly appreciate the reviewer for pointing out the study by Lee et al., which convincingly demonstrates that loss of Pink1 drives Hashimoto's thyroiditis through mitochondrial abnormalities, mitophagy defects, and upregulation of DAMP-related genes including STING and NLRP3. The conclusion that "impaired mitophagy-induced inflammation

by excessive mtROS and DAMPs may be an additional stressor that exacerbates the autoimmune response" is indeed highly consistent with our findings.

In the revised manuscript, we have now cited all three studies in the Introduction and Discussion sections, and we have explicitly acknowledged the work by Lee et al. as a key prior study demonstrating the connection between PINK1/Parkin-mediated mitophagy and STING signaling in Hashimoto's thyroiditis.

We would also like to briefly highlight the unique contributions of our study beyond these prior reports. While Lee et al. established the association between PINK1 deficiency and mitophagy impairment in Hashimoto's thyroiditis, our study further uncovers the upstream regulatory mechanism by which the ATM-CHK2-TAX1BP1 axis controls mitophagy in response to mitochondrial stress. Specifically, we demonstrate that CHK2 directly phosphorylates TAX1BP1 at Ser722, a modification that promotes the recognition of ubiquitinated mitochondria and facilitates mitophagic clearance. Additionally, we provide direct evidence that defective mitophagy leads to mtDNA leakage into the cytosol, which serves as the direct trigger for cGAS-STING activation. Finally, we demonstrate that pharmacological STING inhibition with C-176 effectively attenuates disease progression, highlighting a potential therapeutic strategy.

We believe that incorporating these prior studies not only strengthens the context of our work but also highlights the distinct mechanistic insights we provide. We thank the reviewer again for this valuable feedback, which has helped us better position our study within the existing literature.

The manuscript lacks a clearly stated aim and hypothesis.

Response: We thank the reviewer for this constructive criticism. We agree that a clearly stated aim and hypothesis are essential for guiding the reader through the study. In the original submission, the objectives and hypothesis were implied but not explicitly articulated. To address this, we have now added a dedicated paragraph at the end of the Introduction section, which clearly states the aim and hypothesis of our study.

Specifically, we have added the following text (please see revised manuscript, Introduction, final paragraph):

"In this study, we aimed to investigate whether mitochondrial dysfunction-driven activation of the mtDNA-cGAS-STING inflammatory axis contributes to the pathogenesis of autoimmune thyroiditis, and to elucidate the upstream regulatory mechanism of mitophagy that counteracts this process. We hypothesized that in thyroid follicular epithelial cells, chronic oxidative stress associated with thyroid hormone synthesis leads to mitochondrial damage and mtDNA leakage into the cytosol, thereby activating the cGAS-STING pathway and promoting autoimmune inflammation. Furthermore, we hypothesized that the ATM-CHK2-TAX1BP1 axis activates PINK1/Parkin-dependent mitophagy to clear damaged mitochondria and prevent mtDNA release, thus protecting against disease progression. Finally, we tested whether pharmacological inhibition of STING could attenuate autoimmune thyroiditis in a mouse model."

We believe this addition now provides a clear and explicit statement of our study's aim and hypothesis, which should help guide the reader through the subsequent results. We thank the reviewer for this valuable suggestion to improve the clarity of our manuscript.

Line 260: "Confocal microscopy quantification of cytosolic dsDNA foci (PicoGreen) and mitochondrial mass (MitoTracker) showed significant accumulation of extramitochondrial DNA in cells expressing the phospho-deficient TAX1BP1 S722A mutant compared with WT ...": Did the authors observe such extramitochondrial DNA in tissues from AT mice or human patients?

Response: We thank the reviewer for this important question, which was also noted by Reviewer #1. To address this, we performed additional experiments to examine mtDNA leakage in both patient and mouse disease samples. Using cytoplasmic qPCR, we detected significant mtDNA leakage in thyroid tissues from autoimmune thyroiditis patients (compared to healthy controls) as well as in thyroid tissues from the ISAT mouse model (compared to control mice). These new data are now included in the revised manuscript as Supplementary Figure 7c-f (patients) and Supplementary Figure 7g-i (mouse model). Thus, extramitochondrial mtDNA is indeed present in both human and mouse disease tissues, consistent with our in vitro findings in S722A mutant cells. We believe these results directly address the reviewer's question.

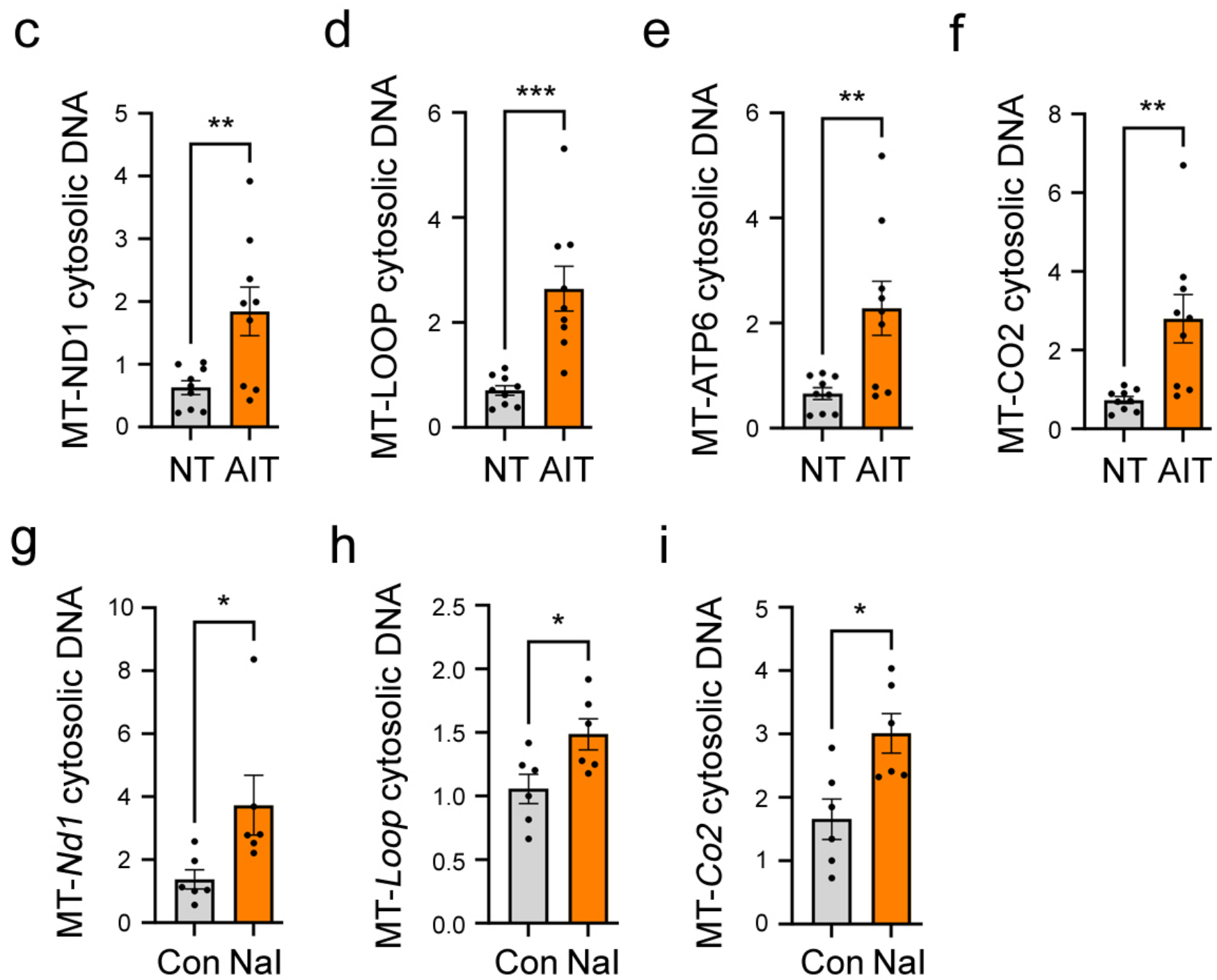

(c-f) Cytosolic mtDNA levels in thyroid tissues from healthy controls (NT) and AIT patients (AIT) (n = 9 per group). Specific mitochondrial DNA regions were quantified by qPCR: (c) *ND1*, (d) *LOOP*, (e) *ATP6*, and (f) *CO2*. Data are presented as mean ± SEM. $^{**}p < 0.01$; $^{***}p < 0.001$.
(g-i) Cytosolic mtDNA levels in thyroid tissues from control mice (Con) and ISAT mice at 6 weeks after high-iodine feeding (NaI) (n = 6 per group). Quantified regions: (g) *Nd1*, (h) *Loop*, and (i) *Co2*. Data are presented as mean ± SEM. $^{*}p < 0.05$.

Several methods are described only briefly. For example:
Line 456, Western blot and IP analysis: "Protein extraction from cultured cells was performed with IP lysis buffer followed by IP and immunoblotting using primary antibodies as described previously15." Reference 15 does not provide a detailed Western blot procedure (e.g. whether nitrocellulose or PVDF membranes were used, how membranes were blocked and incubated with antibodies, and how the signal was developed). Please provide the missing methodological details to enable reproducibility.

Response: We thank the reviewer for this important observation. We agree that sufficient methodological detail is essential for reproducibility. In the revised manuscript, we have substantially expanded the "Western blot and immunoprecipitation" subsection of Materials and Methods. The revised version now reads as follows:

"Cells were lysed with IP lysis buffer (50 mM Tris-Cl, pH 7.4, 1% Triton X 100, 1% NP 40, 150 mM NaCl, 1 mM EDTA, 0.25% sodium deoxycholate, and protease inhibitor cocktail), and lysates were centrifuged at 18,300×g for 20 min at 4 °C. The supernatants were collected for Western blot analysis or immunoprecipitation.

For Western blotting, protein samples were separated by SDS-PAGE and transferred onto PVDF membranes (Millipore). Membranes were blocked with 5% non fat milk in TBST (Tris buffered saline containing 0.1% Tween 20) for 1 h at room temperature, then incubated with primary antibodies (1:1000 dilution) overnight at 4 °C in blocking buffer. After three washes with TBST (10 min each), membranes were incubated with HRP conjugated secondary antibodies (1:5000 dilution) for 1 h at room temperature, followed by three additional washes. Immunoreactive bands were visualized using enhanced chemiluminescence (ECL) substrate (Tanon, 180-5001) and detected on a MicroChemi 4.2 imaging system (DNR). Band intensities were quantified using ImageJ (National Institutes of Health).

For immunoprecipitation (IP), primary antibodies were coupled to Protein A/G agarose beads (Santa Cruz Biotechnology) by gentle rotation for 2 h at 4 °C. The antibody bead complexes were then added to cell lysates and incubated overnight at 4 °C with rotation. Beads were washed three times with IP lysis buffer, and bound proteins were eluted by boiling in 2×SDS sample buffer for 5 min. Eluates were separated by SDS-PAGE and analyzed by immunoblotting as described above."

We believe these additions now provide a complete and reproducible description of our experimental procedures. We thank the reviewer again for prompting us to improve the clarity of our methods section.

Line 482: "Stable cell lines expressing mt-Keima were generated using a lentiviral system." Please provide a detailed description of this method.

Response: We thank the reviewer for requesting detailed methodology for the mt-Keima assay. We agree that a complete and accurate description is essential for reproducibility. In the revised manuscript, we have therefore substantially expanded the Materials and Methods section (under "Mito-Keima mitophagy assay"). The revised paragraph now reads as follows:

"Stable cell lines expressing mt-Keima were generated using a lentiviral system. The mt-Keima lentiviral particles were constructed and packaged by Hanbio Biotechnology (Shanghai, China). Nthy-ori3-1 cells were seeded in 6-well plates at a density of 2×10^5 cells per well and cultured overnight to reach 30-50% confluence. The culture medium was then replaced with fresh medium containing 5 μg/mL polybrene, and the cells were infected with the mt-Keima lentivirus at a multiplicity of infection (MOI) of 20. After 24 hours of infection, the virus-containing medium was removed and replaced with fresh complete medium. At 72 hours post-infection, stable transductants were selected by adding puromycin at a final concentration of 2 μg/mL for 7 days, with medium changes every 2-3 days. The expression of mt-Keima was confirmed by fluorescence microscopy (excitation at 458 nm and 561 nm). The established stable cell line was maintained in culture medium containing 1 μg/mL puromycin. For mitophagy analysis, cells were seeded in 6-well plates, allowed to recover for 24 hours, and then treated with NaI as indicated in the figure legends. After treatment, cells were harvested for flow cytometry (FACS) analysis."

We apologize for any confusion caused by the original description and believe that the revised version now provides full transparency and reproducibility. We thank the reviewer again for helping us improve the accuracy and clarity of our manuscript.

Figure 4h: Please correct the spelling error (K68 should be changed to K63).

Response: We thank the reviewer for the careful reading. The reviewer is correct that "K68" in Figure 4H should be "K63". We have corrected this typo in the revised manuscript and updated the figure accordingly. We apologize for any inconvenience caused by this mistake.

Point by Point Response to Reviewers:

Reviewer #2:

Comments:
Thank you for addressing all my comments and questions. The new data demonstrating the presence of extramitochondrial DNA in tissues from AT mice and human patients are clear and convincing.
However, a detailed description of the methods used to generate these data is still missing. Please provide a comprehensive description of the qPCR procedures, including DNA isolation methods, primer sequences, and the qPCR kits used, as well as the method employed for calculating the amount of detected DNA (e.g., absolute quantification, ΔΔCt, Pfaffl, or another approach).
In addition, please clarify the units used to represent DNA amounts in the plots shown in Figures 5a–e, Supplementary Figures 7c–i, and Supplementary Figures 9a–q and 9s–v. (This information can be provided either in the Methods section or in the figure legends).

Response: Thank you very much for your positive feedback and for the constructive suggestions to improve the methodological transparency of our study.

We have now provided a comprehensive description of the qPCR procedures in the revised Methods section. For your convenience, the complete revised Methods paragraph is provided below:

Detection of mtDNA in cytosolic extracts

Digitonin extracts from Nthy-ori3-1 cells were adapted from West et al.[40] as follows: siNC, siPINK1, and siParkin Nthy-ori3-1 cells exposed to 40mM NaI for 72h (1×10^7) were each divided into two equal aliquots, and one aliquot was resuspended in 500µl of 50µM NaOH and boiled for 30 minutes to solubilize DNA. 50µl of 1M Tris-HCl (pH 8.0) was added to neutralize the pH of the lysate, and the extracts served as normalization controls for total mtDNA. The second equal aliquot was resuspended in roughly 500µl buffer containing 150mM NaCl, 50mM HEPES pH7.4, and 20µg/ml digitonin (MedChemExpress). The homogenates were incubated end over end for 10 min to allow selective plasma membrane permeabilization, then centrifuged at 980g for 3 min three times to pellet intact cells. The cytosolic supernatants were transferred to fresh tubes and spun at 17000g for 10min to pellet any remaining cellular debris, yielding cytosolic preparations free of nuclear, mitochondrial and endoplasmic reticulum contamination. DNA was extracted from both the cytosolic fractions and the whole-cell normalization controls using the QIAQuick Nucleotide Removal Kit (QIAGEN) according to the manufacturer's protocol.

Quantitative PCR was performed using TB Green® Premix Ex Taq™ II (Tli RNaseH Plus, TaKaRa, Cat. No. RR820A) on a QuantStudio 6 Flex Real-Time PCR System (Roche). Each reaction was run in triplicate in a 20 µL volume containing 10 µL of 2× TB Green Premix, 0.4 µL each of forward and reverse primers (10 µM), 2 µL of template DNA, and 7.2 µL of RNase-free water. The thermal cycling conditions were: 93 °C for 1 min, followed by 45 cycles of 55 °C for 1 min and 72 °C for 1 min. A dissociation curve analysis was performed after each run to confirm amplification specificity.

The following primer pairs were used. For human targets: *ND1* (forward 5′-CACCCAAGAACAGGGTTTGT-3′, reverse 5′-TGGCCATGGGTATGTTGTTAA-3′); *LOOP* (forward 5′-CTATCACCCTATTAACCACTCA-3′, reverse 5′-TTCGCCTGTAATATTGAACGTA-3′); *ATP6* (forward 5′-AATCCAAGCCTACGTTTTCACA-3′, reverse 5′-AGTATGAGGAGCGTTATGGAGT-3′); *CO2* (forward 5′-AATCGAGTAGTACTCCCGATTG-3′, reverse 5′-TTCTAGGACGATGGGCATGAAA-3′). For mouse targets: *Nd1* (forward 5′-TCCGAGCATCTTATCCACGC-3′, reverse 5′-GTATGGTGGTACTCCCGCT-3′); *Loop* (forward 5′-AATCTACCATCCTCCGTGAAACC-3′, reverse 5′-TCAGTTTAGCTACCCCCAAGTTTAA-3′); *Co2* (forward 5′-ATAACCGAGTCGTTCTGCCAAT-3′, reverse 5′-TTTCAGAGCATTGGCCATAGAA-3′). The nuclear-encoded control gene *RPL13A* (human: forward 5′-GCCCTACGACAAGAAAAAGCG-3′, reverse 5′-TACTTCCAGCCAACCTCGTGA-3′; mouse: forward 5′-CTGCTCTCAAGGTTGTTCGGCT-3′, reverse 5′-CCTTCCGTTTCTCCTCCAGAGT-3′) was used to verify the absence of nuclear DNA contamination in the cytosolic fractions; no *RPL13A* signal was detected in any cytosolic sample.

The amount of mtDNA in the cytosolic fraction was normalized to total mtDNA from the whole-cell extract (the normalization control). Relative mtDNA levels were calculated using the ΔΔCt method. For each sample, the Ct value of the target mtDNA region was first normalized to the corresponding total mtDNA Ct value (ΔCt = Ct_target – Ct_total_mtDNA). The ΔΔCt was then calculated as ΔCt_treatment – ΔCt_control, and fold change was determined as 2^–ΔΔCt. Data are presented as fold change relative to the control group (mean of control group set to 1). The absence of nuclear DNA contamination was further confirmed by the lack of *RPL13A* amplification in the cytosolic fractions.

In addition, we have clarified the units in the figure legends of Figures 5a-e, Supplementary Figures 7c-i, and Supplementary Figures 9a-q and 9s-v by explicitly stating: "Data are presented as fold change relative to the control group (mean of control = 1)."

All changes have been highlighted in the revised manuscript. We believe the manuscript now fully meets the journal's standards for methodological transparency.

Thank you again for your valuable input.
