# Supplementary material for: Mitophagy mitigates mitochondrial DNA-induced activation of cGAS-STING in autoimmune thyroiditis
Source: Nat Commun. 2026 Jul 24;17:9041. doi: 10.1038/s41467-026-76047-9 (PMC13507228; doi:10.1038/s41467-026-76047-9)
Supplement: Supplementary file 3 [file 41467_2026_76047_MOESM3_ESM.pdf]

nature portfolio

Corresponding author(s): Xiaochen Xie, Weiping Teng, Zhongyan Shan, Liu Cao, Qiqiang Guo

Last updated by author(s): Jun 2, 2026

Reporting Summary

Nature Portfolio wishes to improve the reproducibility of the work that we publish. This form provides structure for consistency and transparency in reporting. For further information on Nature Portfolio policies, see our Editorial Policies and the Editorial Policy Checklist.

Statistics

For all statistical analyses, confirm that the following items are present in the figure legend, table legend, main text, or Methods section.

n/a	Confirmed	
☐	☒	The exact sample size (*n*) for each experimental group/condition, given as a discrete number and unit of measurement
☐	☒	A statement on whether measurements were taken from distinct samples or whether the same sample was measured repeatedly
☐	☒	The statistical test(s) used AND whether they are one- or two-sided<br>*Only common tests should be described solely by name; describe more complex techniques in the Methods section.*
☒	☐	A description of all covariates tested
☐	☒	A description of any assumptions or corrections, such as tests of normality and adjustment for multiple comparisons
☐	☒	A full description of the statistical parameters including central tendency (e.g. means) or other basic estimates (e.g. regression coefficient) AND variation (e.g. standard deviation) or associated estimates of uncertainty (e.g. confidence intervals)
☐	☒	For null hypothesis testing, the test statistic (e.g. *F*, *t*, *r*) with confidence intervals, effect sizes, degrees of freedom and *P* value noted<br>*Give P values as exact values whenever suitable.*
☒	☐	For Bayesian analysis, information on the choice of priors and Markov chain Monte Carlo settings
☒	☐	For hierarchical and complex designs, identification of the appropriate level for tests and full reporting of outcomes
☒	☐	Estimates of effect sizes (e.g. Cohen's *d*, Pearson's *r*), indicating how they were calculated

Our web collection on statistics for biologists contains articles on many of the points above.

Software and code

Policy information about availability of computer code

Data collection	No custom software was used for data collection. The following commercial instrument software packages were used:<br>Flow cytometry data were collected using BD FACSDiva software (version 8.0, BD Biosciences, San Jose, CA, USA).<br>Confocal microscopy images were acquired using NIS-Elements software (version 5.2, Nikon, Tokyo, Japan).<br>Transmission electron microscopy images were captured using the native JEOL software (version 2.1, JEOL, Tokyo, Japan).<br>Western blot images were captured using MicroChemi 4.2 system software (version 1.0, DNR Bio-Imaging Systems, Jerusalem, Israel).
Data analysis	The following software packages were used for data analysis:<br>Statistical analysis and graphing: GraphPad Prism 10.2.1 (GraphPad Software, Boston, MA, USA).<br>Flow cytometry data analysis: FlowJo 10.8.1 (BD Life Sciences, Ashland, OR, USA).<br>Image analysis (band intensity, PLA puncta, etc.): ImageJ 1.54p (National Institutes of Health, Bethesda, MD, USA).<br>No custom code or algorithm central to the conclusions was generated in this study.

For manuscripts utilizing custom algorithms or software that are central to the research but not yet described in published literature, software must be made available to editors and reviewers. We strongly encourage code deposition in a community repository (e.g. GitHub). See the Nature Portfolio guidelines for submitting code & software for further information.

Data

Policy information about availability of data

All manuscripts must include a data availability statement. This statement should provide the following information, where applicable:

- Accession codes, unique identifiers, or web links for publicly available datasets
- A description of any restrictions on data availability
- For clinical datasets or third party data, please ensure that the statement adheres to our policy

Source Data are provided with this paper. The mass spectrometry proteomics data have been deposited to the ProteomeXchange Consortium via the iProX partner repository with the dataset identifier PXD070578 [https://proteomecentral.proteomexchange.org/ui?view=datasets&search=PXD070578].

Research involving human participants, their data, or biological material

Policy information about studies with human participants or human data. See also policy information about sex, gender (identity/presentation), and sexual orientation and race, ethnicity and racism.

Reporting on sex and gender	The study included both male and female participants. Sex was determined based on self-reported medical records, and no gender-based analysis was performed because the study focused on molecular and pathological features of thyroiditis. Most participants were female, consistent with disease prevalence. No sex-based analyses were performed due to limited male sample size.
Reporting on race, ethnicity, or other socially relevant groupings	Race, ethnicity, and other socially relevant groupings were not considered in this study, as the research focused on molecular and cellular mechanisms of autoimmune thyroiditis without any social or demographic stratification.
Population characteristics	63 Hashimoto's thyroiditis patients and 40 healthy controls; age 21–65 years; diagnosis confirmed by clinical, serological and histopathological criteria. Healthy controls were individuals who underwent surgery for thyroid nodules and had no clinical diagnosis of thyroiditis.
Recruitment	All human thyroid specimens were obtained from the Department of Pathology at the First Affiliated Hospital of China Medical University. Tissues were collected from patients who underwent thyroidectomy for thyroid nodules. Patients were classified as having thyroiditis or healthy controls based on clinical and histopathological diagnosis. Samples were collected between 2019 and 2023. No specific recruitment strategies were applied. Selection based on diagnostic criteria and tissue availability.
Ethics oversight	Institutional Research Ethics Committee of the First Affiliated Hospital of China Medical University (approval number: 2023159)

Note that full information on the approval of the study protocol must also be provided in the manuscript.

Field-specific reporting

Please select the one below that is the best fit for your research. If you are not sure, read the appropriate sections before making your selection.

☒ Life sciences ☐ Behavioural & social sciences ☐ Ecological, evolutionary & environmental sciences

For a reference copy of the document with all sections, see nature.com/documents/nr-reporting-summary-flat.pdf

Life sciences study design

All studies must disclose on these points even when the disclosure is negative.

Sample size	For animal experiments, sample sizes (n = 6–8 per group) were determined based on our preliminary studies and prior publications to ensure adequate statistical power for detecting meaningful differences. No formal sample-size calculation was performed. For human tissue studies, all available archived specimens meeting the diagnostic criteria were included (63 Hashimoto's thyroiditis patients and 40 healthy controls), providing sufficient statistical power for correlation analyses. For in vitro experiments, three independent biological replicates were performed, a standard practice for cell-based assays to ensure reproducibility.
Data exclusions	No data were excluded from the analyses.
Replication	All experimental findings were successfully replicated. For in vitro experiments (e.g., Western blot, qPCR, confocal imaging, flow cytometry), each experiment was performed at least three independent times with similar results. For animal experiments, each mouse group contained 6–8 biologically independent animals, and the key findings (e.g., TEM quantification, ELISA, histology) were consistent across individual mice. For human IHC, samples from 63 patients and 40 controls were analyzed; the correlation patterns were robust and statistically significant.
Randomization	For animal experiments, mice were randomly assigned to control or high-iodine treatment groups. For human tissue studies, participants were not randomly assigned; samples were classified based on clinical and histopathological diagnosis (Hashimoto's thyroiditis vs. healthy controls). This non-randomized design is inherent to case-control studies and is justified by the study's objective to compare molecular features between disease and control groups.
Blinding	For human tissue studies, investigators were blinded to patient identity and clinical outcomes during immunohistochemical scoring and image

analysis. For animal studies, investigators were blinded to genotype and treatment group during TEM morphological quantification, histological scoring, and data analysis.

Reporting for specific materials, systems and methods

We require information from authors about some types of materials, experimental systems and methods used in many studies. Here, indicate whether each material, system or method listed is relevant to your study. If you are not sure if a list item applies to your research, read the appropriate section before selecting a response.

Materials & experimental systems

n/a	Involved in the study
☐	☒ Antibodies
☐	☒ Eukaryotic cell lines
☒	☐ Palaeontology and archaeology
☐	☒ Animals and other organisms
☐	☒ Clinical data
☒	☐ Dual use research of concern
☒	☐ Plants

Methods

n/a	Involved in the study
☒	☐ ChIP-seq
☐	☒ Flow cytometry
☒	☐ MRI-based neuroimaging

Antibodies

Antibodies used

Anti-PINK1 (#6946, CST, 1:1000), anti-Parkin (#4211, CST, 1:1000; sc-32282, Santa Cruz, 1:1000), anti-TOM20 (#42406, CST, 1:1000), anti-TIM23 (NBP2-13432, NOVUS, 1:1000), anti-FUNDC1 (NBP1-56430, NOVUS, 1:1000), anti-NIX (#12396, CST, 1:1000), anti-p-ATM S1981 (#13050, CST, 1:1000), anti-ATM (#2873, CST, 1:1000), anti-p-CHK2 T68 (#2661, CST, 1:1000), anti-CHK2 (#3440, CST, 1:1000), anti-STING (#13647, CST, 1:1000), anti-p-TBK1 S172 (#5483, CST, 1:1000), anti-TBK1 (#3504, CST, 1:1000), anti-p-IRF3 S396 (#29047, CST, 1:1000), anti-IRF3 (#4302, CST, 1:1000), anti-TAX1BP1 (#5105, CST, 1:1000), anti-NDP52 (#60732, CST, 1:1000), anti-OPTN (10837-1-AP, Proteintech, 1:1000), anti-p62 (P0067, Sigma, 1:2000), anti-ubiquitin (#3933, CST, 1:1000), anti-BAX (50599-2-Ig, Proteintech, 1:1000), anti-BAK (29552-1-AP, Proteintech, 1:1000), anti-α-tubulin (#2144, CST, 1:1000), anti-Flag (SG4110-16, Shanghai Genomics, 1:1000). Custom anti-p-TAX1BP1 (Ser722) was generated by the authors.

Validation

Anti-PINK1 (#6946, CST):https://www.cellsignal.cn/products/primary-antibodies/pink1-d8g3-rabbit-monoclonal-antibody/6946.
anti-Parkin (#4211, CST):https://www.cellsignal.cn/products/primary-antibodies/parkin-prk8-mouse-monoclonal-antibody/4211.
anti-Parkin (sc-32282, Santa Cruz): https://www.scbt.com/p/parkin-antibody-prk8.
anti-TOM20 (#42406, CST): https://www.cellsignal.cn/products/primary-antibodies/tom20-d8t4n-rabbit-monoclonal-antibody/42406.
anti-FUNDC1 (NBP1-56430, NOVUS): https://www.bio-techne.com/p/antibodies/fundc1-antibody_nbp1-56430.
anti-NIX (#12396, CST): https://www.cellsignal.cn/products/primary-antibodies/bnip3l-nix-d4r4b-rabbit-monoclonal-antibody/12396.
anti-p-ATM S1981 (#13050, CST): https://www.cellsignal.cn/products/primary-antibodies/phospho-atm-ser1981-d25e5-rabbit-monoclonal-antibody/13050.
anti-ATM (#2873, CST): https://www.cellsignal.cn/products/primary-antibodies/atm-d2e2-rabbit-monoclonal-antibody/2873.
anti-p-CHK2 T68 (#2661, CST): https://www.cellsignal.cn/products/primary-antibodies/phospho-chk2-thr68-antibody/2661.
anti-CHK2 (#3440, CST): https://www.cellsignal.cn/products/primary-antibodies/chk2-1c12-mouse-monoclonal-antibody/3440.
anti-STING (#13647, CST): https://www.cellsignal.cn/products/primary-antibodies/sting-d2p2f-rabbit-monoclonal-antibody/13647.
anti-p-TBK1 S172 (#5483, CST): https://www.cellsignal.cn/products/primary-antibodies/phospho-tbk1-nak-ser172-d52c2-rabbit-monoclonal-antibody/5483.
anti-TBK1 (#3504, CST): https://www.cellsignal.cn/products/primary-antibodies/tbk1-nak-d1b4-rabbit-monoclonal-antibody/3504.
anti-p-IRF3 S396 (#29047, CST): https://www.cellsignal.cn/products/primary-antibodies/phospho-irf-3-ser396-d6o1m-rabbit-monoclonal-antibody/29047.
anti-IRF3 (#4302, CST): https://www.cellsignal.cn/products/primary-antibodies/irf-3-d83b9-rabbit-monoclonal-antibody/4302.
anti-TAX1BP1 (#5105, CST): https://www.cellsignal.cn/products/primary-antibodies/tax1bp1-d1d5-rabbit-monoclonal-antibody/5105.
anti-NDP52 (#60732, CST): https://www.cellsignal.cn/products/primary-antibodies/ndp52-d1e4a-rabbit-monoclonal-antibody/60732.
anti-OPTN (10837-1-AP, Proteintech): https://www.ptgcn.com/products/OPTN-Antibody-10837-1-AP.htm.
anti-p62 (P0067, Sigma): https://www.sigmaaldrich.cn/CN/zh/product/sigma/p0067.
anti-BAX (50599-2-Ig, Proteintech): https://www.ptgcn.com/Products/BAX-Antibody-50599-2-Ig.htm.
anti-BAK (29552-1-AP, Proteintech): https://www.ptgcn.com/products/BAK-Antibody-29552-1-AP.htm.
anti-α-tubulin (#2144, CST): https://www.cellsignal.cn/products/primary-antibodies/alpha-tubulin-antibody/2144.
Specificity for anti-p-TAX1BP1 (Ser722) was confirmed using S722A mutants.

Eukaryotic cell lines

Policy information about cell lines and Sex and Gender in Research

Cell line source(s)

Nthy-ori3-1 cells were obtained from the European Collection of Authenticated Cell Cultures (ECACC). The cell line was derived from a 35-year-old female donor and immortalized by transfection with a plasmid encoding the SV40 large T antigen.

Authentication	The cell line was authenticated by the supplier using short tandem repeat (STR) profiling.
Mycoplasma contamination	All cells tested negative for mycoplasma contamination.
Commonly misidentified lines (See ICLAC register)	Nthy-ori3-1 is not listed in the ICLAC database of commonly misidentified cell lines.

Animals and other research organisms

Policy information about studies involving animals; ARRIVE guidelines recommended for reporting animal research, and Sex and Gender in Research

Laboratory animals	NOD.H-2h4 mice were obtained from the Jackson Laboratories (Bar Harbor, ME, USA). Pink1-deficient and Park2-deficient mice (C57BL/6N) were purchased from Cyagen Biosciences and backcrossed for more than 10 generations to NOD.H-2h4. Male and female NOD.H-2h4 wild-type, Pink1 knockout (Pink1−/−), and Park2 knockout (Park2−/−) mice were used (approximately 50% male, 50% female). Mice were 4–5 weeks of age at the start of the experiment. All animals were maintained under SPF conditions with a 12h light/dark cycle, at a controlled temperature of 22–24°C and relative humidity of 45–65%, and with free access to food and water. High-iodine water (0.05% sodium iodine) was administered ad libitum to induce autoimmune thyroiditis.
Wild animals	This study did not involve wild animals.
Reporting on sex	Both male and female mice were included in the study, with approximately equal representation. No sex-disaggregated analyses were performed because the primary objective was to evaluate the role of mitophagy in thyroiditis pathogenesis, and the sample size was not powered for sex-stratified comparisons.
Field-collected samples	This study did not involve samples collected from the field.
Ethics oversight	All animal experiments were approved by the Institutional Animal Care and Use Committee (IACUC) of China Medical University (approval number: CMUXN2023029). Animal care and experimental procedures were performed in accordance with the institutional guidelines and regulations. At the end of the experiments, all mice were euthanized by cervical dislocation, in accordance with the institutional animal care and use guidelines.

Note that full information on the approval of the study protocol must also be provided in the manuscript.

Clinical data

Policy information about clinical studies

All manuscripts should comply with the ICMJE guidelines for publication of clinical research and a completed CONSORT checklist must be included with all submissions.

Clinical trial registration	Not applicable. This study is not a clinical trial.
Study protocol	Not applicable. No clinical trial protocol was involved.
Data collection	Human thyroid specimens were obtained from the Department of Pathology at the First Affiliated Hospital of China Medical University. Tissues were collected from patients who underwent thyroidectomy for thyroid nodules between 2019 and 2023. Samples were classified into two groups based on clinical and histopathological diagnosis: Hashimoto's thyroiditis (n = 63) and healthy controls (n = 40, individuals without any clinical diagnosis of thyroiditis undergoing surgery for benign nodules). No specific recruitment or intervention was applied.
Outcomes	Not applicable.

Plants

Seed stocks	Not applicable.
Novel plant genotypes	Not applicable.
Authentication	Not applicable.

Flow Cytometry

Plots

Confirm that:

- [x] The axis labels state the marker and fluorochrome used (e.g. CD4-FITC).
- [x] The axis scales are clearly visible. Include numbers along axes only for bottom left plot of group (a 'group' is an analysis of identical markers).
- [x] All plots are contour plots with outliers or pseudocolor plots.
- [x] A numerical value for number of cells or percentage (with statistics) is provided.

Methodology

Sample preparation	Nthy-ori3-1 cells stably expressing mt-Keima were seeded in 6-well plates, allowed to recover for 24 h, and then treated with NaI (40 mM) for 24 h as indicated. Cells were harvested by trypsinization, washed once with PBS, and resuspended in FACS buffer. No fixation or permeabilization was performed.
Instrument	Cells were analyzed on a BD FACSymphony A1 cell analyzer (BD Biosciences).
Software	Data acquisition was performed using BD FACSDiva software (BD Biosciences). Analysis of flow cytometry data was performed using FlowJo software (BD Life Sciences).
Cell population abundance	No cell sorting was performed.
Gating strategy	Cells were first gated on forward scatter (FSC) versus side scatter (SSC) to exclude debris and doublets. Subsequently, live single cells were analyzed for mt-Keima fluorescence. Mitophagy was quantified by the ratio of 561 nm (acidic pH) to 405 nm (neutral pH) fluorescence. Boundaries between “positive” and “negative” populations were defined using untreated control cells as reference.

- [x] Tick this box to confirm that a figure exemplifying the gating strategy is provided in the Supplementary Information.
